# The bank of swimming organisms at the micron scale (BOSO-Micro)

**DOI:** 10.1371/journal.pone.0252291

**Published:** 2021-06-10

**Authors:** Marcos F. Velho Rodrigues, Maciej Lisicki, Eric Lauga

**Affiliations:** 1 Department of Applied Mathematics and Theoretical Physics, University of Cambridge, Cambridge, United Kingdom; 2 Faculty of Physics, University of Warsaw, Warsaw, Poland; Texas A&M University, UNITED STATES

## Abstract

Unicellular microscopic organisms living in aqueous environments outnumber all other creatures on Earth. A large proportion of them are able to self-propel in fluids with a vast diversity of swimming gaits and motility patterns. In this paper we present a biophysical survey of the available experimental data produced to date on the characteristics of motile behaviour in unicellular microswimmers. We assemble from the available literature empirical data on the motility of four broad categories of organisms: bacteria (and archaea), flagellated eukaryotes, spermatozoa and ciliates. Whenever possible, we gather the following biological, morphological, kinematic and dynamical parameters: species, geometry and size of the organisms, swimming speeds, actuation frequencies, actuation amplitudes, number of flagella and properties of the surrounding fluid. We then organise the data using the established fluid mechanics principles for propulsion at low Reynolds number. Specifically, we use theoretical biophysical models for the locomotion of cells within the same taxonomic groups of organisms as a means of rationalising the raw material we have assembled, while demonstrating the variability for organisms of different species within the same group. The material gathered in our work is an attempt to summarise the available experimental data in the field, providing a convenient and practical reference point for future studies.

## 1 Introduction

Swimming microorganisms were first observed almost 350 years ago by Antonie van Leeuwenhoek [1]. Since then, extensive knowledge has been obtained on their form, function, genetics and behaviour [2]. We now also understand the vital role they play in ecosystems [3] as well as in the individual organisms they can inhabit, and whose health they influence [4]. Their ubiquity demonstrates an astonishing diversity and adaptability to the most extreme conditions. Furthermore, the involvement of swimming microorganisms in biological processes, irrespective of habitat, is invariably and directly linked to their motility. The chance of a ciliate escaping a predator [5, 6], the capacity of a spermatozoon to enter and fertilise an egg [7], and the virulent spreading of pathogenic bacteria [8] are but a few examples of how cell motility can be decisive for survival.

Swimming in a fluid on small, cellular length scales is subject to the physical constraints imposed by the viscosity of the fluid. With typical lengths of the order of microns, and speeds of a few to hundreds of microns per second, the fluid flows set up by microswimmers are characterised by negligibly small Reynolds numbers. The world in which their locomotion takes place is therefore dominated by viscous friction and the effects of inertia are unimportant [9–11]. As a result, the propulsion strategies employed by larger organisms such as fish, mammals, insects and birds are ineffective on cellular length and time scales [12–18].

Swimming microorganisms have thus developed physical mechanisms to successfully overcome, and in fact exploit, viscous drag by actuating slender tail-like appendages called flagella [19]. Somewhat confusingly, the same name is used to refer to either the polymeric filaments of prokaryotes or the more complex, muscle-like flexible organelles of eukaryotes. In the former case, the filaments are semi-rigid and helical, and they are rotated passively by molecular motors embedded in the cell wall [20]. For the latter, the flagella undergo three-dimensional active motion resulting from the action of internally-distributed motor proteins [2]. Despite the variation in structure, distribution and beating pattern of flagella between species, the actuation of flagella in a viscous fluid provides the unifying biophysical picture through which the locomotion of all microorganisms can be understood.

Assessing how fast a certain microorganism can swim is not a simple task. Motility is strongly dependent on temperature [21–24] and on the viscosity of the medium in which the cells swim [24–28]. Absolute pressure [29], pH [30] and even magnetic field [31] have also been shown to influence the motility of certain species. The motile behaviour of microorganisms may also change depending on whether they are undertaking the role of prey or predator [5, 6, 32, 33]. Furthermore, cellular propulsion also depends on biochemical factors [34, 35]. Swimming speeds for different species within the same genus (e.g. *Vibrio*, *Ceratium*, *Peridinium* and *Paramecium*) and even different strains of the same species (e.g. *Escherichia coli* [36, 37], *Campylobacter jejuni* [26] and *Pseudomonas aeruginosa* [38]) are available in the literature but little information is given on the variability of the swimming speed within a species or even for an individual organism. Overall, data on the swimming speed variability of different organisms are rather scarce. Our recent study for eukaryotic microswimmers has shown that some of the swimming speed distributions have a universal character when appropriately re-scaled [39] but the lack of data limits a more detailed analysis. Since motility may be the key factor distinguishing between the regimes of cell feeding (i.e. advective vs diffusive) or sensing (e.g. spatial vs temporal) [40], extensive data on swimming might aid elucidating the physical mechanisms affecting the cell behaviour.

The biophysical description of cellular propulsion was pioneered in the last century with the works of Gray (from the biology side) [41] and Taylor (mathematics) [42], and it has now grown into a mature field of research [10, 20, 43–48]. Despite many theoretical advances, the difficulties of observation and measurement on small scales, as well as the complexity of the fluctuating fluid flows continue to offer outstanding challenges for detailed studies. In addition, the locomotion of cells links to the rapidly growing field of artificial active matter, addressing the question of how microbiology, medicine and robotics could work together for the creation and manipulation of artificial swimmers, some of which are inspired by flagellated organisms [49]. These laboratory swimmers have a promising potential to perform site-specific drug deliveries, or chemical sensing, and to assist micro-manipulations in advanced surgery, enhancing the effectiveness of medical treatments [50–53].

Motivated by the combination of current activity in the research field and its rich scientific history, we carry out in this paper a biophysical survey of the available experimental data produced to date (13 April 2021) on the characteristics of motile behaviour in unicellular microswimmers. Specifically, we assemble from the available published literature empirical data on the motility of four broad categories of organisms, namely bacteria (and archaea), flagellated eukaryotes, spermatozoa and ciliates. Whenever possible, we gather a broad set of parameters related to biological, morphological, kinematic and dynamical aspects of the swimming cells: species, geometry and size of the organisms, swimming speeds, actuation frequencies and amplitudes, number of flagella and properties of the surrounding fluid. We assemble our results in a large downloadable database that we call BOSO-Micro, with BOSO standing for “Bank Of Swimming Organisms” and “Micro” emphasising their microscopic scale.

We then analyse the data from the database in light of the established fluid mechanics principles for propulsion at low Reynolds number in order to sort and organise the assembled raw material. We reproduce classical scalings for the locomotion of cells within the same taxonomic groups, while demonstrating the variability between different species within the same group. The resulting database, which is made available with this paper and downloadable from the Center for Open Science (OSF) repository, provides a convenient and practical reference point for future studies [54]. Despite our best efforts, some species and studies may have been left out of our dataset, and since research in the field is active and ongoing, it is important to also allow our database to be easily and continuously extended. To allow future collaborative effort of the community, we have also organised an open source version of the database on GitHub [55], which can be supplemented with new data while retaining a version control.

The paper is organised as follows. In Section 2, we describe in detail the structure of the database, its sources, and the procedures used for data selection, extraction and processing. We also briefly outline the theoretical basis of locomotion at low Reynolds number that serves as a guide for the exploration of our data. We then present and discuss the collected data, separating them according to the different taxonomic groups: bacteria and archaea (Sec. 3), flagellated eukaryotes (Sec. 4), spermatozoa (Sec. 5) and ciliates (Sec. 6). We summarise the findings in Sec. 7, where we also comment on the potential caveats and limitations of our work. We conclude the paper by displaying the complete database in Appendix A.

## 2 Methods

### 2.1 Propulsion at low Reynolds number

Cellular swimming is invariably coupled to the fluid mechanics of the surrounding environment. Biological locomotion in aqueous media happens on a wide range of spatial scales, from sub-micrometre bacteria to whales measuring tens of metres. In all cases, steady swimming results from balancing the propulsive forces generated by the moving swimmer with the frictional (drag) forces from the surrounding environment [9, 10]. Propulsion results from the biological actuation, which always involves motion of the body relative to the fluid. This in turn generates flow, which dissipates energy and thus resists the motion.

For biological locomotion in Newtonian fluids, the fluid flow around a swimming organism is governed by the Navier-Stokes equations. However, in the regime of interest for this work, the effects of viscosity on the motion typically dominate inertial effects, as classically quantified by the dimensionless Reynolds number. Assuming *U* to be the typical speed scale of a swimmer of a characteristic size *B*, moving through a fluid of mass density *ρ* and dynamic viscosity *η*, the ratio of inertial to viscous forces is defined as the (steady) Reynolds number, Re = *ρUB*/*η*. Because the propulsion mechanism often involves the periodic motion of biological organelles of characteristic length ℓ and angular frequency *ω*, another dimensionless number can be constructed, termed the oscillatory Reynolds number and defined as Re_*ω*_ = *ρωℓ*^2^/*η*.

In Table 1 we estimate both values of Re and Re_*ω*_ for a number of representative organisms from the database assuming their environment to be water at 25°C. In the majority of cases, these estimates suggest that it is appropriate to neglect all inertial effects when compared to viscous forces, as both Re ≪ 1 and Re_*ω*_ ≪ 1, or at most just below one. To interpret the dynamics of microswimmers, it is thus appropriate to consider the over-damped limit, when the fluid dynamics are governed by the steady Stokes equations. For a detailed overview of the fluid dynamics of locomotion at low Reynolds we refer to classical work in Refs. [10, 19, 56–58].

**Table 1:** Steady (Re) and oscillatory (Re_*ω*_) Reynolds numbers for five representative organisms from the database. The values of the mass density (*ρ*) and dynamic viscosity (*η*) used correspond to water at 25°C.

Species	*B* [*μ*m]	*U* [*μ*m s^−1^]	*ω* [rad s^−1^]	ℓ [*μ*m]	Re	Re_*ω*_
*E. coli* (bacteria)	2.5	24.1	823.1	8.3	6.7510^−5^	6.3510^−2^
*H. salinarum* (archaea)	2.6	3.3	144.5	4.3	9.6110^−6^	2.9910^−3^
*G. lamblia* (flag. eukaryote)	11.3	26	81.7	11.6	3.2810^−4^	1.2210^−2^
Bull spermatozoon (Metazoa)	8.9	97	129.2	54.0	9.6410^−4^	4.2210^−1^
*P. caudatum* (ciliate)	242	1476.5	197.3	12	4.0010^−1^	3.1810^−2^

### 2.2 Data collection and processing

In this paper we focus on unicellular microorganisms that can swim on their own, either using the actuation of flagella and cilia or by periodic deformations of their cell bodies, so that they generate net displacements via interactions with the surrounding fluid. We therefore do not include gliding and twitching motility, nor amoeboid displacement. Swarming bacteria were however included, because swarmer cells are also swimmer cells.

In order to identify in the available literature the swimming characteristics of multiple organisms, we selected six seminal biophysical papers in the field of biological fluid dynamics of microscale locomotion (ordered by year of publication): (i) an early analysis of microscale swimming by Taylor [42]; (ii) the work of Gray and Hancock on the swimming of spermatozoa [59]; (iii) the lecture on the theory of flagellar hydrodynamics by Lighthill [56]; (iv) the introduction to life at low Reynolds number by Purcell [9]; (v) the classical review paper on locomotion by cilia and flagella by Brennen and Winet [19]; and (vi) the study on bacterial locomotion in viscous environments by Berg and Turner [60]. These papers are commonly viewed by the community as groundbreaking biophysical contributions to the field of microswimmer hydrodynamics, which is reflected in the number of citations of these works, summing up to over 5300. The respective numbers of citations are: 614 [19]; 240 [60]; 733 [59]; 541 [56]; 2461 [9]; 736 [42]. Source: Web of Knowledge, 13 April 2021.

In order to construct the database, we first used the Web of Knowledge database to assemble two lists of published references: (a) papers that are cited by any of the six source papers, (b) papers that cite any of the six source papers. Each of the resulting references was then examined to determine whether it contained any measurements or reports on the swimming characteristics of any unicellular microswimmer, or if it led to other useful references. We acknowledge that our selection of six initial papers is clearly biased towards the fluid mechanics and biophysical aspects, yet we hope that by a thorough query of the cited and citing papers we managed to sufficiently extend the scope of the search to construct a comprehensive and relevant dataset. In order to allow further extension of the database to include new and possibly omitted studies, we refer to the open GitHub version of it [55]. Note that we reproduce all the collected information in the form of tables in Appendix A, in which we list all relevant material in a concise form.

**Fig 1:**
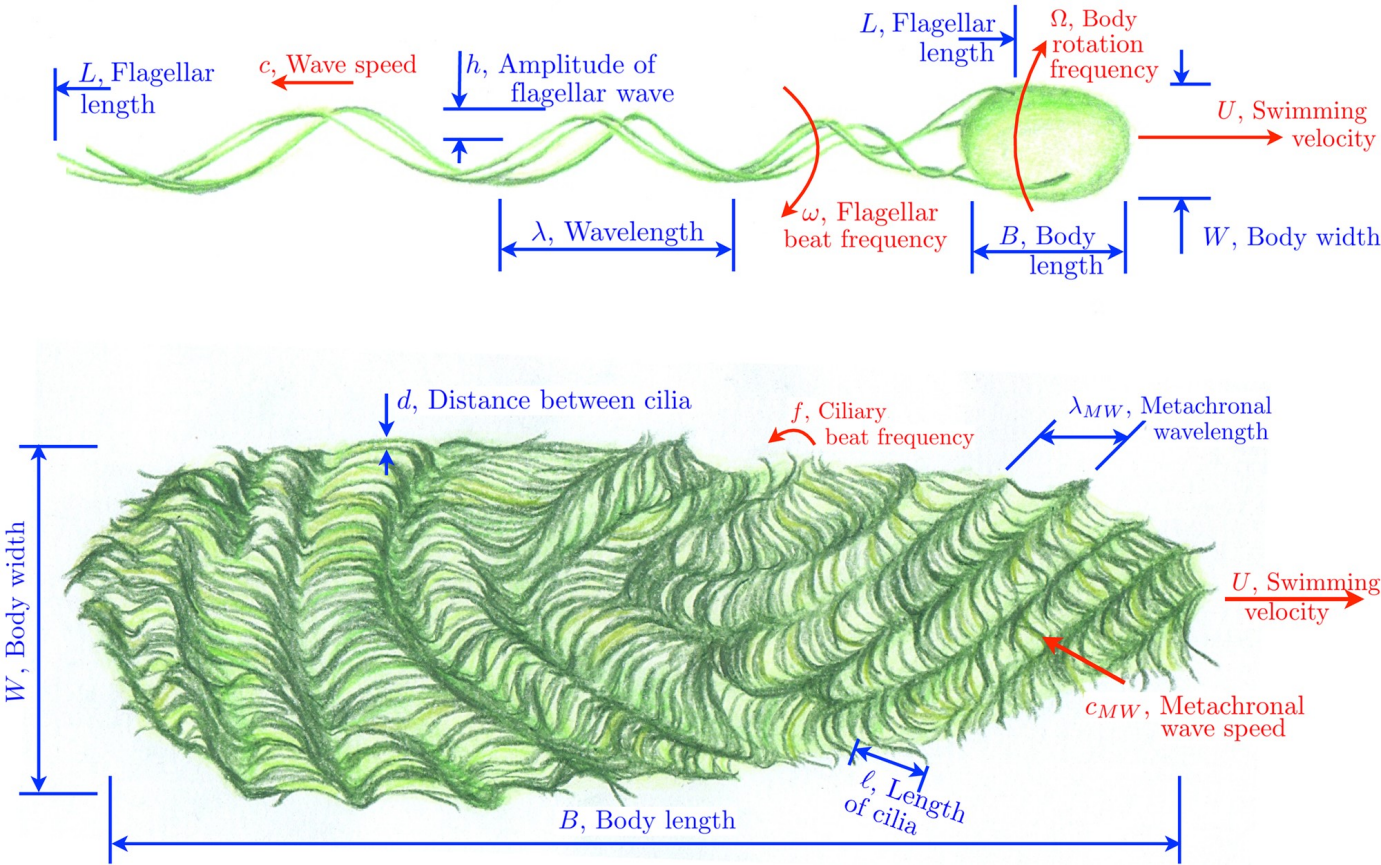
Top: Geometrical and kinematic parameters of flagellated swimmers, illustrated here for a bacterium; we use the same symbols for cells employing planar or helical waves for simplicity. Bottom: Geometrical and kinematic parameters of ciliated swimmers. Drawings by Marcos F. Velho Rodrigues.

In addition to the cell swimming speed, we extracted other geometrical and kinematic characteristics of the organisms when available in experimental studies. These parameters are summarised on the sketches in Fig 1 for cells with a small number of flagella (top) and for cells with many appendages (bottom): dimensions of cell bodies, swimming speeds, lengths and beat frequencies of cilia and flagella, wavelengths, wave speeds, amplitudes and form of the propagated waves (two or three-dimensional, sinusoidal, helicoidal or complex patterns for flagella, and metachrony for cilia [61]). Note that several works exist that review solely the morphological features of swimming microorganisms [62–64]. As the focus of our paper is on the relationship between geometry, kinematics and locomotion, we chose not to include in our database any study that does not report any swimming speeds.

**Fig 2:**
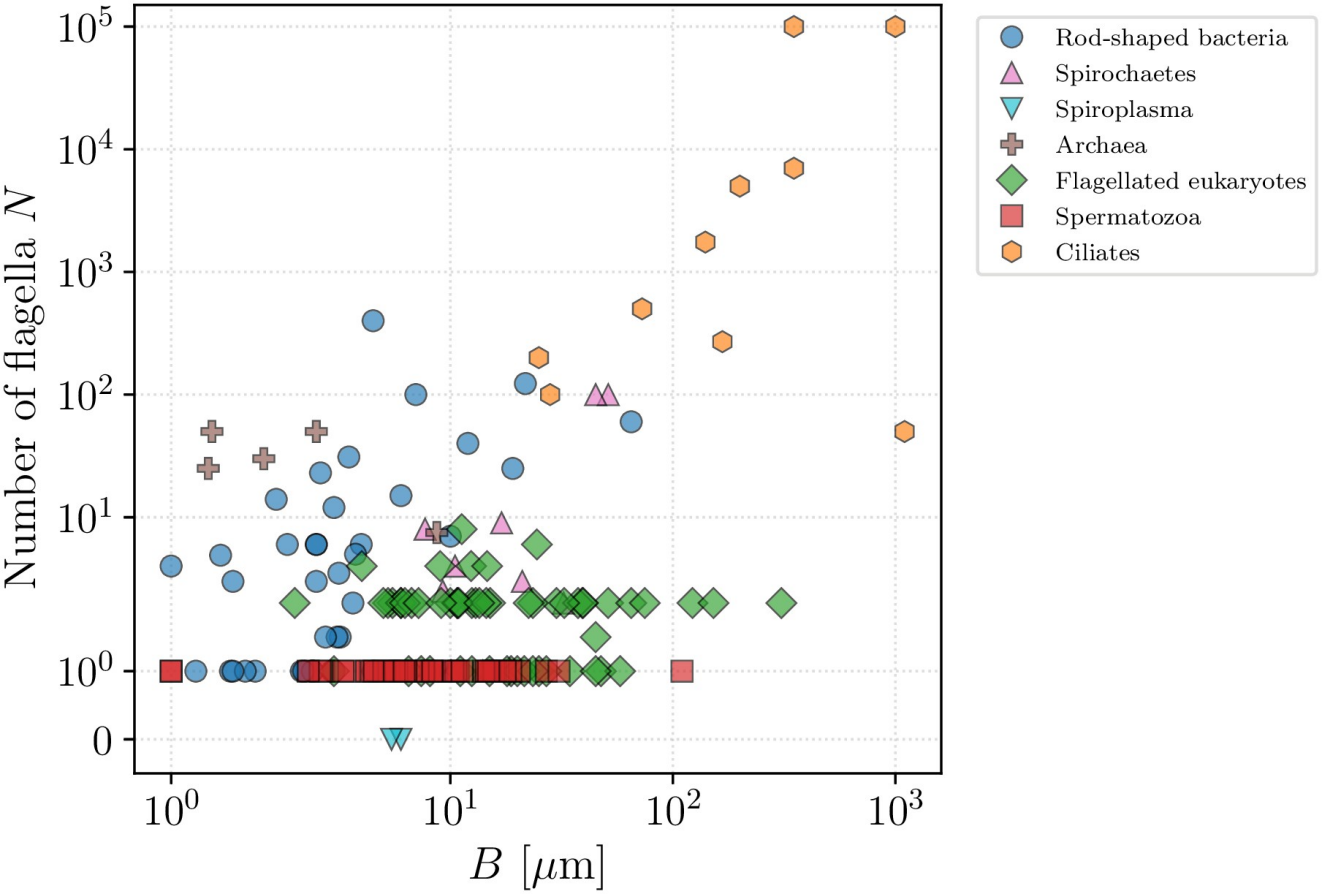
Number of appendages, i.e. cilia or flagella, of each organism (whenever available) plotted against the cell body length. Both characteristics span orders of magnitude but the data cluster within taxonomic groups.

In all, the database contains a total of 382 species for which we were able to find at least one measurement on swimming speed along with other characteristics. Within the tree of life, microswimmers of these species are present in all domains: Bacteria and Archaea (together encompassing prokaryotic organisms), and Eukaryota (including flagellated and ciliated cells and the spermatozoa of multicellular organisms). Members of these different groups clearly differ in size, propulsion modes and other physical characteristics. In particular, we plot in Fig 2 the number of flagella (or cilia) of each organism against the typical cell body length, demonstrating the partial clustering of organisms within their taxonomic groups. On top of variability within taxa, there is a considerable diversity even within groups, and both parameters can span several orders of magnitude. Bearing this in mind, we analyse each taxonomic group separately in what follows.

**Fig 3:**
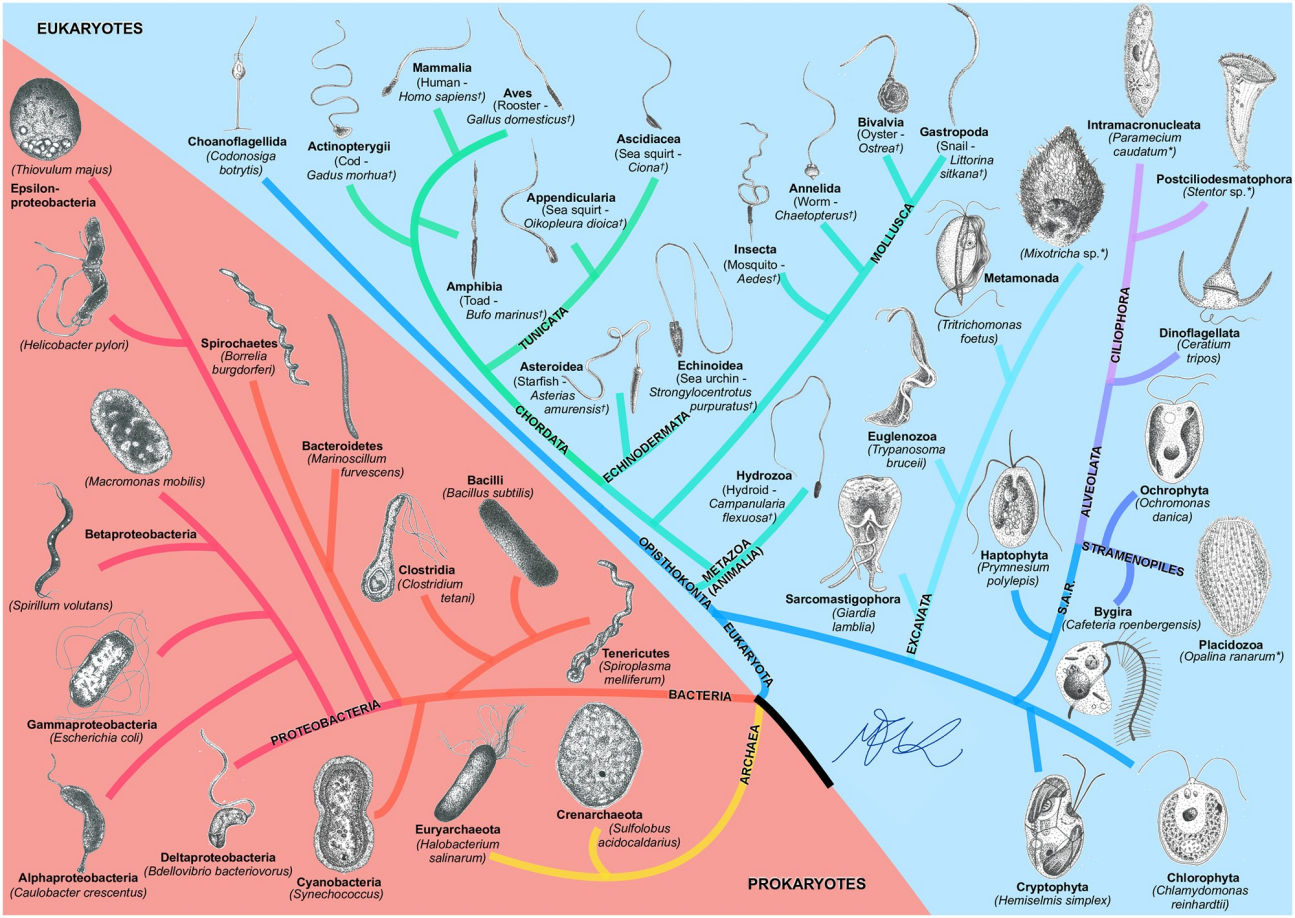
BOSO-Micro Tree of Life. The taxonomy was obtained from the Open Tree of Life [65]. Ciliates are indicated by an asterisk *, and spermatozoa by a dagger ^†^ beside their species’ names. The drawings are not to scale and were inspired by real microscopy images or by illustrations. All drawings by Marcos F. Velho Rodrigues.

In order to help visualise the range of the present study, we also follow taxonomy as presented in the Open Tree of Life [65] and sketch in Fig 3 the various phylogenetic branches included in our work together with a drawing of one representative organism within each phylum covered.

## 3 Bacteria and archaea

We start our journey through swimming microorganisms with prokaryotes, namely the domains Bacteria and Archaea. Bacteria constitute the bulk of the biomass on Earth, inhabiting the soil, water reservoirs, and the guts of larger organisms. They are simple cells without a nucleus, yet they display a remarkable diversity of shapes [66]. Motility is a crucial feature for many species of bacteria, in particular for nutrition purposes, and to this end bacteria have developed various propulsion strategies [67].

Two broad categories of swimming bacteria exist. In the first one, propulsion is enabled by the actuated motion of flagella located in the fluid outside the cell body [20]. Unlike their active eukaryotic analogues, prokaryotic flagellar filaments are passive organelles [68] of typical length of a few microns, attached to a flexible hook that acts as a joint connected to a molecular motor embedded in the cell wall. The word flagellum (plural flagella) is used to refer to the motor–hook–filament complex. The bacterial rotary motor, driven internally by ion fluxes, exerts a torque on the hook, which transmits it to the filament thereby inducing its rotational motion. Because the flagellar filaments have helical shapes, their rotation in a viscous fluid induces a hydrodynamic propulsive force and leads to the motion of the organism [10].

Flagellated bacteria can be equipped with anything from one flagellum (monotrichous cells) to a few flagella originating from different points on the cell body [69]. Polar bacteria have their flagella positioned in the vicinity of the pole of the cell. Other arrangements are seen in lophotrichous (a tuft of flagella at the pole) and amphitrichous (flagella at each pole) cells, while for peritrichous species (including the well-studied model organism *Escherichia coli*) the rotary motors are located approximately randomly on the cell body.

Some species of flagellated bacteria can also display a mode of motility named swarming, where cells undergo changes in morphology and rely on intercellular interactions to move near surfaces [70]. Some species can transition from swimming to swarming behaviours by relying on polar flagella for swimming, while exploiting several flagella distributed along the sides of their bodies for swarming [71]. The data for most bacteria in our database is presented in Table 4.

In the second type of bacterial swimming, cells move via a time-dependent deformation of their body. Famously, cells in the phylum Spirochaetes are morphologically distinguished by having internal axial flagellar filaments running lengthwise between the inner and outer membrane of their periplasmatic space, producing helical waves in the cell body with no apparent slippage with respect to the surrounding fluid [72]. Unlike typical rod-shaped bacteria, this particular configuration allows them to swim in extremely viscous gel-like media.

Finally, cells in the genus *Spiroplasma* do not present axial flagellar filaments. Instead, they swim by propagating kink pairs along their helical body using the motion of its cytoskeleton. This creates a processive change in the helicity of the body, which also allows them to move through extremely viscous fluids [73]. Our data for spirochaetes and *Spiroplasma* is presented in Table 5.

Relatively less studied are the species in the prokaryotic domain Archaea. Archaea have morphologies similar to bacteria but, equipped with a different molecular organisation, they are able to live under conditions that are extreme and hostile to other forms of life. Other differences exist; for example, some species of archaea have square-shaped bodies, unlike any bacterium or eukaryote [74, 75]. Although the actuation of archaeal flagella has been characterised in detail [76], the motile behaviour of only about 10 species in the whole domain has been studied so far, with all data summarised in Table 6.

### 3.1 Geometry and swimming speeds of the cells

**Fig 4:**
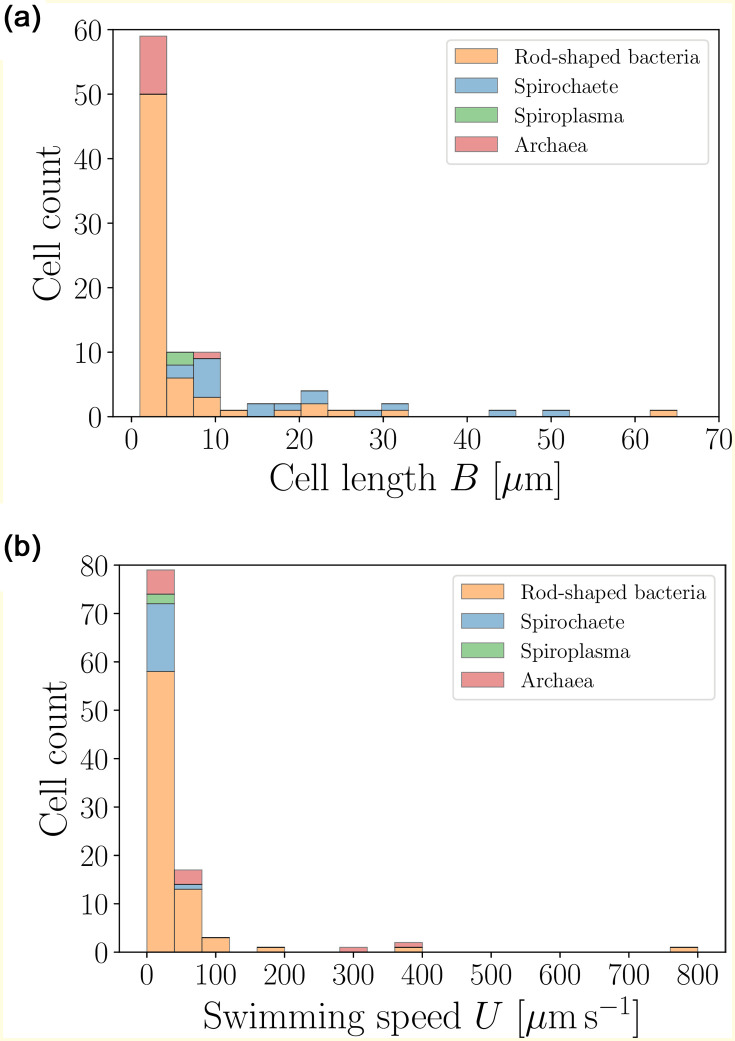
Histograms of body lengths, *B* (*μ*m, left), and swimming speeds, *U* (*μ*m s^−1^, right), for rod-shaped bacteria (excluding spirochaetes and *Spiroplasma*) (〈*B*〉 = 5.79 ± 9.33 *μ*m (*n* = 66), 〈*U*〉 = 48.33 ± 98.47 *μ*m s^−1^ (*n* = 77)), spirochaetes (〈*B*〉 = 18.59 ± 13.02 *μ*m (*n* = 17), 〈*U*〉 = 17.94 ± 18.84 *μ*m s^−1^ (*n* = 15)), *Spiroplasma* (〈*B*〉 = 5.72 ± 0.28 *μ*m (*n* = 2), 〈*U*〉 = 1.69 ± 0.81 *μ*m s^−1^ (*n* = 2)) and archaea (〈*B*〉 = 2.71 ± 2.12 *μ*m (*n* = 10), 〈*U*〉 = 89.18 ± 126.57 *μ*m s^−1^ (*n* = 10)) from our database. Most organisms have sizes below 10 *μ*m (〈*B*〉 = 7.75 ± 10.85 *μ*m (*n* = 95)) and swimming speeds below 100 *μ*m s^−1^ (〈*U*〉 = 46.98 ± 95.42 *μ*m s^−1^ (*n* = 104)).

The distribution of sizes and speeds of the prokaryotes from Tables 4–6 are shown in Fig 4. The characteristic length of the cell bodies does not exceed 10 *μ*m while the typical swimming speeds are of the order of tens of *μ*m s^−1^.

**Fig 5:**
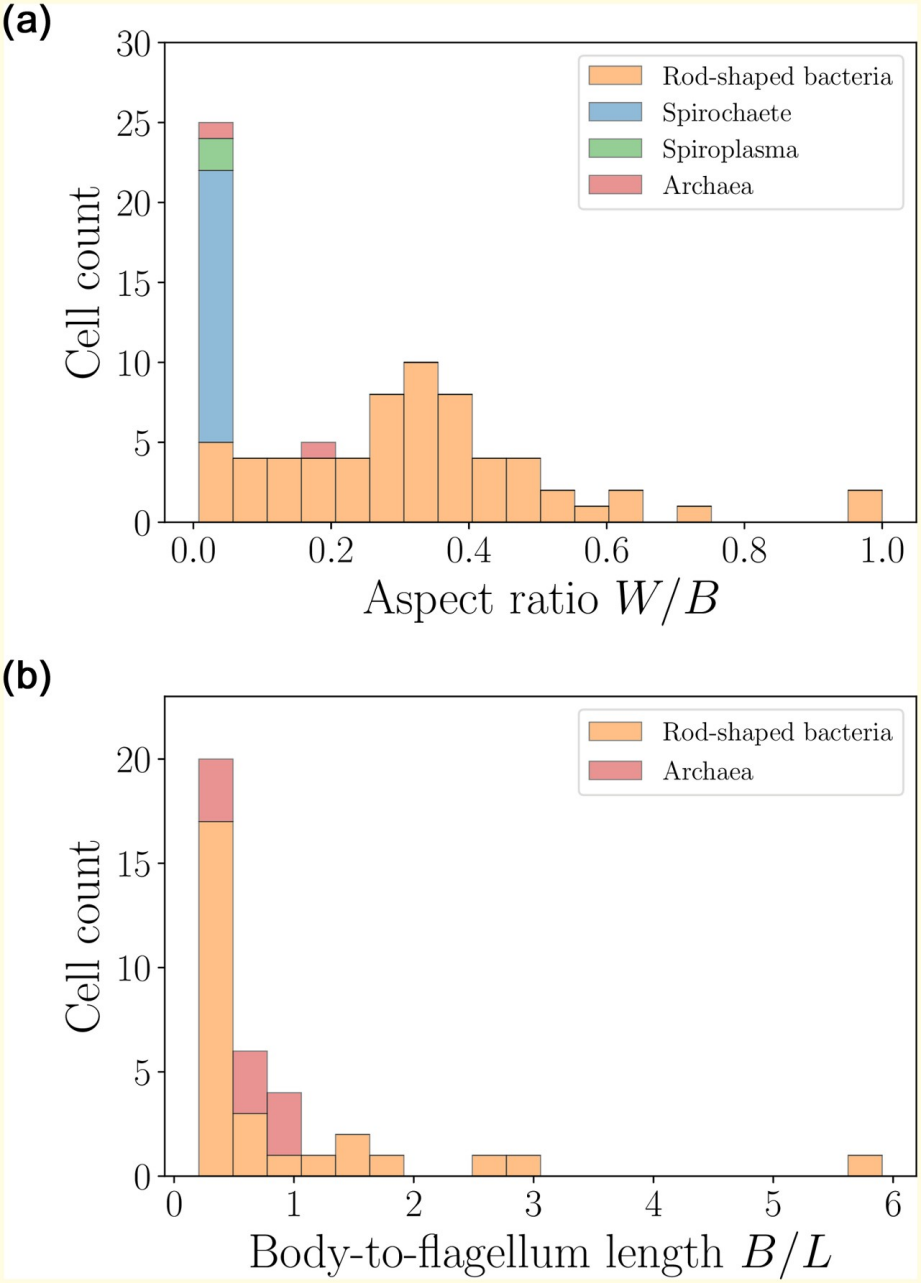
Histograms of aspect ratios *W*/*B* (left) and body-to-flagellum length *B*/*L* (right) for rod-shaped bacteria (excluding spirochaetes and *Spiroplasma*) (〈*W*/*B*〉 = 0.33 ± 0.20 (*n* = 63), 〈*B*/*L*〉 = 0.93 ± 1.19 (*n* = 28)), spirochaetes (〈*W*/*B*〉 = 0.02 ± 0.01 (*n* = 17)),*Spiroplasma* (〈*W*/*B*〉 = 0.03 ± 0.00 (*n* = 2)) and archaea (〈*W*/*B*〉 = 0.11 ± 0.06 (*n* = 2), 〈*B*/*L*〉 = 0.63 ± 0.24 (*n* = 9)). All bacteria in our study are prolate, with an average aspect ratio 〈*W*/*B*〉 = 0.25 ± 0.22 (*n* = 84), with a notable slenderness of spirochaetes and *Spiroplasma*. If the prokaryotes possess freely rotating flagella, their length often exceeds the body size 〈*B*/*L*〉 = 0.86 ± 1.05 (*n* = 37) (both spirochaetes and *Spiroplasma* are not included in the *B*/*L* graph).

The shapes of the prokaryotes are next quantified in the distributions shown in Fig 5 (left). The cells are close to ellipsoidal, with an aspect ratio *W*/*B* (body width to length) not exceeding 1 and an average of about 0.25. In contrast, spirochaetes and *Spiroplasma* are slender, with the aspect ratio not exceeding 0.05. We also plot in Fig 5 (right) the distribution of body-to-flagellum lengths for cells with external flagellar filaments (i.e. excluding spirochaetes and *Spiroplasma*). This is typically smaller than unity, indicating that the helical filaments are longer than the cell body in most cases.

**Fig 6:**
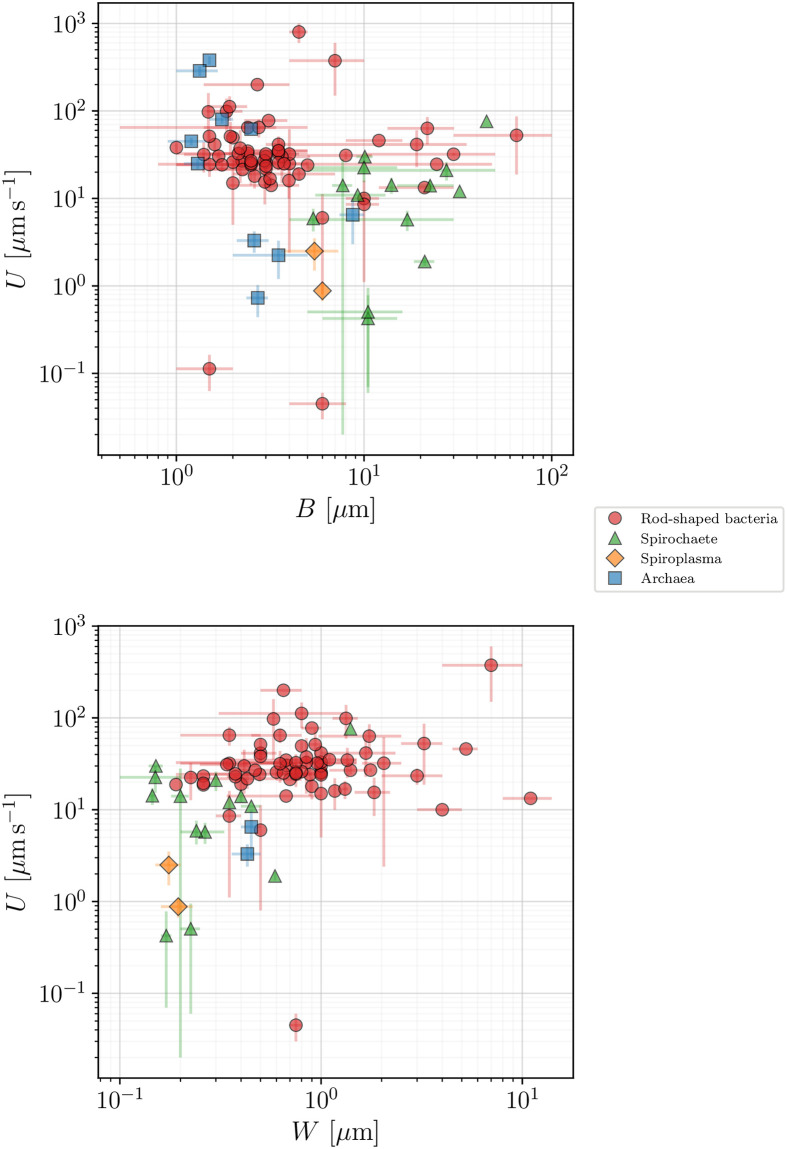
Swimming speed, *U* (*μ*m s^−1^), as function of the cell body length, *B* (*μ*m, top), and body width, *W* (*μ*m, bottom), for all our registered prokaryotes. Error bars represent standard deviations, whenever available, or the span between the recorded maximum and minimum values.

The swimming speed for all prokaryotes in our database is plotted in Fig 6 against the cell body length (top panel) and width (bottom panel), with colours used to divide our dataset into the specific taxonomic groups. Clearly, a wide spread of values exist for the swimming speeds and in the next section we use a mathematical model for bacterial locomotion in order to gain further insight into the data.

### 3.2 Modelling of swimming for flagellated prokaryotes

We focus in what follows on the case of rod-shaped prokaryotes. The flagellar locomotion of bacteria relies on the motor rotation being transmitted to the passive flagellar filament via the flexible hook [20]. The rotation of the motor is generated by ion fluxes and in the forward propulsion mode the rotary motor works at approximately constant torque [77]. The value of this torque, however, has been under some debate. Berry and Berg estimated the stall torque in an optical tweezers experiment to be of the order of 4600 pNnm [78], while Reid *et al*. attached micrometer beads to flagella to measure the motor torque to be 1260 ± 190 pNnm [79]. In magnetic tweezers experiments involving the attachment of paramagnetic beads, the corresponding torque amounted to 874 ± 206 pNnm [80]. In contrast, a simplified theoretical model predicts a lower value of 370 ± 100 pNnm [81] while recent numerical simulations reported values in the range 440 − 820 pNnm [82]. Kinosita *et al*. [76] managed to observe in detail the flagellar rotation of the archaeon *Halobacterium salinarum* and estimated its motor torque to be as low as 50 pN nm. However, different species of bacteria can have very different motor structures [83], which leads to a wide range of possible values for the propulsive torque [84].

In order to estimate the motor torque of various species in our dataset, we consider a generalised mathematical model for the swimming of flagellated prokaryotes. For simplicity we focus on the case of a cell rotating a single helical filament [85]. However, the resulting model should remain valid for a prokaryote with many flagella, since during swimming all flagellar filaments bundle together and rotate as if they formed a single helix [11]. Furthermore, as we show later, the model can be easily adapted to cope with the impact of bundled flagella.

A prokaryotic flagellar filament of length *L* is well approximated by a rigid helix of pitch *λ* and radius *h* (as shown in Fig 1, top), rotating at an angular velocity *ω* = 2*πf* relative to the cell body, where *f* is the frequency of rotation of the flagellum. In turn, the cell body rotates at an angular velocity Ω relative to the fluid to maintain an overall torque balance on the cell. At low Reynolds number, the helical filament is subject to a hydrodynamic thrust *F* and a viscous torque *T* which depend linearly with the axial speed *U* and the rotation rate of the filament relatively to the fluid Ω + *ω*. This may be written as

$$\begin{array}{c} (\begin{array}{c} F\\ T \end{array}{)}_{\text{flagellum}}=-(\begin{array}{cc} f_{11} & f_{12}\\ f_{12} & f_{22} \end{array})(\begin{array}{c} U\\ \Omega +\omega \end{array}). \end{array}$$ (1)

Prokaryotic flagellar filaments are very thin, with typical radii of 0.02 *μ*m and average lengths a thousand times greater (the mean value of all lengths in our database is 〈*L*〉 = 8.28 *μ*m), so that the coefficients of the symmetric matrix *f*_*ij*_ can be evaluated using the resistive-force theory of viscous hydrodynamics valid for very slender filaments [59]. Integrating the local hydrodynamic forces on each small segment of the flagellum using the viscous drag coefficients per unit length, *c*_⊥_ and *c*_∥_, in the directions normal and tangential to each segment respectively (see details below), yields the classical result that the resistance coefficients in Eq (1) are given by

$$f_{11}=(c_{\|}\;{\text{cos}}^{2}\;\theta +c_{⊥}\;{\text{sin}}^{2}\;\theta )\;L,$$ (2a)

$$f_{12}=(c_{⊥}-c_{\|})\;\text{sin}\;\theta \;\text{cos}\;\theta \;hL,$$ (2b)

$$f_{22}=\left(c_{⊥}\;{\text{cos}}^{2}\;\theta +c_{\|}\;{\text{sin}}^{2}\;\theta \right)\;h^{2}L.$$ (2c)

where *θ* = arctan(2*πh*/λ) is the helix pitch angle [11, 86].

The cell body, modelled as a prolate spheroid of length *B* and diameter *W* (Fig 1, top), is subject to a hydrodynamic force *F* proportional to the swimming speed *U* and a hydrodynamic torque *T* proportional to its rotation rate Ω. Assuming the cell to rotate about its principal axis leads to

$$\begin{array}{c} (\begin{array}{c} F\\ T \end{array}{)}_{\text{body}}=-(\begin{array}{cc} b_{11} & 0\\ 0 & b_{22} \end{array})(\begin{array}{c} U\\ \Omega \end{array}), \end{array}$$ (3)

where the off-diagonal coefficients vanish due to the symmetry of the body.

During steady, straight swimming, the sum of forces and torques on the swimming bacterium must vanish, and thus combining Eqs (1) and (3) we obtain a linear system of equations for the swimming speed and angular rotation as a function of the rotation rate of the filament as

$$\begin{array}{c} (\begin{array}{cc} b_{11}+f_{11} & f_{12}\\ f_{12} & b_{22}+f_{22} \end{array})(\begin{array}{c} U\\ \Omega \end{array})=-(\begin{array}{c} f_{12}\\ f_{22} \end{array})\omega . \end{array}$$ (4)

Solving for *U* and Ω as functions of *ω* leads to the relations

$$U=\frac{f_{12}b_{22}}{f_{12}^{2}-\left(b_{11}+f_{11}\right)\left(b_{22}+f_{22}\right)}\omega ,$$ (5a)

$$\Omega =\frac{f_{22}\left(f_{11}+b_{11}\right)-f_{12}^{2}}{f_{12}^{2}-\left(b_{11}+f_{11}\right)\left(b_{22}+f_{22}\right)}\omega .$$ (5b)

The torque *T*_*m*_ exerted by the flagellar motor is, by definition, given by *T*_*m*_ = *f*_12_ *U* + *f*_22_(Ω + *ω*), which after substitution into Eq (5) yields

$$\begin{array}{c} T_{m}=\frac{b_{22}\left(f_{12}^{2}-f_{22}\left(b_{11}+f_{11}\right)\right)}{f_{12}^{2}-\left(b_{11}+f_{11}\right)\left(b_{22}+f_{22}\right)}\omega , \end{array}$$ (6)

and therefore the ratio between the swimming speed and the torque exerted by the motor is only a function of the various resistance coefficients, as

$$\begin{array}{c} \frac{U}{T_{m}}=\frac{f_{12}}{f_{12}^{2}-f_{22}\left(b_{11}+f_{11}\right)}. \end{array}$$ (7)

The ratio between $f_{12}^{2}$ and *f*_11_ *f*_22_ can be computed using the expressions given by Eq (2) and we obtain

$$\begin{array}{c} \frac{f_{12}^{2}}{f_{11}f_{22}}=\frac{{\left(c_{⊥}-c_{\|}\right)}^{2}\;{\text{sin}}^{2}\;\theta \;{\text{cos}}^{2}\;\theta }{\left(c_{\|}\;{\text{cos}}^{2}\;\theta +c_{⊥}\;{\text{sin}}^{2}\;\theta \right)\left(c_{\|}\;{\text{sin}}^{2}\;\theta +c_{⊥}\;{\text{cos}}^{2}\;\theta \right)}. \end{array}$$ (8)

The right hand side of Eq (8) is always positive (since *c*_⊥_, *c*_∥_ > 0). Its derivative with respect to *θ* is given by

$$\begin{array}{c} \frac{2c_{⊥}c_{\|}{\left(c_{⊥}-c_{\|}\right)}^{2}\;\text{sin}\;\theta \;\text{cos}\;\theta \left(\;{\text{cos}}^{2}\;\theta -\;{\text{sin}}^{2}\;\theta \right)}{{\left(c_{⊥}\;{\text{sin}}^{2}\;\theta +c_{\|}\;{\text{cos}}^{2}\;\theta \right)}^{2}{\left(c_{⊥}\;{\text{cos}}^{2}\;\theta +c_{\|}\;{\text{sin}}^{2}\;\theta \right)}^{2}}, \end{array}$$ (9)

which has θ={kπ/4,k∈ℤ} as roots, for all values of *c*_∥_ and *c*_⊥_. Since *θ* = {0, *π*/2} are zeros of Eq (8) themselves, *θ* = *π*/4 gives the maximum possible value for the ratio as

$$\begin{array}{c} \frac{f_{12}^{2}}{f_{11}f_{22}}\leq \frac{{\left(c_{⊥}-c_{\|}\right)}^{2}}{{\left(c_{⊥}+c_{\|}\right)}^{2}}=(\frac{1-c_{\|}/c_{⊥}}{1+c_{\|}/c_{⊥}}{)}^{2}. \end{array}$$ (10)

It is usually a good approximation to take *c*_∥_/*c*_⊥_ ≈ 1/2, so that the ratio $f_{12}^{2}/(f_{11}f_{22})$ is bound from above by 1/9, and one may thus approximately neglect the contribution of $f_{12}^{2}$ in the denominator of Eq (7), yielding the simplified result

$$\begin{array}{c} |\frac{U}{T_{m}}|\approx \frac{f_{12}}{f_{22}\left(b_{11}+f_{11}\right)}. \end{array}$$ (11)

The drag coefficient *b*_11_ for a prolate spheroid of length *B* and diameter *W* depends on a geometric factor *C*_*FB*_ that involves the eccentricity *e* of the spheroid, given by $e=\sqrt{1-(W/B{)}^{2}}$ (0 ≤ *e* < 1), as [87]

$$\begin{array}{c} b_{11}=3\pi \eta BC_{FB}\left(W/B\right),\;C_{FB}=\frac{8}{3}e^{3}[-2e+\left(1+e^{2}\right)\;\text{log}\;\frac{1+e}{1-e}{]}^{-1}. \end{array}$$ (12)

The asymptotic limit of very slender spheroids, evaluated in Ref. [87], also gives the friction coefficients for the motion of a rod of length *L* and maximal thickness 2*b* as

$$\begin{array}{c} c_{⊥}=\frac{4\pi \eta }{\text{log}(L/b)+1/2},\;\;\;c_{\|}=\frac{2\pi \eta }{\text{log}(L/b)-1/2},\;\;\;\left(b/L\ll 1\right), \end{array}$$ (13)

which, for large aspect ratios, yield the approximation *c*_∥_/*c*_⊥_ ≈ 1/2, as above. Assuming for simplicity the pitch angle to be *θ* ≈ *π*/4, and using the friction coefficients as in Eq (13), Eq (11) takes the final explicit form

$$\begin{array}{c} U=\frac{T_{m}}{\eta \xi^{2}},\;\xi =\sqrt{9\pi h(BC_{FB}(W/B)+[L/(\text{log}(L/b)+1/2)])}, \end{array}$$ (14)

where the characteristic length *ξ* depends solely on the morphology of the swimmer and results from the interplay of body and flagellum size. The result in Eq (14) relates therefore the swimming speed *U* to the flagellar motor torque *T*_*m*_ via the viscosity of the fluid (*η*) and a morphological factor (*ξ*). Note that by adjusting the helix thickness 2*b*, the model can address the impact of having several filaments inside the flagellar bundle [88]. Since the effect of *b* in Eq (14) is logarithmic, its impact on our results is minimal.

### 3.3 Insights from data

**Fig 7:**
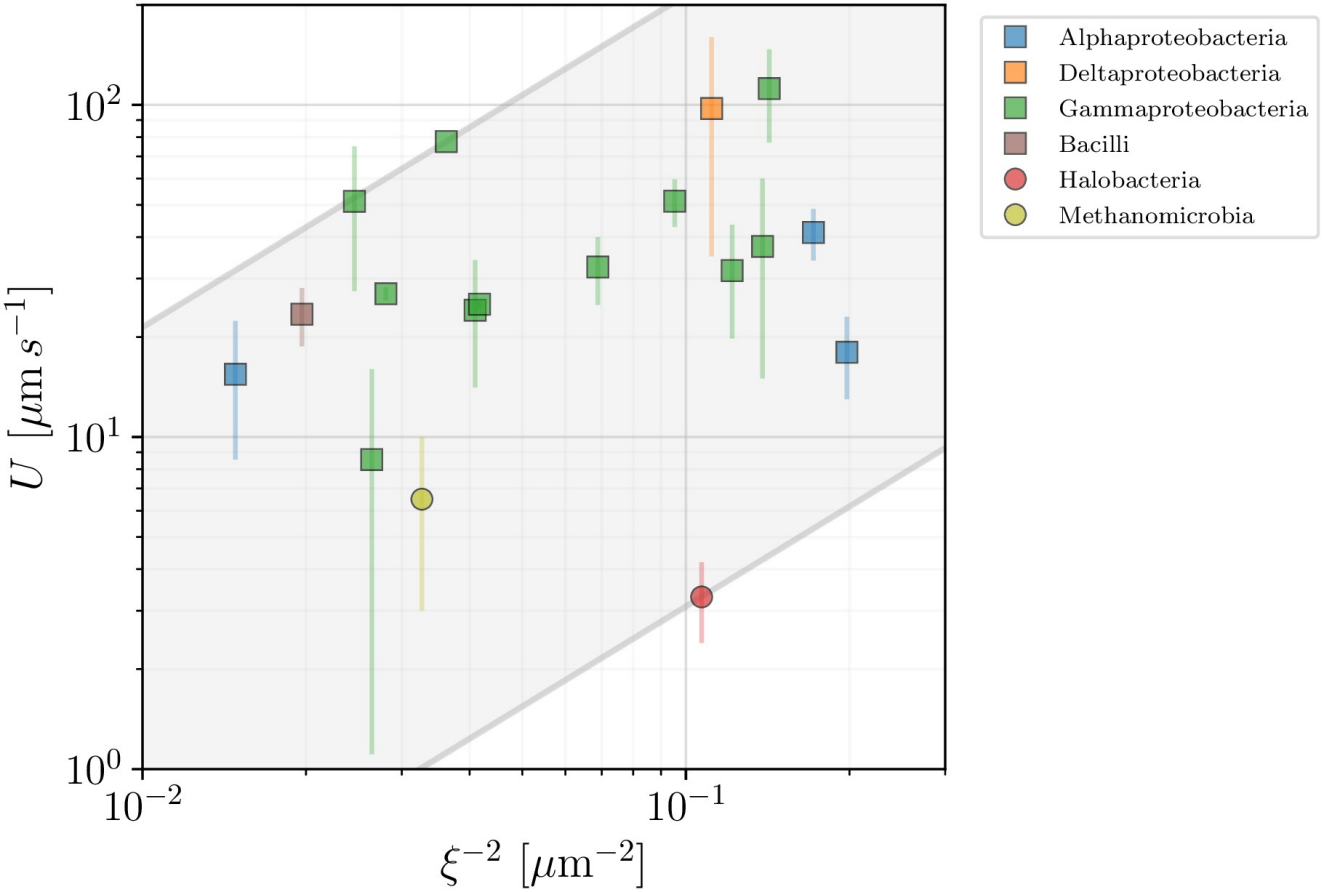
Propulsion speed of rod-shaped prokaryotes vs morphological factor 1/*ξ*^2^. Bacteria are plotted in squares and archaea in circles with colours used to distinguish between the different taxonomic classes. The plot, along with Eq (14), allows to estimate the range of bacterial motor torques 27.48 − 1907 pN nm, represented by the shaded area.

We can now use the model introduced above in order to help organise our database and provide a simple estimate of the range of motor torques in the available data. In Fig 7 we plot the swimming speed, *U*, for rod-shaped bacteria and archaea as a function of the morphological factor 1/*ξ*^2^ for all the species for which our database gives access to the parameters involved in the definition of *ξ* in Eq (14) (we assumed the thickness of the flagella to be 2*b* = 0.02 *μ*m in all cases). The ratio between *U* and 1/*ξ*^2^ should yield an estimate of the effective flagellar motor torque, *T*_*m*_. An important limitation is that the value of the viscosity is, alas, rarely given directly in the studies gathered in our database. We thus assume the viscosity *η* in Eq (14) to be that of water at 25°C and in Fig 7 we display the range of motor torques so obtained using parallel lines enclosing the shaded area. The lower and upper bounds of the motor torque *T*_*m*_ are obtained to be 27.48 pN nm (for *Halobacterium salinarum*) and 1907 pN nm (*Pseudomonas fluorescens*). This large range highlights the intrinsic variability within this group, corresponding to the observed scatter of the data.

## 4 Flagellated eukaryotes (excluding spermatozoa and ciliates)

Eukaryotic cells are not just morphologically distinct from prokaryotes, they also have different important biological features, including the presence of a cellular nucleus. Their propulsion is enabled by an internal mechanism that is fundamentally different from, and more complex than, that of prokaryotes. The central structure of eukaryotic flagella and cilia is termed the axoneme and is usually composed of nine microtubule doublet filaments surrounding a tenth central pair of microtubules. Cross-linking dynein motors allow the relative sliding of the microtubules, which results in the propagation of bending deformations along the flexible flagellum [89] that can take the form of travelling waves, either planar or helical, as well as of complex two- (2D) and three-dimensional (3D) kinematics.

The eukaryotic flagellar waves usually propagate from the flagellum base to its distal end, but some species have waves travelling in the other direction. Similarly, while most species swim with flagella trailing, some, such as the alga *Ochromonas danica*, self-propel with their flagella leading the cell. We refer to Jahn & Votta for an extensive overview of the observed beating patterns [90]. One of the most fundamental beating patterns displayed by eukaryotic cells is a simple planar sine wave, and we will use it as the basis for the modelling introduced below. Note that flagella of some eukaryotic species display perpendicularly attached rigid structures, termed mastigonemes, which give a hairy microstructure to the flagellum and allow the cells to generate propulsion in the same direction as the propagating wave [19, 91]. Some algae such as *Chlamydomonas* do not even rely on waves to swim, but do so by swinging a pair of short flagella in a breaststroke way.

Eukaryotic cells are generally one or two orders of magnitude larger in size than prokaryotes and are therefore more easily observed experimentally. A number of past review papers gathered swimming speeds and body lengths for tens of organisms [92–95]. Our database builds on, and extends, these datasets by introducing a number of new important cellular parameters and new organisms. Note that parts of our data for eukaryotic cells, particularly the average sizes and swimming speeds have been published elsewhere [39].

Among swimming unicellular eukaryotes, three families with different morphology can be distinguished: flagellates, spermatozoa, and ciliates. Flagellates—the focus of this section—typically possess a few long flagella attached to their bodies, which they actuate in order to achieve propulsion (for organisms in this section, the typical number of flagella rarely exceeds 10). Spermatozoa are also remarkable flagellated swimmers but they lack the ability to reproduce, thus are not considered living organisms. Lastly, ciliates are much larger in size and are covered by dense arrays of cilia, which are short flagella that move collectively to create flow along the cell surfaces. The qualitative difference in their swimming speeds, as well as their geometric characteristics such as their size and their number of flagella, warrants separate statistical analysis for each group [39]; spermatozoa are therefore addressed in Sec. 5 and ciliates in Sec. 6.

### 4.1 Geometry and swimming speeds of the cells

**Fig 8:**
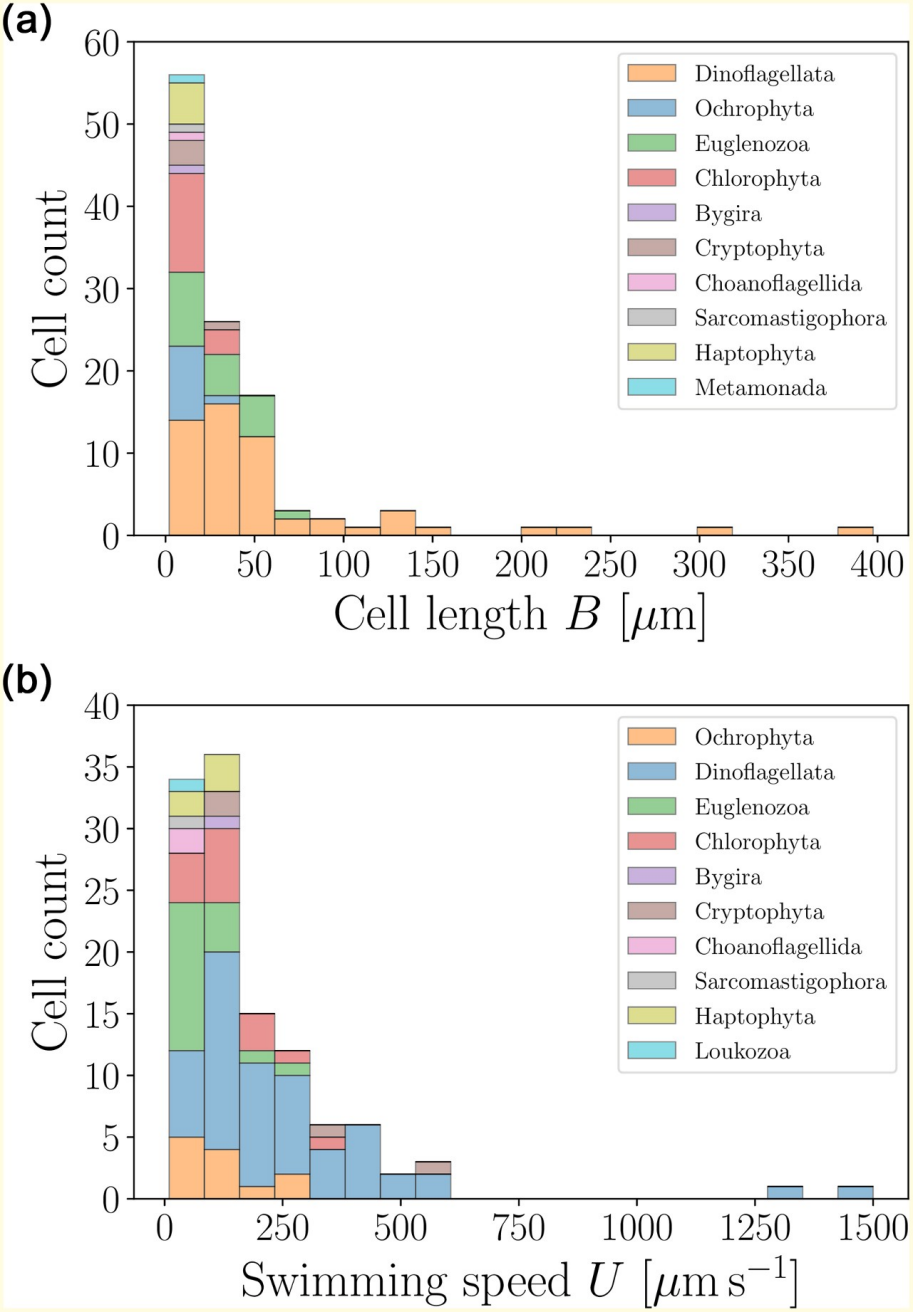
Histograms of body lengths, *B* (*μ*m, left), and swimming speeds, *U* (*μ*m s^−1^, right), for flagellated eukaryotic swimmers (excluding spermatozoa and ciliates) in our dataset. The average cell length is 〈*B*〉 = 38.87±56.64 *μ*m (*n* = 113) and the average swimming speed 〈*U*〉 = 186.70 ± 208.77 *μ*m s^−1^ (*n* = 116).

The typical sizes and swimming speeds of eukaryotic flagellates are presented in Fig 8, based on the data from Table 7. Significantly larger and faster than prokaryotic cells, the distributions are dominated by the low-values tails.

**Fig 9:**
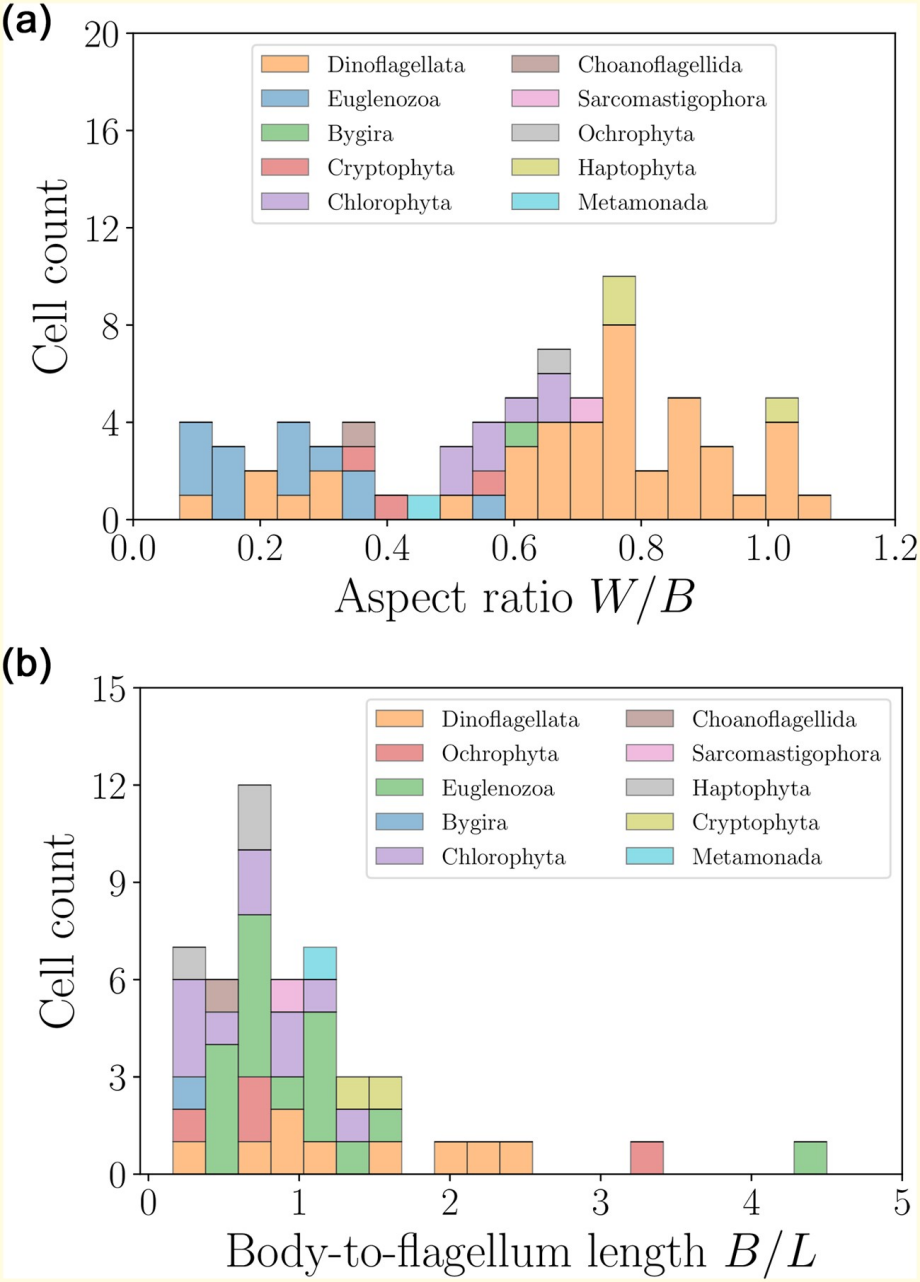
Histograms of aspect ratios *W*/*B* (left) and body-to-flagellum length ratios *B*/*L* (right) for flagellated eukaryotic swimmers. For all organisms in this category, the aspect ratios do not exceed ≈ 1.1, and the shape distribution indicates a slightly prolate shape on average, with 〈*W*/*B*〉 = 0.60 ± 0.27 (*n* = 73). The distribution of body-to-flagellum length ratios shows that flagella tend to be of length comparable to the cell body, with a few exceptions 〈*B*/*L*〉 = 1.03 ± 0.79 (*n* = 49).

Most cells are close to the average values, with several outliers in the large size and speed ranges. The statistical properties of these distributions have been discussed in detail in our previous work [39]. We may gain further insight by considering the distribution of aspect ratios for the cell bodies, *W*/*B*, and the relative cell body-to-flagella lengths, *B*/*L*, both of which are shown in Fig 9. The wide distribution of aspect ratios confirms that most flagellates are slightly prolate, although several more elongated swimmers are also reported. In addition, for most cells the ratio of body to flagella length does not exceed 1, confirming that the length of the flagella is comparable to the cell size. This feature allows to distinguish flagellated eukaryotes from spermatozoa and ciliates.

**Fig 10:**
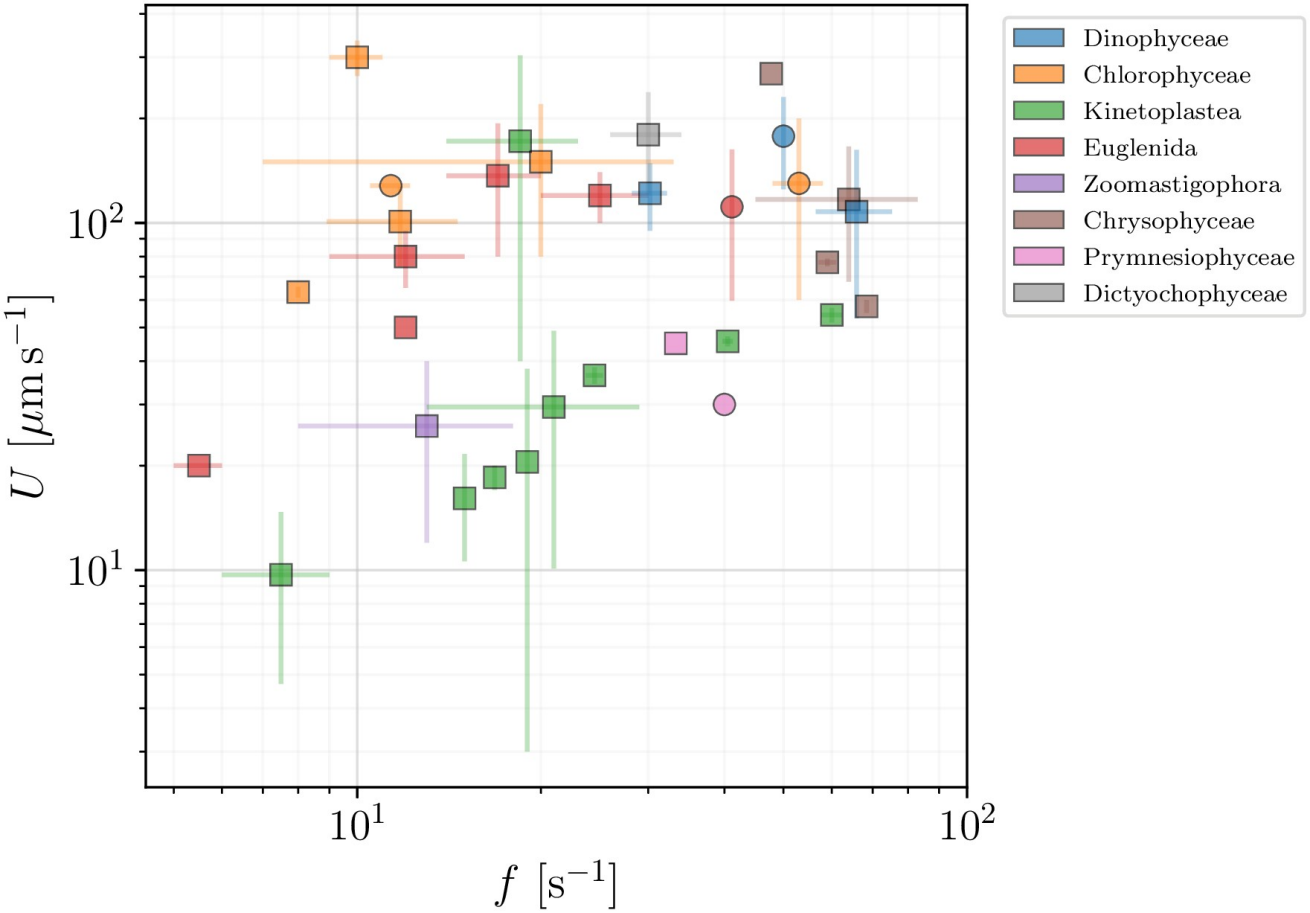
Swimming speed, *U* (*μ*m s^−1^), plotted versus the frequency of flagellar beat, *f* (s^−1^), for flagellated eukaryotes in our dataset (excluding spermatozoa and ciliates). Colours mark different classes and sub-classes. Wave-producing organisms are plotted in squares and the remaining flagellated eukaryotes are plotted in circles.

**Fig 11:**
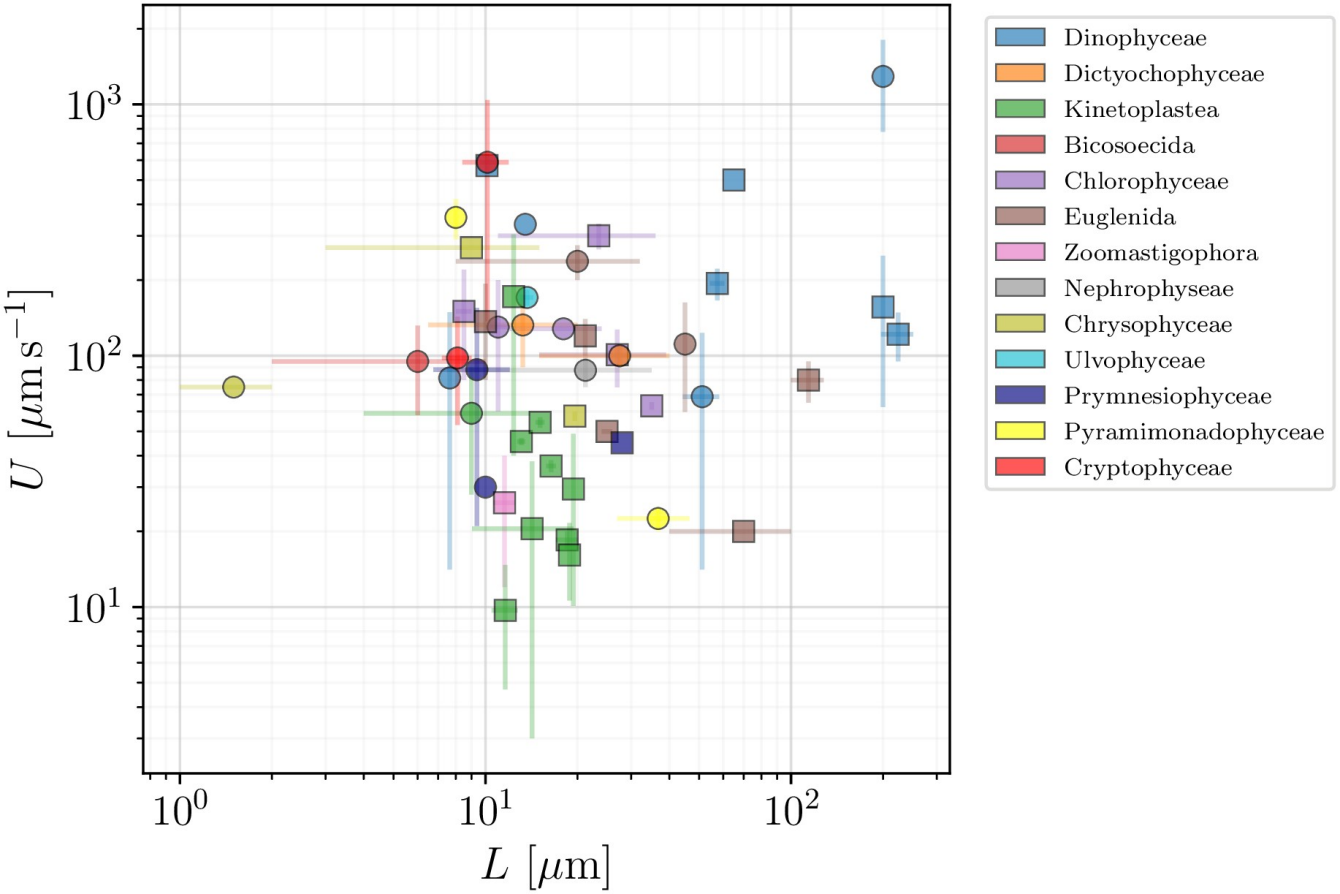
Swimming speed, *U* (*μ*m s^−1^), vs length of flagella, *L* (*μ*m), for flagellated eukaryotes in our database (excluding spermatozoa and ciliates). Taxonomic classes are marked by colours. Wave-producers are again plotted in squares, while other flagellates are plotted in circles.

In Figs 10 and 11 we next show how the swimming speeds *U* of the flagellated eukaryotes in our database vary with the flagellar beat frequencies *f* and flagellar lengths *L*, respectively. Both plots show large variations and no clear trend is evident. In the next section we will then adapt the classical derivation by Gray & Hancock [59] as a minimal model for the propulsion of eukaryotic flagellates to see the role played by these (and other) parameters in eukaryotic propulsion.

### 4.2 Modelling of swimming for flagellated eukaryotes

We base the description of the locomotion of flagellated eukaryotes on the assumption that swimming results from planar travelling waves induced in one or more flagella, which push a spheroidal cell body forward.

**Fig 12:**
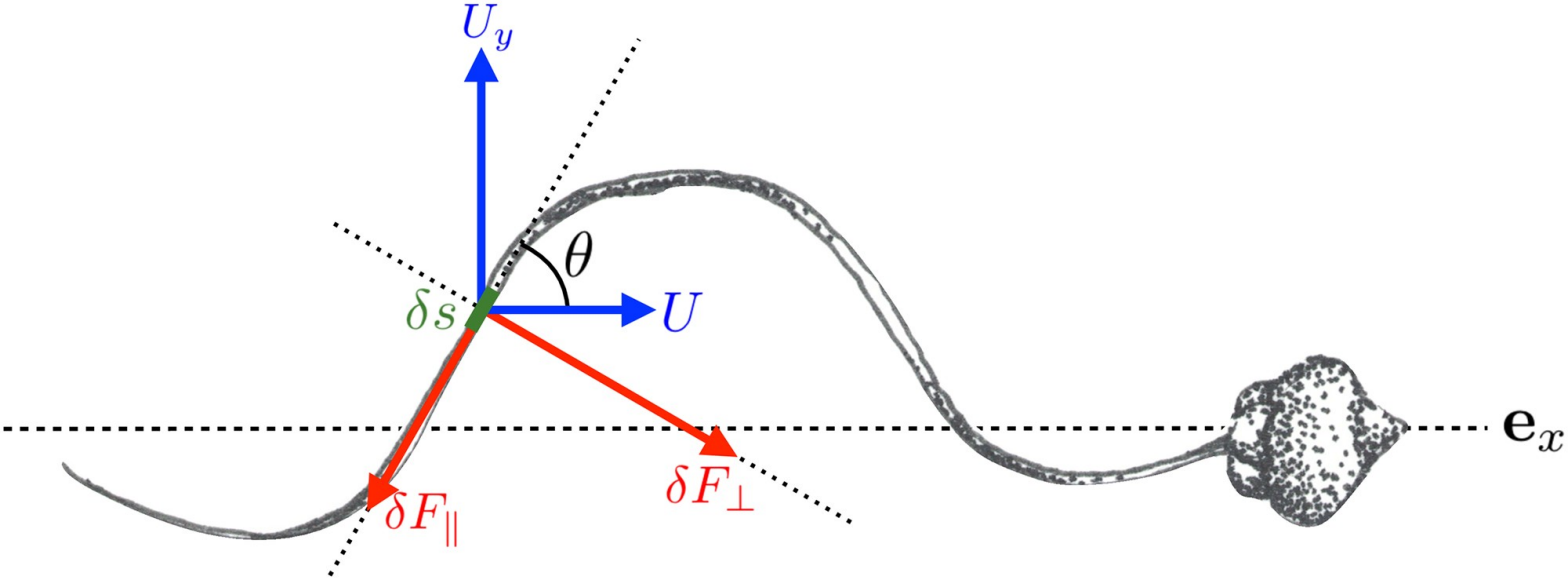
Sketch of a swimming eukaryote (spermatozoon of *Chaetopterus*, Annelida) propelled by a single flagellum. We distinguish a section of length *δs* inclined at an angle *θ* to the direction of motion **e**_*x*_, which we use to determine the local hydrodynamic forces exerting on the flagellum. Drawing by Marcos F. Velho Rodrigues.

The shape of the wave is described in Cartesian coordinates by *y* = *y*(*x*, *t*), where *x* is the direction of cell movement (see Fig 12). An infinitesimal segment of the flagellum of length *δs* inclined at an angle *θ* to the axis of movement **e**_*x*_ is then subjected to a hydrodynamic force perpendicular to its orientation, and given by

$$\begin{array}{c} \delta F_{⊥}=c_{⊥}\left(U_{y}\;\text{cos}\;\theta -U\;\text{sin}\;\theta \right)\;\delta s, \end{array}$$ (15)

and to a force tangential to the segment given by

$$\begin{array}{c} \delta F_{\|}=c_{\|}\left(U_{y}\;\text{sin}\;\theta +U\;\text{cos}\;\theta \right)\;\delta s. \end{array}$$ (16)

Here *U* and *U*_*y*_(*x*, *t*) are the local velocities of the flagellum relative to the fluid in the directions along and perpendicular to the overall direction of cell motion, respectively. Furthermore, similarly to the section on prokaryotes, *c*_⊥_ and *c*_∥_ are the drag coefficients per unit length in the directions normal and tangential to *δs*, respectively (see Eq 13).

These two force components produce an infinitesimal net thrust along the *x* direction, *δF* = *δF*_⊥_ sin *θ* − *δF*_∥_ cos *θ*, which we rewrite as

$$\begin{array}{c} \delta F=\frac{\left(c_{⊥}-c_{\|}\right)U_{y}\;\text{tan}\;\theta -U\left(c_{\|}+c_{⊥}\;{\text{tan}}^{2}\;\theta \right)}{1+\;{\text{tan}}^{2}\;\theta }\;\delta s. \end{array}$$ (17)

Taking into account the normal speed to be *U*_*y*_ = ∂*y*/∂*t*, using tan *θ* = ∂*y*/∂*x* and *δs*^2^ = *δy*^2^ + *δx*^2^, we transform Eq (17) into

$$\begin{array}{c} \delta F=\frac{\left(c_{⊥}-c_{\|}\right)\frac{\partial y}{\partial t}\frac{\partial y}{\partial x}-U(c_{\|}+c_{⊥}(\frac{\partial y}{\partial x}{)}^{2})}{\sqrt{1+(\frac{\partial y}{\partial x}{)}^{2}}}\;\delta x. \end{array}$$ (18)

We now need to specify a particular wave form of the beating pattern. One that is often observed in eukaryotic swimmers is a planar travelling wave [90] which we approximate by a single sine wave of fixed amplitude *h*, wavelength λ and beat frequency *f*

$$\begin{array}{c} y\left(x,t\right)=h\;\text{sin}\;(\frac{2\pi }{\lambda }(x+ct)), \end{array}$$ (19)

where *c* = λ*f* is the speed of the propagating flagellar waves. Substituting the sine wave into Eq (18), and taking the slender limit *c*_⊥_ ≈ 2*c*_∥_, yields

$$\begin{array}{c} \delta F=c_{\|}\frac{cA^{2}-U\left(1+2A^{2}\right)}{\sqrt{1+A^{2}}}\;\delta x, \end{array}$$ (20)

where *A* = ∂*y*/∂*x* = (2*πh*/λ) cos (2*π*(*x* + *ct*)/λ). It is convenient to introduce the number of complete waves *n*_*w*_ in the flagellum of length *L*, defined as

$$\begin{array}{cc} \frac{1}{n_{w}} & =\frac{1}{L}\int_{x=0}^{\lambda }\delta s=\frac{1}{L}\int_{0}^{\lambda }\sqrt{1+A^{2}}\;dx. \end{array}$$ (21a)

Because the integrand $\sqrt{1+A^{2}}$ is a function of period λ, a simple substitution shows that the number of waves is constant in time, and is given by

$$\frac{1}{n_{w}}=\frac{\lambda }{L}\;\Lambda (\frac{2\pi h}{\lambda }),$$ (21b)

where the auxiliary integral Λ is

$$\begin{array}{c} \Lambda \left(a\right)=\frac{1}{2\pi }\int_{0}^{2\pi }\sqrt{1+a^{2}\;{\text{cos}}^{2}\;\alpha }\;d\alpha . \end{array}$$ (22)

With the net thrust *δF* in Eq (20) being also of period λ, a good approximation of the total thrust produced by the entire flagellum independent of time is given by

$$\begin{array}{c} n_{w}\int_{x=0}^{\lambda }\delta F=n_{w}c_{\|}\lambda (cI_{1}(\frac{2\pi h}{\lambda })-UI_{2}(\frac{2\pi h}{\lambda })), \end{array}$$ (23)

where we have introduced the two auxiliary integrals

$$\begin{array}{c} I_{1}\left(a\right)=\frac{1}{2\pi }\int_{0}^{2\pi }\frac{a^{2}\;{\text{cos}}^{2}\;\alpha }{\sqrt{1+a^{2}\;{\text{cos}}^{2}\;\alpha }}\;d\alpha ,\;I_{2}\left(a\right)=\frac{1}{2\pi }\int_{0}^{2\pi }\frac{1+2a^{2}\;{\text{cos}}^{2}\;\alpha }{\sqrt{1+a^{2}\;{\text{cos}}^{2}\;\alpha }}\;d\alpha . \end{array}$$ (24)

The three functions Λ, *I*_1_ and *I*_2_ are easy to evaluate numerically. Alternatively, by writing cos^2^ *α* = (1 + cos 2*α*)/2, and neglecting the contributions of the terms in cos 2*α* in the expressions of Eqs (22) and (24), one gets explicit approximations

$$\begin{array}{c} \Lambda \left(a\right)\approx \sqrt{1+\frac{a^{2}}{2}},\;I_{1}\left(a\right)\approx \frac{a^{2}}{2\sqrt{1+\frac{a^{2}}{2}}},\;I_{2}\left(a\right)\approx \frac{1+a^{2}}{\sqrt{1+\frac{a^{2}}{2}}}. \end{array}$$ (25)

Numerical evaluation of the exact expressions for Λ, *I*_1_ and *I*_2_ shows that the approximations above hold to within 13% accuracy for all values *h*/λ < 1.

For the sake of simplicity, we shall suppose that an organism with *N* beating flagella is subject to a total thrust equal to *N* times the thrust generated by each flagellum and given by Eq (23). We therefore neglect hydrodynamic interactions between the flagella, which we assume all beat collinearly along the swimming direction.

Steady swimming requires the thrust produced by the flagella to be balanced by the drag acting on the cell body. The latter is modelled as a prolate spheroid of length *B* and diameter *W*. The balance of forces acting on the microorganism along *x* is then

$$\begin{array}{c} Nn_{w}\int_{x=0}^{\lambda }\delta F-3\pi \eta UBC_{FB}=0, \end{array}$$ (26)

with *C*_*FB*_(*W*/*B*) given by Eq (12). The swimming speed *U* can thus be written as

$$\begin{array}{c} \frac{U}{\lambda f}=\frac{I_{1}(\frac{2\pi h}{\lambda })}{I_{2}(\frac{2\pi h}{\lambda })+\frac{3\pi \eta BC_{FB}\left(W/B\right)}{Nn_{w}c_{\|}\lambda }}, \end{array}$$ (27)

or using the definition of *n*_*w*_ in Eq (21) as

$$\begin{array}{c} \frac{U}{\lambda f}=\frac{I_{1}(\frac{2\pi h}{\lambda })}{I_{2}(\frac{2\pi h}{\lambda })+\frac{3\pi \eta }{Nc_{\|}}\frac{B}{L}C_{FB}\left(W/B\right)\Lambda (\frac{2\pi h}{\lambda })}. \end{array}$$ (28)

By using *c*_∥_ = 2*πη*[log(*L*/*b*) − 1/2]^−1^ as in Eq (13), and approximating integrals *I*_1_, *I*_2_ and Λ with the expressions in Eq (25), we arrive at the final expression

$$\begin{array}{c} U=\frac{2\pi^{2}h^{2}f}{\lambda }[1+\frac{4\pi^{2}h^{2}}{\lambda^{2}}+\frac{3B}{2NL}C_{FB}\left(W/B\right)(1+\frac{2\pi^{2}h^{2}}{\lambda^{2}})(\text{log}\;\frac{L}{b}-\frac{1}{2}){]}^{-1}. \end{array}$$ (29)

### 4.3 Insights from data

**Fig 13:**
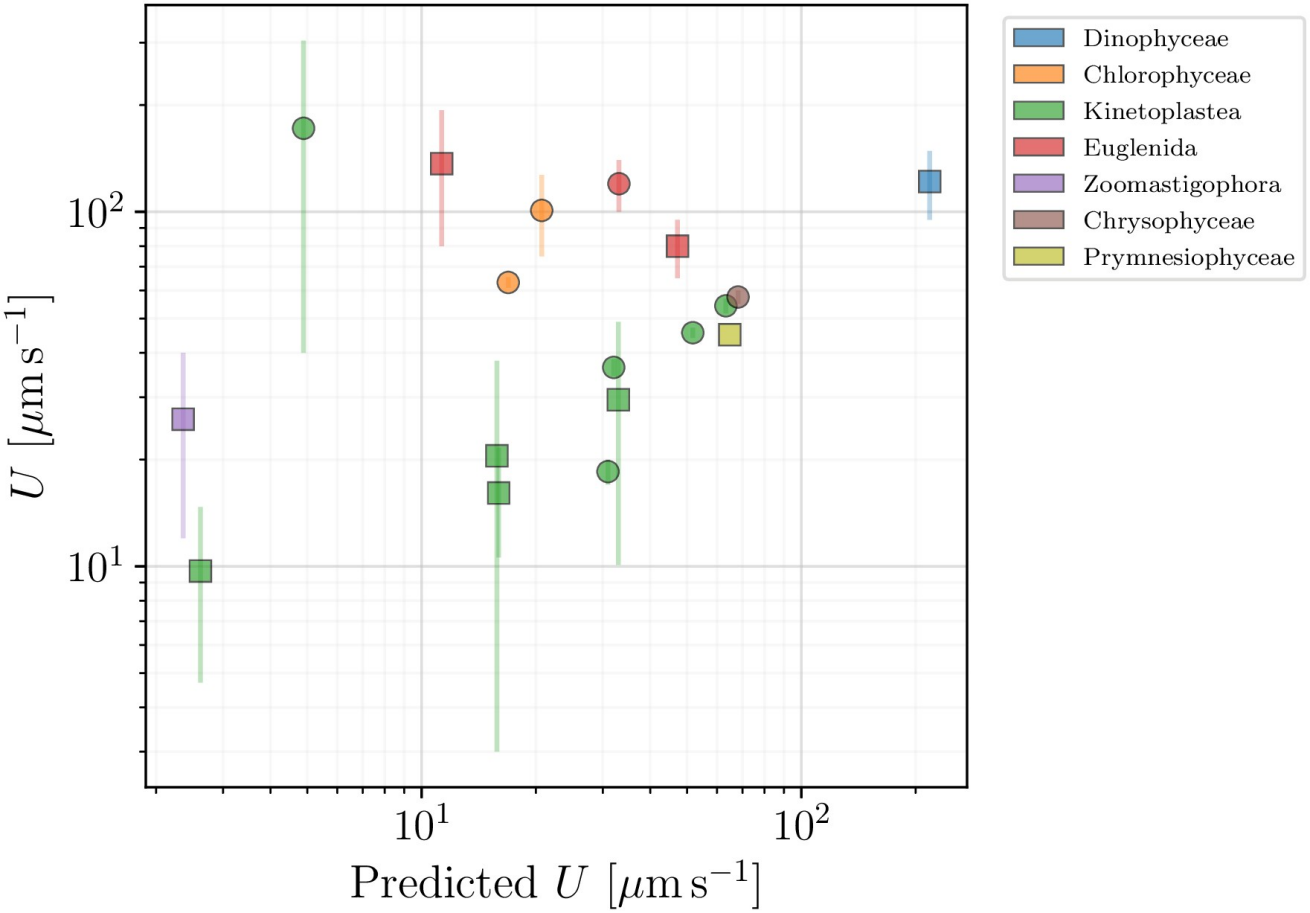
Swimming speeds of flagellated eukaryotes (excluding spermatozoa and ciliates) reported in the database plotted against the theoretical prediction of Eq (28). Colours mark different classes. Square symbols mark organisms for which the prediction was directly calculated from the available data, while circles represent organisms for which either the body width or one of the flagellar characteristics has been estimated (see text for details).

We can now use our model to help organise our data on flagellated eukaryotes. In Fig 13, we compare the swimming speeds from our dataset with those predicted by the theoretical model in Eq (28). Square symbols mark organisms for which all the quantities needed to calculate the predicted speed were available. The species plotted in circles in the figure had their data incomplete. Whenever the body width *W* was unavailable, we estimated its value using the average aspect ratio 〈*W*/*B*〉 = 0.60 of Fig 9. When one parameter of the flagellar wave was missing, we estimated it with the help of Eq (21). The radii of the flagella were all fixed at *b* = 0.2 *μ*m.

In Fig 13, we see a cluster of data points (mostly the class Kinetoplastea) that correlate well with the expected linear dependence. However, many of the organisms have a swimming speed that significantly exceeds the predicted values. This may point to other mechanisms being involved, such as different beating patterns and cell body shapes, which would require a more careful examination. Nevertheless, the basic framework proposed by the model provides a useful estimate of the lower bound for the swimming speed, which can be exceeded by adopting more effective locomotion strategies suited to the organism and its environment.

## 5 Spermatozoa

The motile behaviour of the spermatozoa of animals has been studied in detail since the beginnings of microscopy due to its importance for reproductive health. Because a correlation between motility and fertility has been shown to exist [96, 97], numerous species of fish [98], birds [99], mammals [41, 100, 101], insects [102–105] and sea urchins [106] have had their spermatozoa examined. A particular focus is often placed on the relation between either the swimming speed or the amplitude of lateral displacement of the cell body and the success in fertilisation by human spermatozoa [7].

A remarkable geometrical characteristic of spermatozoa, at least in comparison with other flagellated eukaryotes, is their relatively small heads compared to the length of their flagella. Despite this difference, the flagella of spermatozoa have the same structure detailed above for other eukaryotic cells, and are likewise capable of creating complex waveforms. The mathematical modelling of flagellar locomotion outlined in the previous section is thus also applicable to the case of swimming spermatozoa.

Our database of swimming spermatozoa contains 60 different species, for which various geometric and dynamic data were found. These include sperm cells of the taxonomic classes Insecta, Actinopterygii, Mammalia, Amphibia, Polychaeta, Ascidiacea, Echinoidea, Aves, and Bivalvia. As mentionned above, databases of morphological measurements for over 400 spermatozoa, particularly of mammalian species, are available in literature [62–64] but since they do not include motility data they are not included in our database.

### 5.1 Geometry and swimming speeds of the cells

**Fig 14:**
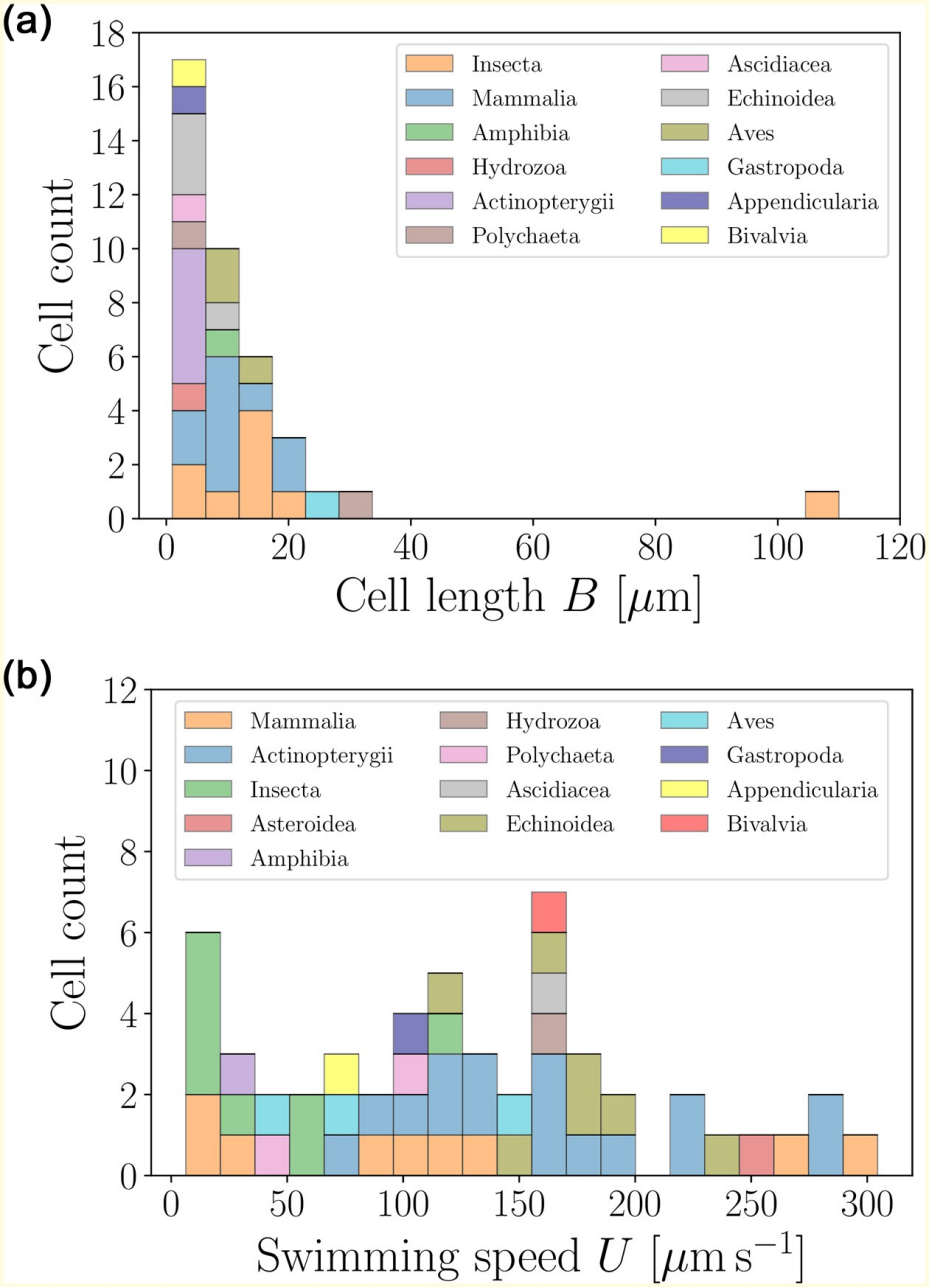
Histograms of body lengths, *B* (*μ*m, left), and swimming speeds, *U* (*μ*m s^−1^, right), for the spermatozoa in the database. The average cell length is 〈*B*〉 = 12.21 ± 17.25 *μ*m (*n* = 39), while the the average swimming speed is 〈*U*〉 = 127.23 ± 78.49 *μ*ms^−1 (*n* = 52)^ over a wide distribution. We use colours to distinguish between the different taxonomic classes.

The distribution of cell body sizes and swimming speeds of spermatozoa are shown in Fig 14, based on the data from Table 8. With body sizes hardly exceeding 30 *μ*m (except for one outlier, the cricket spermatozoon, with a size of over 100 *μ*m), we see that spermatozoa are typically small compared to other eukaryotic cells. The distribution of swimming speeds is relatively uniform, reaching up to 300 *μ*m s^−1^. While their average speeds are close to those of flagellated eukaryotes from Sec. 4, the distribution of speeds is dramatically different, deviating from the log-normal seen for other flagellated eukaryotes [39].

**Fig 15:**
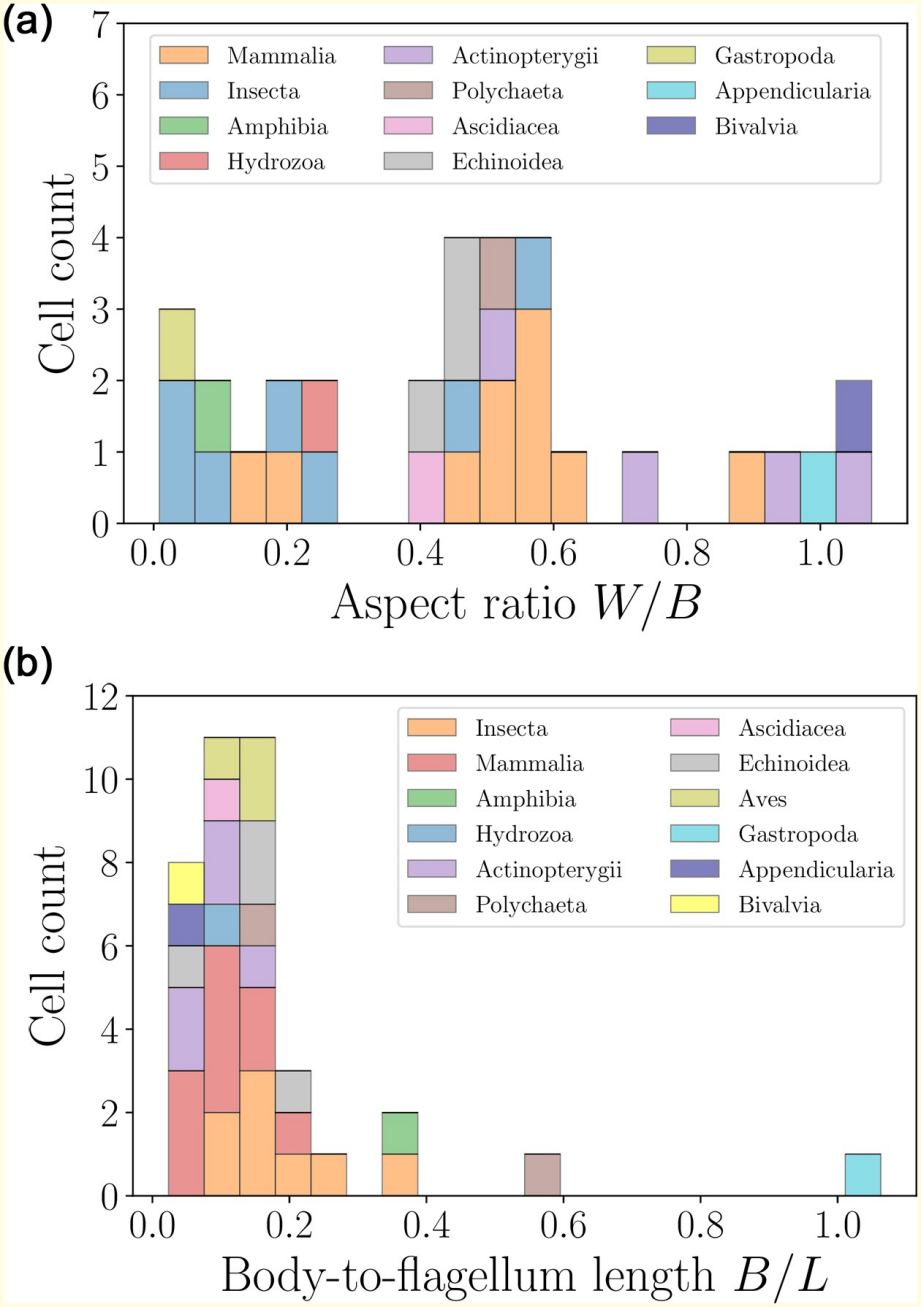
Histograms of aspect ratios *W*/*B* (left) and body-to-flagellum length *B*/*L* (right) for spermatozoa (colours mark the different taxonomic classes). The distribution of cell aspect ratios is rather wide, and yields an average value of 〈*W*/*B*〉 = 0.47±0.30 (*n* = 31). The size-to-flagellum length ratios are mostly close to the average 〈*B*/*L*〉 = 0.17 ± 0.18 (*n* = 38), showing that in spermatozoa the flagellum length is typically much larger than the cell body.

A further inspection of the geometry reveals that the distribution of sperm cell aspect ratios (Fig 15, left) is widely spread, ranging from elongated to spherical. A clear distinguishing feature for spermatozoa is the body-to-flagella length ratio (Fig 15, right), which is peaked at small values, showing that the spermatozoa of most species have flagella that are over fivefold longer than their body sizes.

### 5.2 Hydrodynamic model for locomotion

The locomotion of flagellated spermatozoa follow the same hydrodynamic principles as discussed in detail in Sec. 4. We may thus use as our starting point the the result in Eq (28), which upon using the drag coefficient *c*_∥_ = 2*πη*[log(*L*/*b*) − 1/2]^−1^ and *N* = 1 takes the form

$$\begin{array}{c} \frac{U}{\lambda f}=\frac{I_{1}\left(2\pi h/\lambda \right)}{I_{2}\left(2\pi h/\lambda \right)+\frac{3B}{2L}C_{FB}\left(W/B\right)\Lambda \left(2\pi h/\lambda \right)\left[\text{log}\left(L/b\right)-1/2\right]}. \end{array}$$ (30)

Note that the second term in the denominator of the right-hand side of Eq (30) is the hydrodynamic load of the dragging cell body, which we include although the flagella are notably longer than cell bodies for spermatozoa.

### 5.3 Insights from data

**Fig 16:**
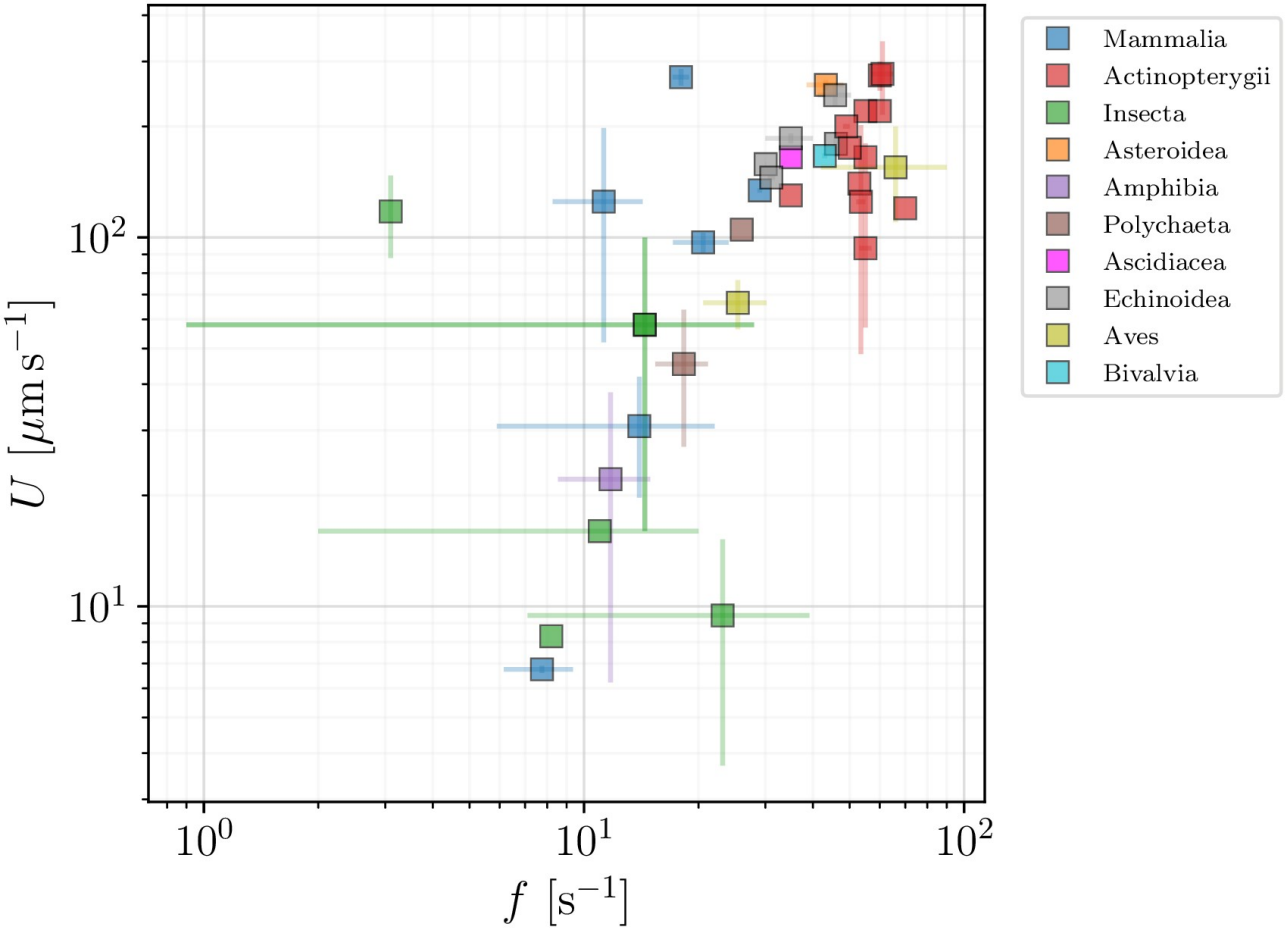
Swimming speeds, *U* (*μ*m s^−1^), as function of flagellar beat frequency *f* (s^−1^), for spermatozoa. A strong correlation between *U* and *f* is apparent on the figure.

**Fig 17:**
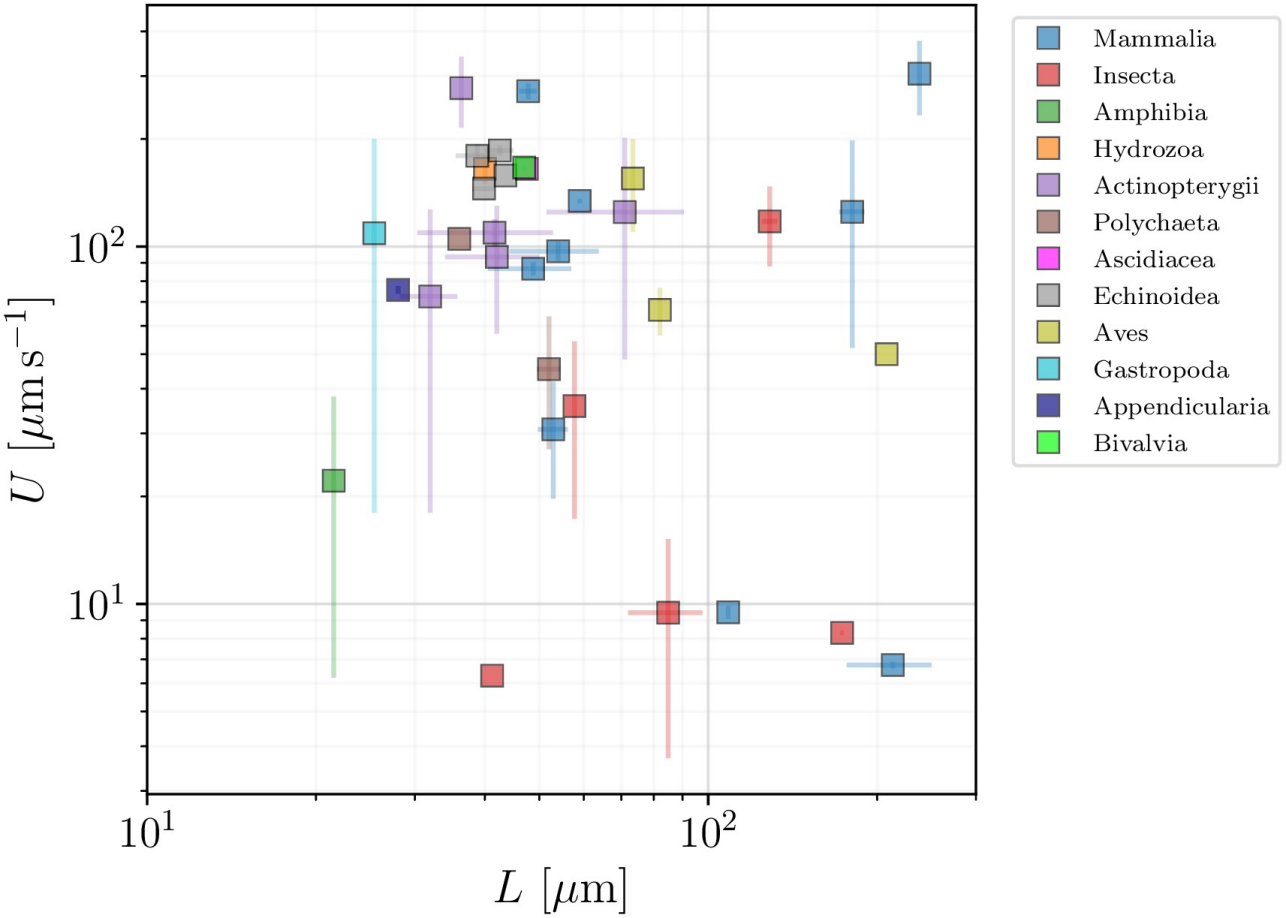
Swimming speeds, *U* (*μ*m s^−1^), as function of flagellar lengths, *L* (*μ*m), for spermatozoa. In contrast with the result in Fig 16, no clear correlation between *U* and *L* is observed here.

We again turn our attention to the behaviour of the swimming speeds for the cells. In Fig 16, we examine the dependence of the spermatozoa swimming speed *U* on the flagellar beat frequency, *f*. With most spermatozoa operating in the frequency range between 10 and 100 Hz, and swimming speeds of up to 300 *μ*m s^−1^, we observe a pronounced correlation between these two variables across our database. In Fig 17, we also show the dependence of the swimming speed *U* on the flagellar length *L*, which ranges from about 20 to 120 *μ*m. Here, in contrast, no direct or apparent correlation is seen between *U* and *L*.

**Fig 18:**
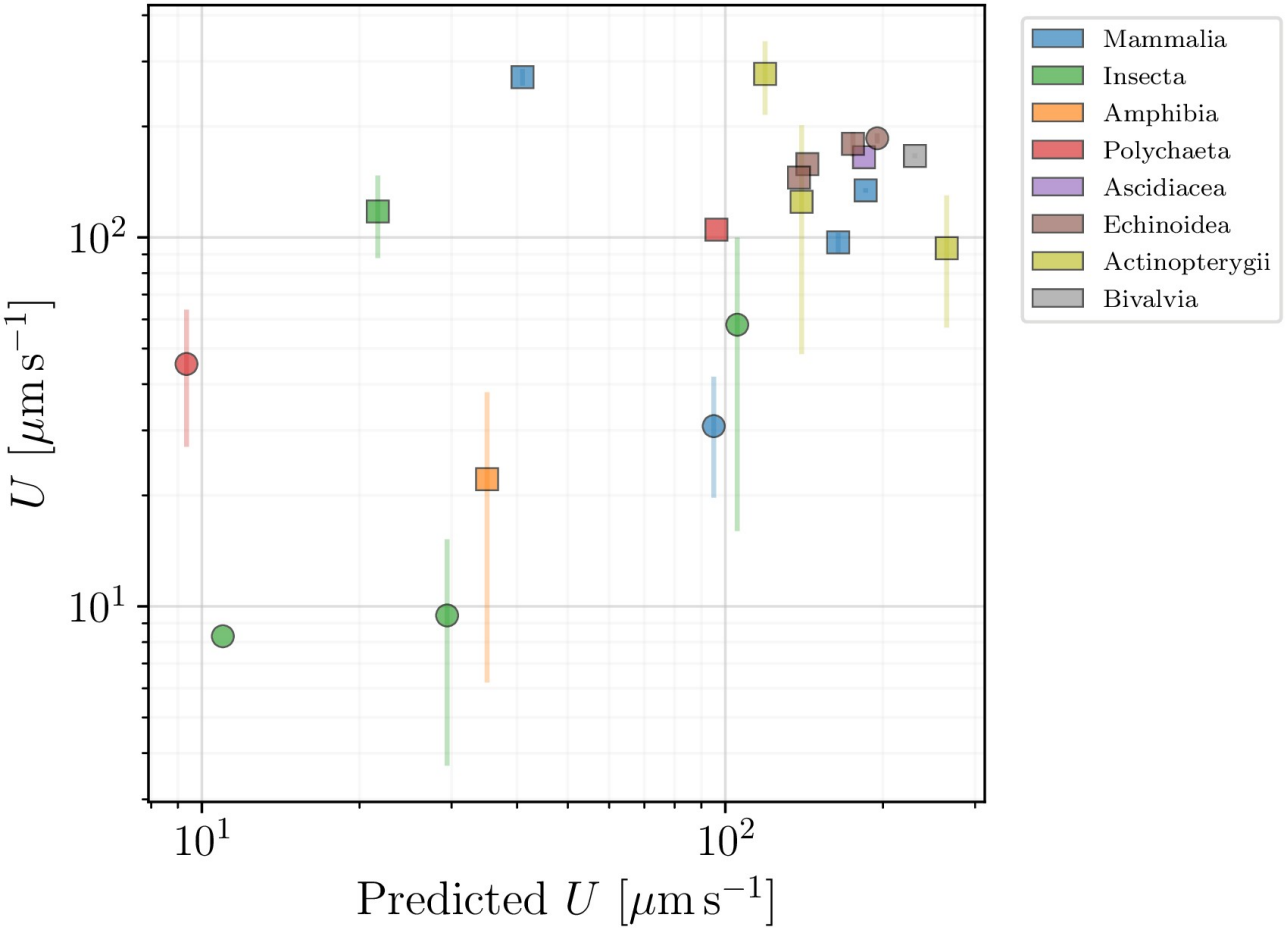
Reported propulsion speed of spermatozoa compared with the values predicted by the theoretical model in Eq (30). Colour scheme distinguishes between the different taxonomic classes. Squares represent spermatozoa that had all parameters available in the literature, while the circles mark cases where at least one parameter had to be estimated (via 〈*W*/*B*〉 = 0.47 from Fig 15 or through Eq (21)).

To help organise the information on the locomotion of sperm cells in our database, we resort to the model from Eq (30), which we compare with the collected data in Fig 18. Circles represent organisms for which either the body width *W* was unavailable (in which case we assumed *W*/*B* = 0.47, the average value from Fig 15), or for which one parameter of the flagellar wave was missing (and was thus estimated using Eq (21)). The thickness of the flagella was fixed at 2*b* = 0.4 *μ*m. We see that the model of Eq (30) is able to capture the essence of spermatozoan swimming, and better than it did for flagellated eukaryotes in the previous section. The outliers can likely be explained by the use of more complex wave patterns in some species.

## 6 Ciliates

Within the diverse group of flagellated eukaryotes, the final family of organisms is distinguished by their remarkably large number of flagella, ranging from hundreds to tens of thousands (see the distribution in Fig 2). These flagella are short compared to the size of the cell body and are called in this case cilia—hence the name of ciliates given to the whole group. Ciliates have developed a locomotive strategy relying on the phased beating of their many cilia. Typically, a single cilium beats using a two-stroke motion with a power stroke of an extended cilium followed by a recovery stroke where the cilium is curved, generating a polarised beat [10]. From the phased beat of neighbouring cilia, collective motion is induced that pumps the surrounding fluid [19], thus creating the hydrodynamic forces necessary for locomotion. This collective sequential movement of cilia is often observable through the so-called metachronal waves of deformation travelling over the surfaces of ciliated cells, resembling spectator waves in stadiums. Yet, the underlying ciliary structure is not easily observed and only a few studies report successfully the wavelengths of metachronal waves and ciliary beat frequencies. In particular, for the model organisms in the genus *Paramecium* the frequencies of ciliary beat of all the different regions of the cell have been accessed [107].

The mathematical modelling of metachronal waves can be undertaken at various levels of complexity [10, 12], starting with coarse-grained continuum models, such as the squirmer model [108, 109], up to detailed simulations of the deformations of individual cilia interacting hydrodynamically [110, 111]. Non-hydrodynamic interactions via intra-cellular coupling mediated by the cell body are also important [112, 113]. Independently of the specific coordination mechanism, ciliates all swim by transporting the surrounding fluid along their surfaces, and move in the direction opposite to the fluid motion. By using different models for this effective transport mechanism, we can now test several hypotheses across our database of ciliates, which involves data for 93 species. Note that the distribution of swimming speeds across species from this dataset has been published in our earlier contribution [39].

### 6.1 Geometry and swimming speeds of the cells

**Fig 19:**
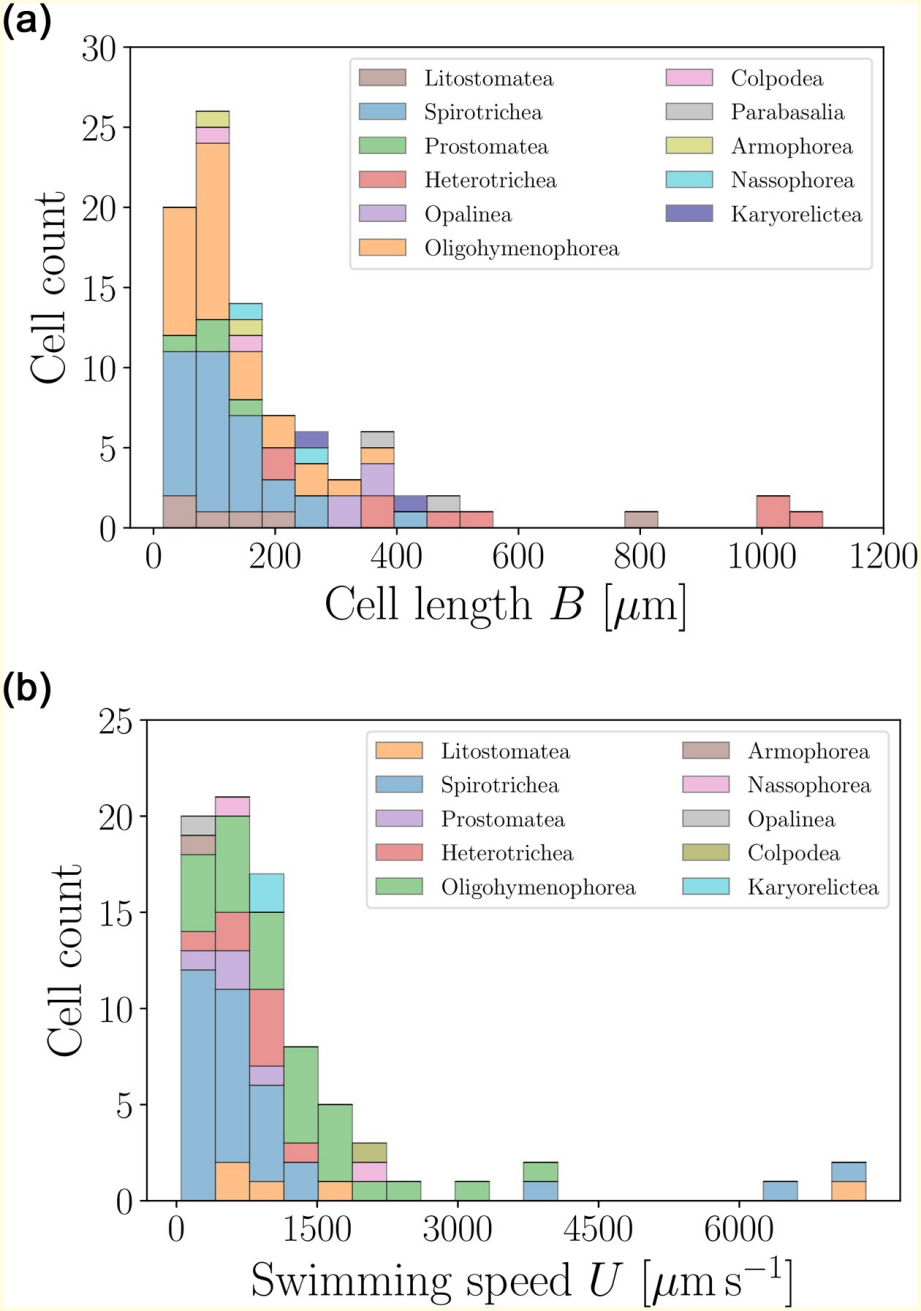
Histograms of body lengths, *B* (*μ*m, left), and swimming speeds, *U* (*μ*m s^−1^, right), for the 93 ciliates in the database. Ciliates are by far the largest organisms in our database, with the average cell length of 〈*B*〉 = 194.87 ± 207.45 *μ*m (*n* = 91), and an average swimming speed 〈*U*〉 = 1147.57 ± 1375.64 *μ*m s^−1^ (*n* = 81).

In Fig 19, we present histograms of sizes and swimming speeds for the ciliates in our database. Most of the organisms are close to, or slightly below, average values, which is highlighted by the skewness of the distributions [39]. The cells are notably larger (average length about 200 *μ*m) and faster (average speed of over one millimetre per second) than any other group in our database. As a result, the dimensionless Péclet number for relevant molecular solutes (such as ions) around the ciliates is of the order of 100 which means that, in contrast to bacteria and flagellates, ciliates live in a physical environment where advection and thus the ability to stir the surrounding fluid may be the life-driving mechanism [39].

**Fig 20:**
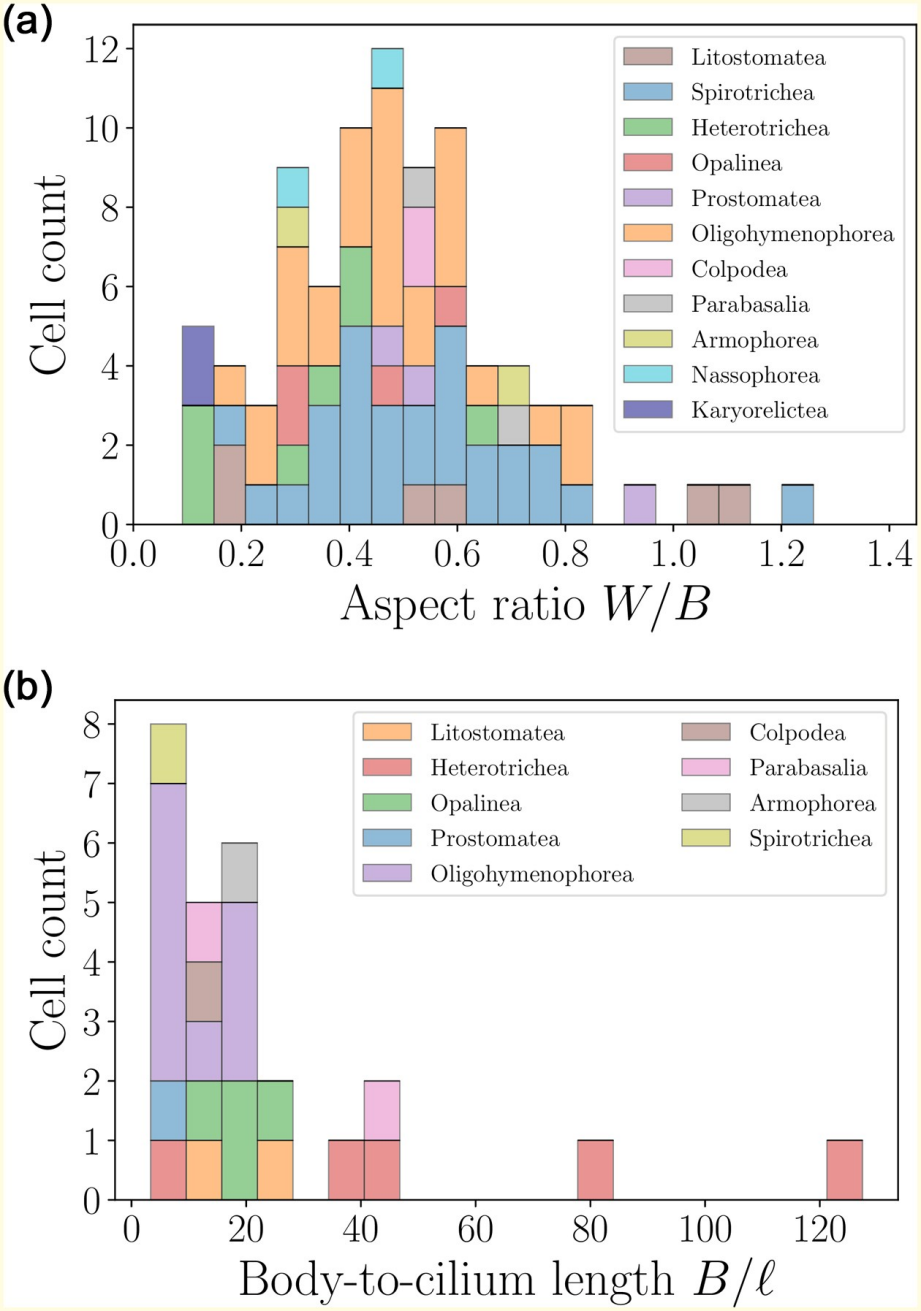
Histograms of aspect ratios *W*/*B* (left) and body-to-cilium length *B*/*ℓ* (right) for ciliates. Most of the cells are prolate, with the mean aspect ratio 〈*W*/*B*〉 = 0.49 ± 0.22 (*n* = 86). The size-to-flagellum length ratios have average values 〈*B*/*ℓ*〉 = 23.13 ± 27.03 (*n* = 26).

The distribution of aspect ratios of the cells, along with the body-to-cilia lengths, are shown in Fig 20. The former peaks at the mean value of about 0.5, indicating prolate cell bodies. The large values of the body-to-cilium length ratios confirm that cilia take the form of tiny hairs covering the cell body, much smaller than the body itself. This in turn justifies coarse-grained modelling approaches representing the cell body as a continuous surface capable of exerting stress, thereby locally averaging the collective motion of individual cilia.

### 6.2 Models for ciliary propulsion

In search of means to organise our data on the locomotion of ciliates, we propose below three distinct ciliary propulsion models that each assume a different property to be constant among the cells during forward swimming. These three approaches model the swimming of the cells as induced by: **(A)** a constant tangential stress exerted on the cell surface by the cilia array; **(B)** a constant force exerted by each individual cilium on the fluid; **(C)** a constant effective fluid speed induced near the cell surface by the cilia.

We model a ciliate cell as a prolate spheroid of length *B* and diameter *W*. We set the *x*-axis along the long axis of the cell, taken to also be the direction of movement. The ciliate swimming with speed *U* along *x* is then subject to a viscous drag of magnitude

$$\begin{array}{c} D=3\pi \eta BC_{FB}U, \end{array}$$ (31)

with the geometry-dependent coefficient *C*_*FB*_ in Eq (12). Balancing this drag with the propulsive force generated by the collective action of the cilia yields different models for the swimming speed *U*, according to how one exactly models the propulsive force.

Some aspects of the mathematical description of the cell will be useful in what follows. A cross-section of the spheroid containing **e**_*x*_ is an ellipse of eccentricity $e=\sqrt{1-(W/B{)}^{2}}$. Every point of the ellipse can be parametrised in polar coordinates by

$$\begin{array}{c} r\left(\theta \right)=\frac{W}{2\sqrt{1-{\left(e\;\text{cos}\;\theta \right)}^{2}}}, \end{array}$$ (32)

with the origin placed at the centre between its foci. Every point on the surface of the spheroidal body can then be written using spherical coordinates as

$$\begin{array}{c} \text{x}\left(\theta ,\varphi \right)=r\left(\theta \right)[\text{cos}\;\theta \;{\text{e}}_{x}+\text{sin}\;\theta (\text{cos}\;\varphi \;{\text{e}}_{y}+\text{sin}\;\varphi \;{\text{e}}_{z})],\;\theta \in \left[0,\pi \right],\;\varphi \in [0,2\pi [. \end{array}$$ (33)

One may thus write the axisymmetric, unit vector tangential to the spheroidal surface and pointing along the polar angle as

$$\begin{array}{c} \text{t}\left(\theta \right)=\frac{1}{\sqrt{r{\left(\theta \right)}^{2}+r^{\prime }{\left(\theta \right)}^{2}}}[r^{\prime }\left(\theta \right){\text{e}}_{r}+r\left(\theta \right){\text{e}}_{\theta }], \end{array}$$ (34)

where *r*′(*θ*) = d*r*(*θ*)/d*θ*. Finally, an infinitesimal surface element on the spheroidal surface is given by

$$\begin{array}{c} dS=r\left(\theta \right)\text{sin}\;\theta \sqrt{r{\left(\theta \right)}^{2}+r^{\prime }{\left(\theta \right)}^{2}}\;d\theta \;d\varphi . \end{array}$$ (35)

Let then **x** be a given point on the spheroidal surface, Eq (33). The probability of having a cilium in an area d*S* around **x** is denoted by *p*(**x**)d*S*, and we take the probability density to be uniform by setting p(x)=1/S for every **x** of Eq (33), where

$$\begin{array}{c} S=\int_{0}^{2\pi }\int_{0}^{\pi }r\left(\theta \right)\;\text{sin}\;\theta \sqrt{r{\left(\theta \right)}^{2}+r^{\prime }{\left(\theta \right)}^{2}}d\theta \;d\varphi =\frac{\pi W^{2}}{2}[1+\frac{\text{arcsin}\;e}{e\sqrt{1-e^{2}}}] \end{array}$$ (36)

is the surface area of the spheroid.

In order to proceed, we now need to balance the drag force with ciliary propulsion, and thus need to specify the details of the propulsion mechanism.

#### (A) Constant tangential stresses

The simplest model for ciliary propulsion assumes that the array of cilia exerts a constant, axisymmetric stress of magnitude *τ* along the tangent vector **t**. Using Eqs (34) and (35), the total propulsive force can then be written as

$$\begin{array}{c} P_{\tau }=\int_{S}\tau \left(-\text{t}\cdot {\text{e}}_{x}\right)dS=\tau \;I_{\text{t}}\left(B,W\right), \end{array}$$ (37)

with a purely geometric factor given by

$$\begin{array}{c} I_{\text{t}}\left(B,W\right)=2\pi \int_{0}^{\pi }[{\left(r\left(\theta \right)\text{sin}\;\theta \right)}^{2}-r\left(\theta \right)r^{\prime }\left(\theta \right)\;\text{sin}\;\theta \;\text{cos}\;\theta ]d\theta . \end{array}$$ (38)

Balancing the propulsion *P*_*τ*_ from Eq (37) with the drag *D* given by Eq (31) and solving for the swimming speed *U*_*τ*_ leads then to the theoretical model

$$\begin{array}{c} U_{\tau }=\tau \frac{I_{\text{t}}}{3\pi \eta BC_{FB}}. \end{array}$$ (39)

#### (B) Constant force per cilium

In the second modelling approach, one may imagine that each cilium, whose base lies at the point **x**(*θ*, *φ*), exerts a constant force *F* along the tangent vector **t**. One cilium then contributes a local thrust along *x* of magnitude

$$\begin{array}{c} F\left(-\text{t}\right)\cdot {\text{e}}_{x}=\frac{F}{\sqrt{r{\left(\theta \right)}^{2}+r^{\prime }{\left(\theta \right)}^{2}}}[r\left(\theta \right)\;\text{sin}\;\theta -r^{\prime }\left(\theta \right)\;\text{cos}\;\theta ]. \end{array}$$ (40)

If the ciliated cell possesses *N* such cilia, uniformly distributed over its surface, the central limit theorem establishes the total propulsive force to be

$$\begin{array}{c} P_{F}=F\frac{N}{S}I_{\text{t}}\left(B,W\right). \end{array}$$ (41)

After balancing with the drag, this leads to the ciliary swimming speed *U*_*F*_ predicted by this model as

$$\begin{array}{c} U_{F}=F\frac{NI_{\text{t}}}{3\pi \eta BSC_{FB}}. \end{array}$$ (42)

#### (C) Constant surface velocity

The third modelling approach assumes that the local speed of the fluid induced by ciliary motion is (almost) constant. To quantify this hypothesis, consider a spheroidal cell with a prescribed tangential surface velocity distribution **u**_s_ = *u*_s_(*ζ*) **t**, where *ζ* = cos *θ* and **t** is given by Eq (34).

In this case, the Lorentz reciprocal theorem may be used to relate the propulsion speed *U*_s_ of a squirming organism to the surface velocity distribution [114, 115] by

$$\begin{array}{c} U_{\text{s}}=-\frac{\tau_{0}}{2}\int_{-1}^{1}(\frac{1-\zeta^{2}}{\tau_{0}^{2}-\zeta^{2}}{)}^{1/2}u_{\text{s}}\left(\zeta \right)\;d\zeta , \end{array}$$ (43)

where $\tau_{0}=1/e=1/\sqrt{1-(W/B{)}^{2}}$ is fixed by the morphology of the swimmer.

Following past work [114], if we take an almost uniform surface velocity distribution of the form

$$\begin{array}{c} u_{\text{s}}\left(\zeta \right)=-\tau_{0}{\hat{u}}_{\text{s}}(\frac{1-\zeta^{2}}{\tau_{0}^{2}-\zeta^{2}}{)}^{1/2}, \end{array}$$ (44)

where the constant ${\hat{u}}_{\text{s}}$ sets the characteristic surface velocity scale, we obtain a model for the swimming speed as given by $U_{\text{s}}={\hat{u}}_{\text{s}}\left[\tau_{0}^{2}-\tau_{0}(\tau_{0}^{2}-1){\text{coth}}^{-1}\tau_{0}\right]$, which may also be written in terms of the eccentricity *e* as

$$\begin{array}{c} U_{\text{s}}\left(e\right)=\frac{{\hat{u}}_{\text{s}}}{e^{2}}(1-\frac{1-e^{2}}{e}{\text{tanh}}^{-1}e). \end{array}$$ (45)

With this particular flow assumption, for a very slender cell body (*e* → 1), $U_{\text{s}}\to {\hat{u}}_{\text{s}}$, while for a spherical cell (*e* → 0) we get $U_{\text{s}}\to 2{\hat{u}}_{\text{s}}/3$, in agreement with the classical result [116].

### 6.3 Insights from data

**Fig 21:**
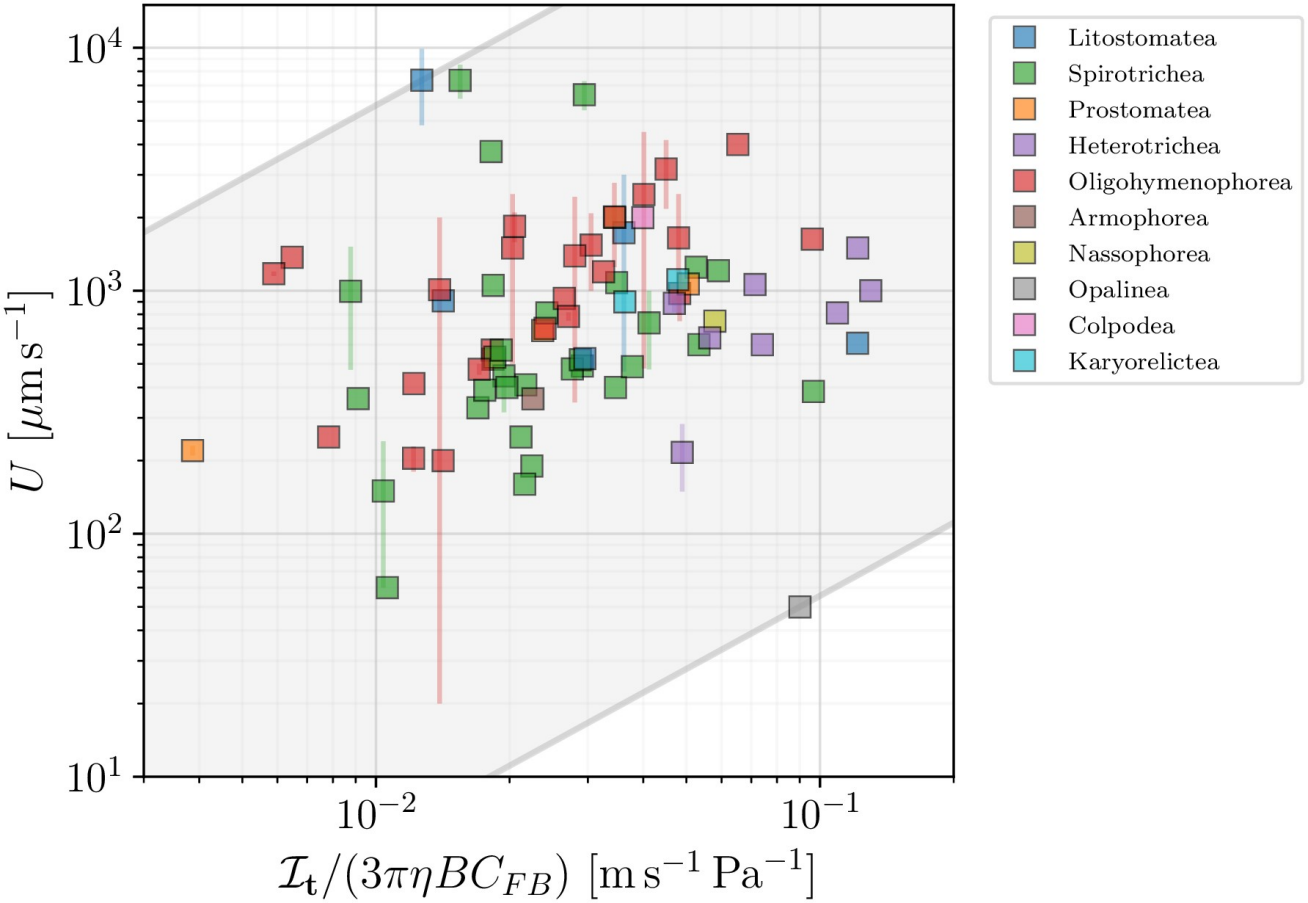
The swimming speed *U* for ciliates plotted versus the numerical factor accompanying the constant tangential stress assumed in model (A) and Eq (39). The shaded area encloses all organisms and serves as an estimate of the average effective tangential stress for all organisms, with the lower bound of *τ*_min_ = 0.55 mPa, and the upper bound of *τ*_max_ = 580 mPa. Colours distinguish between classes of ciliated organisms. The scatter of data suggests that only a large range of values for the stress of individual organisms can be inferred.

We begin with the constant tangential stress model **(A)**, where the swimming speed is given by Eq (39). In Fig 21 we plot the measured speed for all ciliated species in our database versus the factors accompanying the tangential stress *τ* in Eq (39). The scatter of the data points clearly does not support the hypothesis of universal surface stress for all organisms. The model can however be used to estimate the effective stress *τ* on the surface of each ciliate in the database. The shaded area represents the bounds for *τ*, and fall in the wide range 0.55 − 580 mPa. These values are consistent with the estimate of *τ* ≈ 10 mPa for *Volvox* colonies [117].

**Fig 22:**
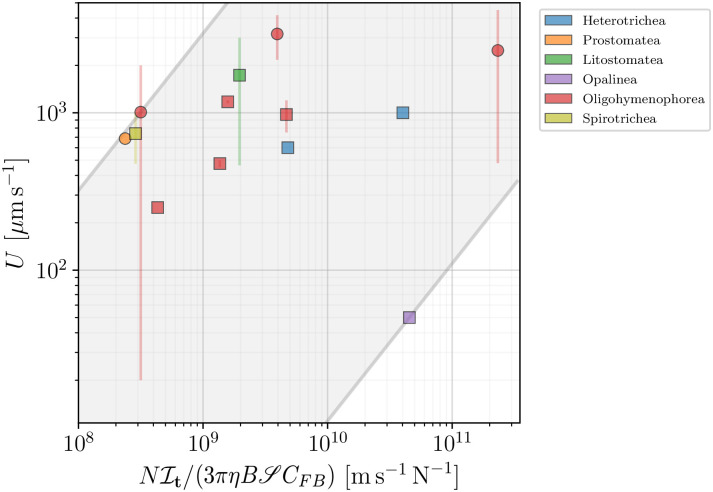
Reported swimming speed *U* plotted against the numerical prefactor of Eq (42), assuming a constant effective force per cilium in the propulsion model (B). Square symbols mark organisms for which the prediction was directly calculated from the available data, while circles represent those for which we estimated the number *N* of cilia. Colours distinguish the different taxonomic classes. The visible large scatter of data sets the bounds for the effective force per cilium to be in the range of 1.10 10^−3^ to 3.19 pN, represented by the shaded area in grey.

A similar comparison for the ‘constant force per cilium’ model **(B)**, quantified by Eq (42), requires the knowledge of the number of cilia *N* for each swimmer. This number is, however, scarcely reported in literature, with only 9 values registered in our database. For some species, however, measurements report the number of cilia per unit area *κ*, or the distance between neighbouring cilia *d*. Using the latter, we can estimate the number of cilia per unit area to be *κ*_*d*_ ≈ 1/*d*^2^. Using Eq (36), *κ* and *N* can be easily related via N=κS. By doing so, we determined *N* (equivalently *κ*) for a total of 15 ciliated species out of 93, 13 of which had information about the cell swimming speed. In Fig 22 we plot the reported swimming speed versus the right-hand side of Eq (42) to estimate the effective force per cilium *F*. We report our estimated values of *F* for each species in Table 2. Our data encloses previous estimates in the range 0.3 − 1.0 pN [118], and show that the effective tangential forces exerted by each cilium may even be two orders of magnitude lower for species like *Opalina ranarum*.

**Table 2:** Estimated values of the effective tangential force *F* exerted by each cilium for the species in Fig 22.

Species	*F* [pN]
*Blepharisma* sp.	1.25 10^−1^
*Coleps hirtus*	2.89
*Didinium nasutum*	8.82 10^−1^
*Opalina ranarum*	1.10 10^−3^
*Paramecium caudatum*	1.07 10^−2^
*Paramecium multimicronucleatum*	8.04 10^−1^
*Paramecium* spp.	2.09 10^−1^
*Spirostomum* sp.	2.49 10^−2^
*Stylonichia* sp.	2.57
*Tetrahymena pyriformis*	3.48 10^−1^
*Uronema marinum*	3.19
*Uronema* sp.	7.43 10^−1^
*Uronemella* spp.	5.78 10^−1^

**Fig 23:**
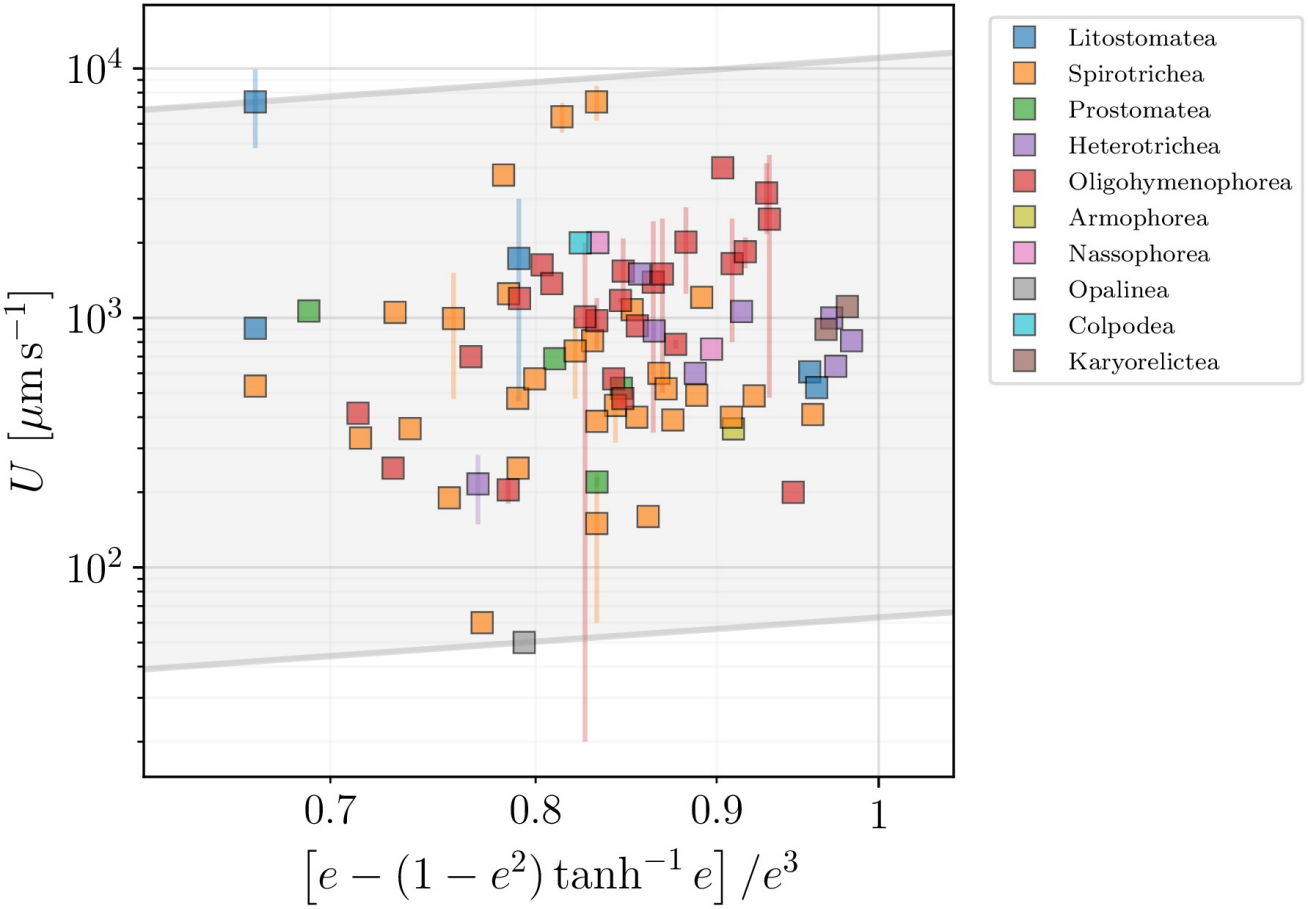
Reported swimming speeds from our database *U* plotted against the geometric factor from Eq (45) for the constant-flow model (C). The data can be used to estimate the range of effective surface velocities to be in the range from 63.0 *μ*m s^−1^ to 1.10 10^4^ *μ*m s^−1^, in the grey shaded area. Colours allow to distinguish different taxonomic classes.

The third model **(C)** assumes the creation of local flows by an almost constant surface velocity, whose order of magnitude is fixed by ${\hat{u}}_{s}$. The predictions of Eq (45) suggest that the swimming speed and the surface velocity are related by a simple geometric parameter, namely a function of the cell body eccentricity, *e*. In Fig 23 we plot the measured ciliate velocities against the theoretical geometric factor determined for each species from our data. The model can be used to estimate the magnitude of the effective average surface speed for each species. The resulting values span from a few tens of *μ*m s^−1^ to about 10^4^ *μ*m s^−1^. The average value of the effective surface velocity, calculated for all species, $\langle {\hat{u}}_{\text{s}}\rangle =1.42\;10^{3}$ *μ*m *s*^−1^, is about 2 to 3 times the average metachronal wave speed we estimate from our data, λ_*MW*_ *f*, where λ_*MW*_ is the wavelength of the metachronal wave created by the collective ciliary beating at frequency *f*. Here also our data confirm and extend previous estimates. For example in Ref. [119], tracking microscopy and fluid velocimetry were used to determine with precision the flow field of a freely swimming *Volvox* colony, resulting in estimates of the surface speed ${\hat{u}}_{\text{s}}\approx 200-250$ *μ*m s^−1^ for species swimming at *U* ≈ 100 − 150 *μ*m s^−1^.

## 7 Conclusion

### 7.1 Summary and perspective

In this paper, based on an initial selection of six seminal papers in the field of biological fluid dynamics and physics, we assembled a summary of the experimental data produced to date on the characterisation of motile behaviour of unicellular microswimmers. The material gathered provides a convenient and practical reference point for future studies. Our database includes empirical data on the motility of four categories of organisms, namely bacteria (and archaea), flagellated eukaryotes, spermatozoa and ciliates. Whenever possible, we reported the following biological, morphological, kinematic and dynamical parameters: species, geometry and size of the organisms, swimming speeds, actuation frequencies, actuation amplitudes, number of flagella and properties of the surrounding fluid. In all cases, we also give the appropriate references to the publications reporting the measurements. We then analysed this information by characterising some of the statistical properties of the cells in our database and by introducing theoretical models for each main species in order to establish guiding principles for the presentation of the data. We particularly focused on the dependence of the swimming speed on the characteristics of the swimmers and environmental properties. The analysis shows that qualitative trends established in the theoretical framework based on motility in Stokes flows agrees broadly with the reported data but that the large degree of variability among species precludes drawing general conclusions from the dataset. The modelling approaches can however be helpful in rationalising the data, pointing out the relevant dynamic quantities governing the locomotion of each individual group. In particular, our data confirm and extend estimates of these parameters previously reported in the literature.

An important result highlighted by our study is that a tremendous statistical variability exists in the available data, not only within domains [39] but also within smaller taxonomic groups. Little is known about the variability of motility within individual species in Nature, neither in terms of their morphological characteristics (e.g. size and shape distribution), nor in the details of their propulsion (flagellar or ciliary motion). In fact, for every single set in our database, it is not clear at all how representative any particular measurement is of a group of similar organisms in the same environment? How sensitive are the propulsion characteristics of these cells to changes in the environmental stimuli and how do they adapt to new conditions? With the enhanced capacity to process large datasets and with new developments regarding automation of image analysis, the task of gathering and processing statistical data is becoming increasingly feasible, and new works will be able to discover the fundamental principles dictating the locomotion of similar species within the same taxonomic group.

The database in its current form, which is stored on the OSF repository [54], would benefit from the collaborative effort of the community. By growing further, it would help provide up-to-date information on the dynamics of a variety of organisms and populations, hopefully further encouraging collaborations between cell biologists and physical scientists. To aid this process, our database is available on GitHub [55], where it can be extended and enriched.

We gave our database the appellation of BOSO-Micro. The first term stands for Bank Of Swimming Organisms while the second is there to emphasise that we have focused our work on microscopic unicellular organisms. We hope that new versions of the database, BOSO-X, will be built by focusing for X on different organisms. An obvious suggestion would be to assemble a BOSO-Fish database, given the large amount of experimental, computational and theoretical knowledge on the swimming of fish. We hope that building exhaustive databases of this sort will further facilitate the work of physical scientists on biological problems related to locomotion.

### 7.2 Caveats and limitations

The collection of data gathered in our database is inevitably incomplete and biased, in particular due to the way the initial set of literature sources, focused on biophysical studies, was chosen. Despite our efforts to carry out a broad search for swimming data, it is possible that important references were left out. The mitigation strategy in this case relies on making the database public [54] and expandable [55].

Regarding the presented data, a major limitation is of course their sparseness. The relevant parameters in the description of motility are incomplete for many species, especially the variables related to the beating of cilia and flagella, which hinders direct comparison with theoretical models.

Furthermore, the database was populated using data presented across different papers, books, registers and reports, and the multiplicity of sources introduces a significant and inherent noise. For many species, reported measurements of one or more characteristics refer to different experimental environments. Even if those are reported, different strains of the same species may behave differently under slightly modified physical and chemical conditions.

It is also important to highlight the limitations and assumptions of the models used in our paper. The models were designed to assist the presentation of data in the context of established ideas regarding microscale locomotion, and to provide quick estimates of the relevant dynamic characteristics of microswimmers. Common to all the models is our assumption that the cell bodies are spheroidal. A look at Fig 3 quickly reveals that this hypothesis is a crude approximation for many species in our analyses (e.g. *Caulobacter crescentus*, *Ceratium tripos*, *Stentor*). We have made this choice in modelling in order to account for the influence of both the cell body length and width in an analytical way. The diversity of form, which might be crucial for certain locomotion strategies, has no reflection in the considered simplistic models, yet it must be incorporated into specific models describing particular organisms. Similarly, in the case of swimming eukaryotic cells, several of our hypotheses on the flagellar beat ought to be examined carefully. For spermatozoa and flagellated eukaryotes, we assumed the form of a simple sinusoidal wave, whereas many species display more complex flagellar beating patterns (e.g. complex waves displayed in *Columba livia* and *Sturnus vulgaris* spermatozoa). For flagellated eukaryotes, we have neglected hydrodynamic interactions between flagella, which is a simplified approximation. In the case of ciliates, the three models we have introduced also do not take into account hydrodynamic interactions between neighbouring cilia, nor the effect of the polarised beating of cilia and their recovery stroke. Despite these limitations, we hope that the use of modelling may also prove useful in rationalising and organising future data on swimming organisms along similar lines.

## 8 Appendix

### A The database of swimming microorganisms

In Table 3 we present a short glossary with the main symbols used in the database.

**Table 3:** List of symbols used in the database, together with their explanation and units.

Symbol	Meaning	Unit
*B*	Body length	*μ*m
*W*	Body width	*μ*m
*N*	Number of flagella or cilia	-
*L*	Lengths (mostly flagella, otherwise specified)	*μ*m
*n*_*w*_	Number of waves (full periods, or crests) produced by flagellar beat	-
λ	Wavelength of flagellar waves (of helicoidal body and of metachronal waves indicated by a subscript *B* and *MW*, respectively)	*μ*m
Λ	Length of a complete wave along the flagellum (or path, indicated by subscript)	*μ*m
*h*	Amplitude of waves (for helicoidal bodies, a subscript *B* added)	*μ*m
*U*	Swimming velocity	*μ*m s^−1^
*ω*	Flagellar beat frequency	s^−1^
Ω	Frequency of the rotation of cell body	s^−1^
*c*	Wave speed of flagellar beat (or metachronal wave)	*μ*m s^−1^
*V*	Volume of cell body	*μ*m^3^
*ℓ*	Length of cilia	*μ*m
*d*	Distance between cilia	*μ*m
*b*	Radius of flagella	*μ*m
*κ*	Number of cilia per unit area	*μ*m^−2^
*f*	Beating frequency of cilia	s^−1^
*G*	Gyration (frequency at which organisms revolve around the axis of movement)	s^−1^
*η*	Viscosity of the swimming medium	mPas

For every entry in the database, in the case when more than one measurement was available, we report the average value and the standard deviation using the ± notation. Values inside parentheses specify the range of the values measured, e.g. (*x*_min_ − *x*_max_). Sometimes only the upper boundary was available, indicated by a preceding ‘max’. When the information was not available in the texts of the articles, the figures or the graphics were analysed with the GNU Image Manipulation Program (GIMP) software in order to extract data. This is indicated in the tables by a superscript ^⋆^ or ^×^ respectively, if figures or graphics were used.

The various tables of data are organised as follows. Table 4 contains the data for 78 organisms in the branch of bacteria (with 5 spiral-shaped bacteria included). Spirochaetes (18 species) and *Spiroplasma* (2 species) were separated from the other bacteria because of their distinct mode of locomotion and are presented in Table 5. The data for the 10 species of archaea are contained in Table 6.

**Table 4:** Data for swimming bacteria (Spirochaetes and *Spiroplasma* excluded).

Species	Geometry			Kinematics		References
	*B*	*W*	Flagella	*U*	Notes	
*Agrobacterium sanguineum*				25.2 (max35)	Mean run time = 0.11 s with acceleration = 138 *μ*m s^−2^.	[120]
*α*-proteobacte-rium AB015520^†^			^†^GenBank closest matching organism.	17.25 ± 4.05 (max55)	Mean run time = (0.19 − 0.21) s with acceleration = (96 − 124) *μ*m s^−2^.	[120]
*α*-proteobacte-rium KAT8	4	1		22 (max28)	GenBank AF025321. *V* = 3.1. Mean run time = 3.05 s.	[121]
*Alteromonas macleodii*	(2 − 7)	0.4	Monopolar flagellum.	19 ± 2.9 (max 55)	Mean run time = (0.13 − 0.2) s with acceleration = (112 − 139) *μ*m s^−2^.	[120, 122, 123]
*Arthrobacter histidinolovorans*		0.26		23.3 (max 55)	Mean run time = 0.19 s with acceleration = 166 *μ*m s^−2^.	[120]
*Azospirillum brasilense*	2.61^⋆^	0.9^⋆^	Single thick polar flagellum and ca. 22 thin lateral flagella. *L*_polar_ > 5.2^⋆^, *L*_lateral_ = (3.1 − 4.51)^⋆^, *n*_*w*polar_ > 4^⋆^, *n*_*w*lateral_ = (5 − 6)^⋆^, λ_polar_ = 1.36^⋆^, λ_lateral_ = 0.65^⋆^, *h*_polar_ = 0.13^⋆^, *h*_lateral_ = 0.06^⋆^.	(13 − 23)	Strain ATCC 29145. *U* up to 100 *μ*m*s*^−1^ has also been reported.	[124, 125]
*Azospirillum lipoferum*	2.24 ± 0.32	1.4 ± 0.3	Single polar flagellum and/or lateral flagella.	26.9 ± 2.7^×^	Strain ATCC29707.	[125, 126]
*Azotobacter vinelandii*	(3 − 5)	(1.6 − 2.5)	Peritrichous flagella. λ = (2 − 3), *h* = (0.4 − 0.59).	13.1^†^(8.7^‡^ − 74)	^†^Wild-type strain DJ. ^‡^Strain DJ77.	[127–129]
*Bacillus licheniformis*	(1.5 − 3)	(0.6 − 0.8)	Peritrichous flagella. λ = (2.2 − 2.6).	21.4^†^	Strain 9945-A, grown at 30°C. ^†^At 20°C	[130–132]
*Bacillus megaterium*	3 (2 − 5)	(1.2 − 1.5)	Peritrichous flagella. *N* = (26 − 36), *n*_*w*_ ≈ 2.5, λ = 3.389 ± 0.166^†^, *h* = (0.46 − 0.53)^‡^.	(22.2^♢^ − 47.2^♣^)	Swimming speed was studied in function of viscosity. Chamber kept between 19 and 25°C. ^†^Average from 4 strains. ^‡^The value of λ was used to make the estimate. ^♢^ *η* = 1.16. ^♣^ *η* = 4.7.	[25, 128, 131–133]
*Bacillus subtilis*	(2 − 4)	(0.7 − 0.8)	*N* ≈ 12, *L* = 7.5, λ = 2.186 ± 0.103^†^.	(20 − 32^‡^)	Strain BC26 grown at 35°C. ^†^Average of 6 strains. ^‡^At 30°C, pH between 6 and 7.5.	[128, 132, 134, 135]
*Bdellovibrio bacteriovorus*	1.48^⋆^^†^	0.58^⋆^^†^	*N* = 1, *L* = 4 ± 0.5, *n*_*w*_ = 1, λ = 0.565^⋆^^†^, *h* = 0.23^⋆^^†^.	(35^†^ − 160^‡^)	([19] report Ω = 600, measured with flagellum tethered). ^†^Strain 109J. ^‡^Strain HD100.	[19, 136, 137]
*Bradyrhizobium japonicum*	(1.62 − 1.74)^⋆^	(0.62 − 0.73)^⋆^	The cell has a thick flagellum (diameter 22nm) and a few thin flagella (12nm). Bases of the thick flagellum distribute at one end of the cell from 10 to 26% of the cell length (average from 35 cells). The average ratio is 18.7%. The bases of the thin flagella distribute widely from 9 to 44% with an average of 23.5%. λ_thick_2.8 ± 0.3^⋆^, λ_thin_ = 0.7 ± 0.04^⋆^.	30.4 ± 5.7^†^	Swimming speeds of the wild-type cells (^†^) and those with a thick flagellum are almost the same (30.3 ± 2.9, strain BJDΔ283), but cells with only thin flagella (BJDΔ293) are much slower (16.8 ± 6.1), with an aberrant and unstable pattern of movement.	[138]
*Campylobacter jejuni*^†^	(0.5 − 5)	(0.2 − 0.5)	*N* = (1 − 2), *L* = (1 − 15), λ = (1.54 − 1.63)^⋆^, *h* = (0.34 − 0.38)^⋆^; λ_*B*_ = (0.96 − 1.12)^†^, *h*_*B*_ = (0.23 − 0.48)^†^.	64.8 ± 14.9 (39.3 − 100.2)^‡^	^†^Helical-shaped. ^‡^Average of 5 strains (FUM158432, 600, MQ23, MQ26 and VIC) with *η* ≈ 1. Speeds are available in function of *η* for all of them.	[26, 125, 139, 140]
*Candidatus* Ovobacter propellens	(4 − 5)		*N* ≈ 400 forming a prominent tuft that bends backwards and rotates CCW, leading to a right-handed, helical swimming path^†^.	(600 − 700)^‡^	*ω* = (100 − 200), Ω = (50 − 100). ^†^ *h*_path_ = (2-3), *h*_path_ = (5-10) ^‡^Some cells may attain *U* = 1000.	[141]
*Caulobacter crescentus*	1.6	(0.4 − 0.6)	*N* = 1, *L* = (5.3 − 6.6), λ = 1.08, *h* = 0.13.	41.3 ± 7.3^†^	*ω*_motor_ = 310 α 47. The authors also measured the torque as 342 ± 42 pN nm. ^†^For wild-type cells swimming in water.	[142, 143]
*Chromatium okenii*	(8 − 16)	(4.5 − 6)	Lopotrichous flagella. *N* = 40, *L* = 25.	45.9^†^	^†^At 20°C, strain from R. L. Gherna.	[129, 130, 144]
*Clostridium tetani*	6	0.5	*N* ≈ 15, *n*_*w*_ ≈ 4, λ = 1.8, *h* ≈ 0.42.	(0.8 − 11.2)^†^	^†^Swarming.	[70, 131–133, 145]
*Colwellia demingiae*	(1.5 − 4.5)	(0.26 − 0.6)		21.75 ± 4.85 (max 65)	Mean run time = (0.15 − 0.16) s with acceleration = (106 − 135)*μ*m s^−2^.	[120, 146]
*Curacaobacter baltica*	2.5	1		21 (max 30)	Average run length = 6.2s. *V* = 2.	[121]
*Escherichia coli*	2.5 ± 0.6	0.88 ± 0.09	Peritrichous flagella. Usually *N* = 6 (*N* = 3.37 ± 1.59^†^^‡^), *L* = 8.3 ± 2.0^†♢^ (*L* = 7.3 ± 2.4^†‡^), *n*_*w*_ ≈ 2, λ = 2.366 ± 0.121^♣^, *h* = 0.38^†‡⋆^.	24.1 ± 10 (14.2 − 60)^♠^	*ω* = 131 ± 31^†‡^(also *ω* = 270^♯^), Ω = 23 ± 8^†‡^, *ω*_motor_ = 154 ± 30^†‡^. Additional parameters measured^†^: twiddle length = 0.14 ± 0.19 s, run length = 0.86 ± 1.18 s, change in direction from run to run = 68 ± 36°, change in direction while running = 23 ± 23°. ^†^For wild-type strain AW405. ^‡^Observed in the presence of Alexa Fluor 532. ^♢^In the presence of motility buffer, at 23°C. ^♣^Average from 4 strains. ^♠^Average from values of 10 articles. ^♯^For strain HCB437 at 32°C.	[25, 26, 29, 36, 37, 128, 130, 131, 133, 144, 147–155]
*Flavobacterium uliginosum*		0.225 ± 0.035		22.45 ± 9.75 (max 65)	Mean run time = (0.16 − 0.17) s with acceleration = (80 − 117)*μ*m s^−2^.	[120, 156]
*Frigobacterium* sp.	3	1		26 (max 34)	Strain GOB (GenBank AF321022). Average run length = 10.5s. *V* = 2.4	[121]
*Haererehalobacter ostenderis*				15.4 (max 35)	Mean run time = 0.16 s with acceleration = 101*μ*m s^−2^.	[120]
*Halomonas meridiana*	(1.9 − 4.5)	(0.34 − 1.0)	*N* = (1 − 2) lateral flagella.	14.1 (max35)	Mean run time = 0.17 s with acceleration = 98*μ*m s^−2^.	[120, 157]
*Helicobacter pylori*^†^	(2.5 − 5)	(0.5 − 1)	*N* = (4 − 6), *L* = 3.2, λ = 2.1, *h* = 0.28; λ_*B*_ = 1.65^⋆^, *h*_*B*_ = 0.11^⋆^.	25 ± 4.3^♢^	The authors estimated the torque as being 3600 pN nm. ^†^Helical-shaped. ^♢^Average of two reported results.	[158–160]
*Listeria monocytogenes*	(1 − 2)			(0.113 ± 0.050^†^ − 0.115 ± 0.046)^‡^	^†^In the cytoplasm of MTF-16 (vimentin -/-) mouse 3T3 fibroblasts. ^‡^In the cytoplasm of MTF-6 (vimentin +/+) mouse 3T3 fibroblasts.	[161]
*Macromonas bipunctata*	(8 − 12)	(3 − 5)	Monopolar flagellum. *L* = (10 − 15).	10		[129, 133]
*Macromonas mobilis*	20 (12 − 30)	9 (8 − 14)	Monopolar flagellum. *L* = (20 − 40).	13.3		[129, 133]
*Magnetococcus marinus* MO-1	1.85 ± 0.4	1.33 ± 0.19	*N* = 14 in two sheathed bundles of 7 flagella each on the long axis side of the body.	98.9 ± 39.5 (max 300)		[162, 163]
*Magnetospirillum gryphiswaldense*	(2.14 − 5.15)^⋆†^	(0.34 − 0.49)^⋆†^	Bipolar flagella. *L* = 2.4^⋆^, λ = 0.35, *h* = 0.02.	(15 − 45)^‡^	Strain MSR-1 grown at 22°C. ^†^Cells can be either curved or elongated and helicoidal. ^‡^Velocity was a bimodal distribution with peaks corresponding to 15 *μ*m s^−1^ (slow mode) and 45 *μ*m s^−1^ (fast mode) in the presence of a magnetic field (1.5 mT), 50× greater than the earth’s.	[31, 164, 165]
Marine bacterium TW-3	2	0.8		44 ± 1(max56)	*V* = 1. Mean run length = 0.4s. GenBank AY028198.	[121, 166]
*Marinobacter* sp.		0.26		18.65 ± 0.75 (max 55)	Mean run time = (0.13 − 0.2) s with acceleration = (98 − 100)*μ*m s^−2^.	[120]
*Marinobacter* strain PCOB-2	3.5	1		29 (max 55)	*V* = 2.7.	[121]
*Marinocaulobacter* sp.				12.95 ± 1.65 (max45)	Mean run time = (0.12 − 0.18) s with acceleration = (89 − 92)*μ*m s^−2^.	[120]
*Marinomonas vaga*		0.375 ± 0.035		22.9 ± 0.6 (max 55)	Mean run time = (0.18 − 0.19) s with acceleration = (125 − 148)*μ*m s^−2^.	[120]
*Marinoscillum furvescens*	(10 − 50)	(0.2 − 0.5)		32 ± 1	Strain M58792	[166–168]
*Microscilla furvescens*	3.5	1		32 (max51)	Average run length = 14.9s. *V* = 2.7	[121]
*Myxococcus xanthus*	(4 − 8)	(0.7 − 0.8)		(0.03 − 0.06)^†^	^†^Swarming	[125, 169]
*Oleiphilus messinensis*	5	1		22 (max 26)	*V* = 3.9.	[121]
*Photobacterium phosphoreum*	1.2		*N* = 1, *n*_*w*_ ≈ 2.5^⋆^, λ = 3.1^⋆^, *h* = 0.37^⋆^.			[131, 133]
*Photobacterium profundum*	2.94^⋆^	1.76^⋆^	Probably monopolar. *L* = 9^⋆^, *n*_*w*_ ≈ 3.25^⋆^, λ = 2.22^⋆^, *h* = 0.39^⋆^.	(25.8^†^ − 28.2^‡^)	Data for strain SS9. This species’ motile behaviour was observed as a function of the pressure in the observation chamber *p*. ^†^At 20°C and *p* = 0.1 MPa. ^‡^At 20°C and *p* = 30 MPa. Motility ceased when *p* was superior to 170 MPa. For strain 3TCK: *U*_max_ = 21.7*μ*m*s*^−1^ at 20°C and *p* = 0.1MPa, no motility observed if *p* > 150 MPa.	[29]
*Polaribacter irgensii*	(0.8 − 48)	(0.25 − 0.5)	Polar flagella.	24.6 (max 55)	Mean run time = 0.23 s with acceleration = 178*μ*m s^−2^.	[120, 170]
*Pseudoalteromonas citrea*	(1.5 − 4.5)	(0.41 − 1.5)	Monopolar flagellum.	32.2 (max75)	Mean run time = 0.17 s with acceleration = 145*μ*m s^−2^.	[120, 122]
*Pseudoalteromonas* spp.	(1.8 − 3)	(0.19 − 1.5)	Single unsheathed polar flagellum.	32.633 ± 4.245 (max75)	Mean run time = (0.17 − 0.2) s with acceleration = (138 − 160)*μ*m s^−2^.	[120, 171]
*Pseudoalteromonas tetraodonis*	2.4	(0.34 − 1)	Monopolar flagellum.	34.7 (max 75)	Mean run time = 0.23 s with acceleration = 158*μ*m s^−2^.	[120, 172]
*Pseudomonas aeruginosa*	1.5	0.5	Monopolar flagellum. *L* = 4.84^⋆^, *n*_*w*_ ≈ 2.5^⋆^, λ = 1.53 ± 0.086^†^, *h* = 0.26^⋆^.	51.3 ± 8.4 (32.7 − 71)^‡^	^†^Average from 3 strains. ^‡^Average of the values for the strains No.3, No.6, P15, P28 and K, cultured in nutrient broth with aeration at 37°C and observed at 30°C.	[25, 38, 128–131, 139, 151, 152]
*Pseudomonas azotoformans*		0.19		18.8 (max 45)	Mean run time = 0.19 s with acceleration = 102*μ*m s^−2^.	[120]
*Pseudomonas fluorescens*	3.1 ± 0.8	0.9 ± 0.1	*N* = 1.5 ± 1.1, *L* = 8.4 ± 1.3, *n*_*w*_ = 2.5, λ = 1.76, *h* = 0.39.	77.6 (max 102)^†^	Ω = 2.4. SBW25. ^†^Run speed, other phases of motion differ.	[173]
*Pseudomonas putida*	(1.88^⋆^ − 2)	(0.87^⋆^ − 1)	*N* = (5 − 7), at one end of the cell (occa-sionally N = 1 to 12). *L* = (5.52 − 5.9)^†^, *n*_*w*_ ≈ (1 − 2)^†^, λ = 3.14^†^, *h* = 0.73^†^.	(27.5 − 75)^‡^	^†^Data from a micrography of strain PRS2000 with 5 flagella. ^‡^Data for the strain PRS2000. The average velocity was between 27.5 and 44 and the maximum velocity between 53.8 and 75. The swimming speed of the strain KT2440 was studied as a function of the optical seeding density.	[174, 175]
*Rhizobium lupini*			*N* = (2 − 3) complex flagellar filaments with *n*_*w*_ ≈ (2 − 3), λ = 2.28, *h* = 0.6.	52.4		[176]
*Rhizobium* sp.	2	0.8		22 (max 30)	Strain SDWo52 (GenBank AF345550). Average run length = 2.07s. *V* = 1.	[121]
*Rhodobacter sphaeroides*	(2.91^⋆^ − 3)	(1.47^⋆^ − 2.2)	Single sub-polar flagellum. *L* = 9.95^⋆^ (or (2 − 5) × *B*), *n*_*w*_ ≈ 3^†^, λ = 2.2, *h* = 0.7. Only clockwise rotation was observed.	15.45 ± 6.9 (max 80)^‡^	Ω = 2.7 ± 1.6. ^†^Roughly. ^‡^Average from two articles for strain WS8. Speed studied as a function of pH.	[125, 177–179]
*Roseobacter litoralis*	(1 − 2)	(0.6 − 0.9)	*N* ≥ 3^⋆^ sub-polar flagella.	24.43 ± 6.74 (max 75)	Mean run time = (0.16 − 0.18) s with acceleration = (94 − 146)*μ*m s^−2^.	[120, 180]
*Salmonella enteditidis*	(2 − 5)	(0.7 − 1.5)	Peritrichous flagella. λ = 2.335 ± 0.088^†^.	(30.2^‡^ − 40^♢^)	Strain JOR2 incubated at 37°C. Motility studied in function of viscosity. ^†^Average from 3 strains. ^‡^ *η* = 1.3. ^♢^ *η* = 3.2.	[26, 128]
*Salmonella paratyphi*	(3 − 4)	0.6	λ = 2.34 ± 0.078^†^.	25.68 ± 4.64^‡^	^†^Average from 6 strains. ^‡^For Paratyphi A, average from 5 strains. For Paratyphi B: *U* = 25.54 ± 4.41 *μ*m s^−1^, average from 5 strains. All 10 strains were examined at pH7	[128, 129, 133, 151]
*Salmonella typhi*	(2 − 3)	(0.6 − 0.7)	Peritrichous flagella. *N* = 6, *L* = (9.16 − 11.76)^⋆†^, *n*_*w*_ ≈ (3 − 4.5)^†^, λ = 2.293 ± 0.061^‡^, *h* = 0.35^⋆†^.	25.11 ± 0.46^♢^	^†^Watson’s strain. ^‡^Average from 12 strains. ^♢^Average from 5 strains at pH7. Temporal variation of the swimming speed and effect of temperature over motility were studied.	[128, 129, 131, 133, 151]
*Salmonella typhimurium*	1.4 ± 0.3	(0.5 − 0.73 ± 0.02)	*N* = 4.9 ± 3^†^, *L* = 5.7, *n*_*w*_ ≈ 3, λ = 2.35 ± 0.091^‡^, *h* = 0.18 ± 0.03^†^.	31.7 ± 11.9(18.4 − 55)^♢^	*ω* = 112 at 25°C. ^†^Strain SJW3076. ^‡^Average from 10 strains. ^♢^Average of all values we registered. For the wild type strain *U* = 18.4 ± 8.8 *μ*m s^−1^.	[21, 30, 128, 131, 133, 139, 151, 153, 179]
*Sarcina ureae*	(1.97^⋆^ − 4)		Peritrichous flagella. *L* = (13.3 − 15.7)^⋆^, *n*_*w*_ ≈ (2 − 5), λ_short_ = 1.639 ± 0.0054, λ_long_ = 3.193 ± 0.0048, *h* = 0.37^⋆^.	(18.75^†^ − 28.1^‡^)	Occurs in packets of 8 cocci with one flagellum per cell. ^†^ *η* = 1.16. ^‡^Strain ATCC 13881 at 20°C.	[25, 128, 130, 131, 135]
*Selenomonas ruminantium*	3.98^⋆^	1.17^⋆^	*N* = 6 ± 1.4, Three configurations of flagella were observed in function of pH and salt concentration: Coiled form: Left-handed helix with λ ≈ 0, *h* = 0.965, when pH = (5 − 8) in the absence of salt. Normal: Left-handed helix with λ = 4.7, *h* = 0.965, when pH >8, for every salt concentration. Large Curly: Right-handed helix with λ = 4.84, *h* = 0.93, when pH <5. For pH <3 the flagella were disintegrated.	16 ± 6	*S. ruminantium* subsp. lactilytica TAM6421 (NBRC103574) grown anaerobically at 37°C.	[30]
*Serpens flexibilis*	(8 − 12)	(0.3 − 0.4)	Bipolar and lateral. *N* = (4 − 10) per tuft, *L* = (15 − 30), λ = 0.88, *h* = 0.29.	(1.11^†^ − 16^‡^)	^†^Strain PFR-1 at 30°C with 0.5% agar. ^‡^Strain PFR-3, *η* = 6.5.	[152, 181]
*Serratia marcescens*	1	0.5	Peritrichous flagella. *N* > 4, *n*_*w*_ ≈ 1.5, λ = (0.965 ± 0.037 − 2.591 ± 0.108), *h* = 0.09.	(33.7^†^ − 42.6^‡^)^×^	^†^ *η* = 1.16. ^‡^ *η* = 4.7.	[19, 25, 128, 129, 131]
*Shewanella frigidimarina*		0.226 ± 0.057		24.23 ± 3.82 (max 65)	Mean run time = (0.13 − 0.21) s with acceleration = (86 − 147)*μ*m s^−2^.	[120, 182]
*Shewanella pealeana*	(2 − 3)	(0.34 − 0.6)	*N* ≥ 3 polar flagella.	26.9 (max 55)	Mean run time = 0.15 s with acceleration = 109*μ*m s^−2^.	[120, 183]
*Spirillum gracile*^†^	(5 − 10.9^⋆^)	(0.25 − 0.43^⋆^)	Bipolar flagella. *L* = (1.55 − 4.3)^⋆^, λ = (2 − 3.5)^⋆^, *h* = (0.24 − 0.34)^⋆^.	(28.1^‡^ − 34^♢^)	Strain D-5 observed at 22-23°C. Geometry comes from strains D-2 and D-3. ^†^Helical-shaped. ^‡^ *η* = 1. ^♢^ *η* = 2.	[152, 184]
*Spirillum serpens*^†^	8.2 (3 − 35.17^⋆^)	(1 − 2.34^⋆^)	Bipolar flagella. *N* = 2 × (10 − 15), *L* = 11.43^⋆‡^, *n*_*w*_ ≈ 1, λ = (2.7 − 13^⋆‡^), *h* = (0.55 − 1.37^⋆‡^); λ_*B*_ = 8.2(7.1 − 9.7), *h*_*B*_ = 2.1(1.5 − 3).	(22.8^♢^ − 60^♣^)	^†^Helical-shaped. ^‡^Leifson’s strain. ^♢^ *η* = 1.16. ^♣^ *η* = 2, at 22-23°C.	[25, 131, 133, 152, 185–187]
*Spirillum volutans*^†^	21.74 ± 8.4 (13.5 − 60)^⋆^	(0.97^⋆^ − 2.5)^⋆^	*N* = 75(46 − 200), *L* = (12.43^⋆^ − 17.8^‡^), *n*_*w*_ ≈ 1.1, λ = (6.5 − 12.88), *h* = 1.3^⋆^(*h*^*L*^ = 5.3 ± 0.68^♢^, *h*^*T*^ = 3.68 ± 0.97^♢^); λ_*B*_ = 18.12^⋆^, *h*_*B*_ = 2.08^⋆^.	63^♣^(41.5^♣^ − 85.05)	*ω* = 40, Ω = 13. ^†^Helical-shaped. ^‡^Gray’s strain, the flagellum was not entirelly in the microscopy. ^♢^Unipolar cells of strain ATC 19554 could swim either with Leading or Trailing flagella. At 28°C: *U*^*L*^ = 53 ± 36.7, *ω*^*L*^ = 34.3 ± 21.7; *U*^*T*^ = 81.7 ± 45.8, *ω*^*T*^ = 82.8 ± 55. ^♣^The author was not sure whether it was *S. volutans* species.	[19, 125, 133, 151, 187–190]
*Streptococcus*	(3 − 3.27)	(1.27 − 1.36)	*N* = 3.5 ± 0.2, λ = 3.51^⋆^, *h* = 0.69^⋆^.	16.8 ± 3.7^†^	Ω = 6.7 ± 2.4, *ω* = 88.5 ± 22.16, at 22°C, *η* = 1. Strains smooth-swimming SM197 and wild-type V4051 grown at 35°C, pH7.5. Measurements of swimming speed and bundle frequency are available for *η* hanging from 1 to 10, at 22°C, and in function of the temperature from 10 to 45°C. ^†^For wild-type V4051 in solution with no Ficoll. Other parameters measured: tumble length = 0.18 ± 0.07 s, run length = 1.71 ± 0.9 s, change in direction from run to run = 63 ± 14°, change in direction between runs = 26 ± 8°.	[21, 132, 150, 191]
*Synechococcus*	2	1	Synechococcus swim without the benefit of flagella. Their means of locomotion is not known.	(5 − 25)	Ω ≈ 1.	[192, 193]
*Thiospirillum jenese*	(30 − 100)	(2.5 − 4)	At least 60 polar flagella. *L* = (10 − 12).	(18.75^†^ − 86.5^‡^)	^†^ *η* = 1.16. ^‡^Strain from R. L. Gherna, in a synthetic medium specifically developed for large photosynthetic purple sulfur bacteria by Pfennig and Lippert. Motile behaviour observed in function of viscosity.	[25, 129, 130]
*Thiovulum majus*	7 ± 3(5 − 25)^⋆†^		*N* ≈ 100 peritrichous flagella. *L* = 2.3^⋆^.	330 (150 − 600)	*ω* = (20 − 63), Ω = 6.6 ± 3.4, cells were attached and exerted a force of ≈ 40 pN in the surrounding water. Cells swam in helical paths of *h*_path_ = (5-40), λ_path_ = (40-250), in periods of (0.2 − 1) s. ^†^Different sizes have been reported for different populations of the same species.	[125, 194–196]
*Vibrio alginolyticus*	1.92 ± 0.46	0.8 ± 0.49	*L* = 5.02 ± 1.15, *n*_*w*_ = 2.76(= *L*/Λ), λ = 1.58 ± 0.14, Λ = 1.82 ± 0.16, *h* = 0.14 ± 0.02.	(77^†^ − 147^‡^)	*ω* = (690^†^ − 1660^‡^). Mutant YM42 grown in HI broth at 37°C. ^†^At 25°C. ^‡^At 35°C.	[21, 129, 153, 197, 198]
*Vibrio anguillarum*	(1 − 3.28^⋆^)	(0.5 − 1^⋆^)	Sheathed polar flagella. *L* > 4.5^⋆†^, *n*_*w*_ > 1, λ = 2.73^⋆^, *h* = 0.3^⋆^.	(25^‡^ − 40^♢^)	Strain NB10. ^†^Flagellum not entirely in the picture. ^‡^At 5°C. ^♢^At 25°C	[199–201]
*Vibrio campbellii*				17.5 (max 45)	Mean run time = 0.16 s with acceleration = 109*μ*m s^−2^.	[120]
*Vibrio cholerae*	(1.4 − 3.4)^⋆^	(0.45 − 0.8)	*N* = 1, *n*_*w*_ ≈ 1, λ = 2.43 (or 1.86 × *W*^⋆^), *h* = (0.17 − 0.32^⋆^) (or 0.56 × *W*^⋆^).	64.6 ± 11.2 (50.56 − 99)^†^	^†^Average from all the values registered, for the strains O139, VO18, AP7, AP5, AI1854 and NW13 and 4 unmentioned ones.	[26, 38, 129, 131, 133, 139, 151, 202]
*Vibrio comma*	(1.4 − 4)	(0.5 − 0.80)		200		[129, 194]
*Vibrio parahaemolyticus*	(2 − 2.38^⋆†^)	(0.82 − 0.87^⋆†^)	Cells produce sheathed polar flagellum suited for swimming and numerous unsheathed lateral flagella (under viscous conditions) suited for swarming. Mutants with only one configuration of flagella were also examined: swimming strain RS313 (single polar) and swarmer strain ML34 (lateral). *L* = (1.83^†^ − 5.7)^⋆^, λ_polar_ = 1.16^⋆^, *h*_polar_ = 0.15^⋆^	(15 − 60)^‡^	The monopolar flagellum had its efficiency reduced with an increase in viscosity while cells with only lateral flagella were slower both in high and low viscosities. ^†^Strain H-926, cell outside swarming zone ^‡^Wild-type strain B22 with both lateral and polar flagella	[71, 203, 204]
*Vibrio splendidus*		0.26		19.4 (max 45)	Mean run time = 0.17 s with acceleration = 91 *μ*m s^−2^.	[120]

**Table 5:** Data for swimming Spirochaetes and *Spiroplasma*.

Species	Geometry			Kinematics		References
	*B*	*W*	Helicoidal body	*U*	Notes	
*Borrelia burgdorferi*	(4 − 30)	(0.2 − 0.33)	*N* = 2 × (7 − 11), *n*_*w*_ = (3 − 10), λ_*B*_ = (3.01 ± 0.18 − 3.29 ± 0.07), *h*_*B*_ = (0.41 ± 0.02 − 0.77 ± 0.03).	(4.25 − 6.8 ± 2.4^†^)	*ω* = 10.2, *c* = 34.24. ^†^In BSK-II medium with no Ficoll (*η* ≈ *η*_water_). The torque was estimated to be 2700 pN nm.	[167, 205–209]
*Brachyspira hyodysenteriae*			*h* = 0.43 ± 0.03, λ = 2.84 ± 0.1.	40 ± 4	Wild type.	[210]
*Brachyspira pilosicoli*	5.36^⋆^	0.24^⋆^	λ_*B*_ = 2.2, *h*_*B*_ = 0.32.	5.9 ± 1.7	*ω* = 8.83 ± 3.35. Swine intestinal strain NKf1 grown at 37°C.	[211, 212]
*Cristispira balbianii*	(22.5^⋆^ − 80)	(0.65^⋆^ − 2)	*N* > 100, *n*_*w*_ = (3^⋆^ − 3.5). Parameters out of picture of the veil-like crista (the body loses its regular spiral form on fixation): λ_*B*_ = 6 and *h*_*B*_ = 1.52. ([133] mentions irregular spiral shapes with *n*_*w*_ ≈ (2 − 5), amplitude of 8 *μ*m and depth of 1.6 *μ*m).			[19, 133, 167, 213, 214]
*Cristispira* sp.	45	1.4	*N* > 100, *n*_*w*_ = 3.2. λ_*B*_ = (13.7^†^ − 14.2), *h*_*B*_ = 1.68^†^.	76	([19]: *ω* = 300, *c* = 475); ([57]: $U/(c-U)\approx 0.125\underset{\left(U=76\right)}{\Rightarrow }c=684\underset{\left(\lambda_{B}=14\right)}{\Rightarrow }\omega \approx 50$). ^†^Using the value of *W* to create scale.	[19, 57, 213]
*Leptospira biflexa*	14^†^	(0.14 − 0.15)	When a cell translates, its anterior end is spiral-shaped (*h*_*B*_ = 0.3, λ_*B*_ = 2.7, *W* = 0.18, “S-end”) and the posterior end is hook-shaped (H-end). The anterior S-end is gyrated by the rotation of Periplasmatic Flagella (PF), and the coiled protoplasmic cylinder (PC) (*h*_*B*_ = 0.09, λ_*B*_ = 0.7, *W* = 0.14) rotates in the opposite direction. The S- and H-shapes of the two ends are thought to be determined by the shape of PF.	14.2 ± 2.9^‡^	The anterior S-end is left-handed and gyrates counter-clockwise (*ω* = 74.4 ± 33.6^×‡^), which produces backward motion of the spiral wave. In contrast, the protoplasmic cylinder is right-handed and rotates clockwise (*ω* = 135.6 ± 22.8^×‡^). The posterior H-end is approximately planar and rotates in the same direction as the S-end to allow the cell to translate without twisting (*ω* = 20.97 ± 12.58^×‡^). ^†^ *L*_H-end_ ≈ *L*_S-end_ = 3 and *L*_*PC*_ = 8. ^‡^ *η* = 0.86.	[213, 215]
*Leptospira icterohemorrhagiae*	7.5 (4 − 10)	(0.07 − 0.27)	*N* = 0, λ_*B*_ = (0.3 − 0.6), *h*_*B*_ = (0.25 − 0.45).		At 19-25°C.	[133, 167, 216]
*Leptospira illini*	15 ± 5(10 − 20)	0.12	*L* = 15 ± 5, λ = 0.69 ± 0.04, *h* = 0.120 ± 0.025^†^.		^†^Data for the central part.	[217, 218]
*Leptospira interrogans*	(9.1 − 11.1^⋆^)	(0.139 − 0.163^⋆^)	Right-handed helix with *n*_*w*_ = 25, λ_*B*_ = (0.34^⋆^ − 0.392), *h*_*B*_ = (0.06^⋆^ − 0.085).	30^†^	Geometry comes from picture of serovar *patoc* Patoc 1. ^†^ *η* = 300.	[219–221]
*Leptospira* spp.	9 (5 − 15)	(0.1 − 0.2)		(15.3^†^ − 29.8^‡^)	*L. interrogans* and *L. icterohemorrhagiae*. ^†^ *η* = 3.44. ^‡^ *η* = 530.	[167, 213, 222]
*Spirochaeta aurantia*	15 (5 − 50)	0.3	λ_*B*_ = 2.5.	(16 − 26^†^)	^†^ *η* = 1.	[167, 223–225]
*Spirochaeta halophila*	22 (15 − 30)	0.4	λ_*B*_ = 1.5.	(12^†^ − 16^‡^)	Strain P1 observed at 22-23°C, motility in function of viscosity of media. ^†^ *η* = 1. ^‡^ *η* = 2.	[152, 167, 223]
*Spirochaeta litoralis*	(5.5 − 13)	(0.4 − 0.5)	*N* = (2 − 3), *n*_*w*_ = 1.79 ± 0.72, λ_*B*_ = (8.2 − 9.7), *h*_*B*_ = (0.84 − 1.45).	10.9	([19]: *ω* = 300, *c* = 136.5); ([57]: $U/(c-U)\approx 0.19\underset{\left(U=11\right)}{\Rightarrow }c=69\underset{\left(\lambda_{B}=9\right)}{\Rightarrow }\omega \approx 8$).	[19, 57, 167, 226]
*Spiroplasma citri*	6	(0.16 − 0.23^⋆^)	*n*_*w*_ = 4.1, λ_*B*_ = (0.94^⋆^ − 0.97), *h*_*B*_ = (0.18 − 0.238^⋆^).	(0.67 − 1.09^†^)^×^	^†^At 30°C	[19, 167, 227]
*Spiroplasma melliferum*	4.4 ± 0.8 (max7.285)	(0.15 − 0.2)	*n*_*w*_ = (5 − 6), λ_*B*_ = (0.64^⋆^ − 0.87), *h*_*B*_ = (0.1^⋆^ − 0.185).	(1.5^†^ − 3.3 ± 0.2)	*c* = 35.19^†^. ^†^At 30°Cand *η* = 1.147. [228]: The authors identified four consistent modes of cell movements generating motility: *i*) regular extension and contraction within the limits of helical symmetry; *ii*) reversible switching of helical sense, propagated in either direction along the cell and accomplished within ≈ 0.08 s; *iii*) propagating a deformation on one of the helical turns (kink) along the cell, at a speed up to ≈ 40 *μ*m s^−1^ (this appears to be the most important and effective mode of *Spiroplasma* swimming. It is also reported in [73], where the authors precise that the kinks moved along the cell body at a speed of 10.5 ± 0.3 *μ*m s^−1^ relative to the front of the cell, in the opposite direction of movement, the time between kinks being Gaussian distributed with a mean of 0.26 ± 0.07 s); *iv*) random bending, flexing and twitching (equivalent to tumbling). The authors measured average and running velocities for the cell and also the velocity of travelling waves along the cell for several media with different viscosities and at different temperatures.	[73, 228]
*Treponema denticola*	7.7 ± 0.94	0.2 ± 0.02	Flagellar arrangement 2:4:2, λ_*B*_ = 1.23 ± 0.15, *h*_*B*_ = 0.5 ± 0.05.	0^†^(0.015 − 28.1)	Strains ATCC 33520, ATCC35405 and ATCC35404 were examined. ^†^ *T. denticola* are unable to translate unless suspended in a gel-like medium. Swimming speed is strongly dependent on viscosity and temperature, e.g. for ATCC 33520: *U*(*η* = 9.2, 25°C) = 2.53 ± 0.34(1.65 − 4.85), *U*(*η* = 216, 25°C) = 6.31 ± 1.25 (3.33 − 8.63), *U*(*η* = 9.2, 35°C) = 5.48 ± 1.25(3.63 − 8.64), *U*(*η* = 140, 35°C) = 19.31 ± 4.46(11.82 − 28.1).	[8, 167, 229]
*Treponema pallidum*	(18.46 − 23.65)^⋆^	0.59^⋆^	*N* = 2 × (2 − 4), *n*_*w*_ ≈ (8 − 10)^⋆^, λ_*B*_ = 1.56 ± 0.04, *h*_*B*_ = 0.28 ± 0.01.	1.9 ± 0.2	In CMRL medium with no Ficoll (*η*_water_). The torque was estimated to be 800 pN nm.	[209]
*Treponema primitia*	32.31^†^	0.35	*N* = 2, *n*_*w*_ ≈ 2, λ_*B*_ = 2.5, *h*_*B*_ = 0.6. The cells looked like rigid helices at all times.	12	*ω* = 16.67. ^†^Estimated using the pitch and radius of a helix with two complete turns.	[230]
*Treponema socranskii*	(6 − 15)	(0.16 − 0.18)	Flagellar arrangement 1:2:1.	(0.1 ± 0.03^†^ − 0.56 ± 0.22^‡^)	Strain 35536 grown at 42°C. ^†^ *η* = 2.1. ^‡^ *η* = 88.	[167, 229, 231]
*Treponema vincentii*	6.31^⋆^(5 − 16)	(0.2 − 0.25)	λ_*B*_ = 1.3, *h*_*B*_ = (0.2 − 0.3).	(0.17 ± 0.11^†^ − 0.70 ± 0.25^‡^)	Strain 35580 grown at 42°C. ^†^ *η* = 2.1. ^‡^ *η* = 88.	[167, 229, 232]

**Table 6:** Data for swimming Archaea.

Species	Geometry			Kinematics		References
	*B*	*W*	Flagella	*U*	Notes	
*Halobacterium halobium*	(2^†^ − 5^‡^)		Cells can have either a mono or bipolar tuft of flagella and display “Super flagella”. *L* = 3.85 ± 0.1 (*L*_super_ = 22 ± 2), λ = 1 ± 0.02, *h* = 0.36 ± 0.09,	(1.59 ± 0.39^♢^ − 2.94 ± 0.34^♣^)	Strains wild-type NRL, M407, M416 and Flx37 were examined. ^†^Monopolar flagellated cells. ^‡^Bipolar flagellated cells. ^♢^Counterclockwise rotation of flagellar bundle. ^♣^Clockwise rotation of flagellar bundle.	[233, 234]
*Halobacterium salinarum*	2.6 ± 0.5(1.6^⋆^ − 10)	0.43 ± 0.07 (max1)	Cells can show either a mono- or bipolar tuft of flagella. *L* = 4.3 ± 1, λ = 2.1 ± 0.2, *h* = 0.22 ± 0.03.	3.3 ± 0.9 (max10)	*ω* = 23 ± 5, Ω = 2.9 ± 2.5. Estimatedpower and torque required to rotate the archaellum are 7.710^−18^ W and 50 pN nm, respectively. Temperature range of swimming: 20 − 65°C.	[76, 133, 147, 235]
*Methanocaldococcus jannaschii*	1.5		Polar bundle of flagella. *L* < 5, *n*_*w*_ = 2.	380 ± 40(max 589)	Temperature range of swimming: 20-90°C (optimal growth at 85°C). In zigzag movement, *U* = (50 − 100).	[147, 236, 237]
*Methanocaldococcus villosus*	(1 − 1.66^⋆^)		*N* ≈ 50, *L* = 3.08^⋆†^	287 ± 36(max468)	Temperature range of swimming: 50-90°C (optimal growth at 80°C). In zigzag movement: *U* = (80 − 120). ^†^Estimate: it was very hard to distinguish precisely.	[147, 238]
*Methanococcus maripaludis*	(1.1 − 1.5)		*N* ≈ 25, *L* = (1.93 − 2.16), λ = 0.97(0.8 − 1.12)^⋆^, *h* = 0.15(0.077 − 0.2)^⋆^.	25 ± 3.4 (max 45)	Temperature range of swimming: 20-60°C (optimal growth at 37°C). In zigzag movement: *U* < 10.	[147, 236, 239, 240]
*Methanococcus voltae*	(1.5 − 2)		*N* ≈ 30^⋆^, *L* = 1.71(0.86 − 2.56)^⋆†^.	80 ± 8.5 (max128)	Temperature range of swimming: 20-55°C (optimal growth at 37°C).^†^Average of all registered values	[147, 236, 241]
*Methanospirillum hungatei*	(7.4 − 10)	(0.4 − 0.5)	*N* = (5 − 10) in a polar tuft, *L* ≤ 10, λ = 2 ± 0.2, *h* = 0.34 ± 0.08.	(3 − 10)	Strain GP 1.	[236, 242]
*Pyrococcus furiosus*	2.5		*N* ≈ 50, *L* = 7.	62 ± 7 (max110)	Temperature range of swimming: 70-95°C (optimal growth at 100°C). In zigzag movement: *U* = (30 − 50).	[147, 236]
*Haloarcula quadrata*	(2.36 − 3.07)^⋆^		*L* = 5.18^⋆^, *n*_*w*_ = 4^⋆^, λ = 1.23(0.88 − 1.75)^⋆^, *h* = 0.12(0.11 − 0.15)^⋆^.	0.81 (0.44 − 1.02)^⋆^	Isolate 801030/1 grown at 40°C, identified as *Haloarcula quadrata*. Cells are square or retangular shaped.	[74]
*Sulfolobus acidocaldarius*	(0.9 − 1.5)			45 ± 4.2 (max60)	Temperature range of swimming: 30-80°C (optimal growth at 70°C).	[147, 236]

Eukaryotes have also been divided into three groups. The data for flagellated eukaryotes (121 species) are presented first in Table 7, followed by spermatozoa (60 species) in Table 8 and finally ciliates (93 species) in Table 9.

**Table 7:** Data for swimming flagellated eukaryotes.

Species	Geometry			Kinematics		References
	*B*	*W*	Flagella	*U*	Notes	
*Actinomonas mirabilis*				240	*V* = 75.	[243]
*Alexandrium minutum*	21.7 ± 2.2			(64 ± 23^†^ − 320 ± 84^‡^)	^†^At 12°C. ^‡^At 24°C. Swimming speeds also available at 8, 16 and 20°C.	[244]
*Alexandrium ostenfeldii*	41.1 ± 4.5			(66 ± 19^†^ − 150 ± 34^‡^)	^†^At 12°C. ^‡^At 16°C. Swimming speeds also available at 8 and 20°C	[244]
*Alexandrium tamarense*	26.7 ± 2.6			(108 ± 44^†^ − 255 ± 81^‡^)	A. tamarense isolate SB50 appeared in doublet configuration and swam at *U* = 344 ± 52(max472) (compare: single cells *U* = 238 ± 64(max360)). ^†^At 12°C. ^‡^At 24°C. Swimming speeds also available at 8, 16 and 20°C.	[244]
*Amphidinium britannicum*	51.2 ± 7 (39.5 − 69.9)	36.3 ± 4.6 (30 − 53.9)	*N* = 2 (longitudinal and transverse): *L*_*L*_ ≈ *B*, λ_*T*_ = 3.375^⋆^, *h*_*T*_ = 0.74^⋆^.	72.85(14.1 − 123.3)	Algae from Biologische Anstalt Helgoland, ME76, (Herdman) Lebour, measured with Laser Doppler Spectroscopy.	[245, 246]
*Amphidinium carterae*	16.7^†^(10 − 22)	10^‡^(8 − 13)	*L* > 7.64^⋆^.	81.27^†^(14.1 − 149)	Algae from Biologische Anstalt Helgoland, ME 30, Hulburt, measured with Laser Doppler Spectroscopy. ^†^Average from three ranges registered. ^‡^Average from two ranges registered.	[245, 247–250]
*Amphidinium klebsi*	36.25^†^(20 − 50)	23.25^†^(14 − 30)		73.9	(Synonym of *A. operculatum*) ^†^Average from two ranges registered.	[247–249]
*Apedinella spinifera*	(6.5 − 10)		*L* = (6.5 − 20).	110 (90 − 175)	*V* = 450, *G* = (0.5 − 1).	[93, 251]
*Bodo designis*	(4 − 7)^⋆^	(2 − 4)^⋆^	*N* = 2, *L*_1_ ≈ *B*, *L*_2_ ≈ 2*B*.	39 ± 1(max80)	*V* = 54.	[166, 243, 251, 252]
*Brachiomonas submarina*	(15 − 40)			(77 − 115)	Algae from University of Oslo, Bohlin, measured with Laser Doppler Spectroscopy.	[245]
*Cachonina niei*	21.44^⋆^	13.36^⋆^		227.1^†^(50 − 555.6)	(Syn.: *Heterocapsa niei*) ^†^Average of the values registered.	[253–255]
*Cafeteria roenbergensis*	(1.5 − 2.5)^†^	(1 − 1.5)^†^	*N* = 2 (longitudinal and transverse):*L*_*L*_ = (3 − 5) × *B*, *L*_*T*_ = (1 − 1.5) × *B*.	103.6 (58 − 131.8)^×^	^†^ *C. minuta*.	[251, 256]
*Ceratium cornutum*	(114.5^⋆^ − 130)	(50.7^⋆^ − 77)		(125 − 230.5)	Ω = (0.5 − 0.67), *ω* = 50.	[253, 257–259]
*Ceratium furca*	(35 − 210)	27.5^⋆^	*N* = 2 (longitudinal and transverse). For the longitudinal flagellum: *L* = (1.97 − 2.2) × *W*^⋆^, producing planar sine waves with *n*_*w*_ ≈ 1.5, λ = (0.77 − 1.37) × *W*^⋆^, *h* = (0.177 − 0.25) × *W*^⋆^.	(166 − 222)	Measured at 18-20°C.	[92, 155, 260–262]
*Ceratium fusus*	240^†^(15 − 600)	(15 − 30)	*N* = 2, *L* = 200, helical or planar beat.	(62.5 − 250)	5 measures of speed at 18-20°C. ^†^Average from three ranges registered.	[91, 92, 155, 260, 263]
*Ceratium hirundinella*	(95 − 700)			(194.4 − 277.8)		[155, 253, 263]
*Ceratium horridum*	(200 − 250)	(40 − 60)		(8.3 − 33.3)	At 18-20°C, 4 measures of speed.	[92, 155]
*Ceratium lineatum*	82.1^†^	26.8^†^	λ = 18.6^†^, *h* = 1.3^†^.	36	Ω = 0.63. Cells swim in a helicoidal path with *h*_path_ = 6.5 and λ_path_ = 380, with *ω*_ormal_ = 0.07. ^†^From illustration.	[264]
*Ceratium longipes*	210	(51 − 57)		166	At 18-20°C, 1 measure of speed.	[92, 263]
*Ceratium macroceros*	(40 − 60)			15.4	At 18-20°C, 1 measure of speed.	[92, 155]
*Ceratium tripos*	158^†^(79.56^‡^ − 225)	(68.1^‡^ − 157.1)	*N* = 2 (longitudinal and transverse). For the longitudinal flagellum: *L* = 224 ± 27, *n*_*w*_ = 2.27 ± 0.33, λ = 74.3 ± 9.6, *h* = 14.2 ± 2.3. Helical or planar beat.	121.7 ± 26.8 (69.4 − 250)^♢^	*ω* = 30.2 ± 2, at 20°C and pH8. ^†^Average of 4 values registered. ^‡^Horn length not included. ^♢^From 6 values registered.	[91, 92, 245, 257, 263, 265]
*Chilomonas paramecium*	32.5 (20 − 40)	(10 − 12)	*N* = 2, *n*_*w*_ = 1.5. Helical beat. Mastigonemes.	132.35 (59.7 − 162.8)^†^	At 25-26°C. ^†^From 4 values registered.	[91, 247, 263, 266, 267]
*Chlamydomonas moewusii*	(9 − 16)	(5 − 12)	*N* = 2, *L* = (12 − 24).	128	Breaststroke swimming with *ω* = (10.5 − 12.2).	[247, 268, 269]
*Chlamydomonas reinhardtii*	(7 − 13)		*N* = 2, *L* = (10 − 12). Breaststroke beat.	92.27 (60 − 200)	Ω = (0.13 − 0.382), *ω* = 53 ± 5. Wild-type *C. reinhardtii* (cc1690).	[119, 247, 270–273]
*Chlamydomonas* sp.	13		*N* = 2, *L* = 35(≈2.5 × *B*), *n*_*w*_ = 1, λ = 6.3, planar DDW or rowing breaststroke.	(61 − 65.4)	*ω* = 8.	[19, 91, 245, 247, 274, 275]
*Codonosiga botrytis*	15	5	*N* = 1, *L* = 30.		*c* = 500, *ω* = 28.	[276–278]
*Crithidia deanei*	7.4 ± 0.2		*L* = 13.1 ± 0.4, *n*_*w*_ = 0.77(= *L*/Λ), λ = 11.7 ± 0.2, *h* = 2.2 ± 0.05.	45.6 ± 1.5	*c* = 466 ± 12, *ω* = 40.5 ± 0.8. Cells were cultured at 28°C, and examined at room temperature, 22°C.	[279]
*Crithidia fasciculata*	11.1 ± 0.3		*L* = 15.1 ± 0.5, *n*_*w*_ = 0.94(= *L*/Λ), λ = 11.6 ± 0.2, *h* = 2.2 ± 0.07.	54.3 ± 2.6	*c* = 680 ± 28, *ω* = 60 ± 2.3. Cells were cultured at 28°C, and examined at room temperature = 22°C.	[279]
*Crithidia oncopelti*	(8 − 8.2)	(2.6 − 3)	*N* = 1, *L* = (17 − 20), λ = 14.4, *h* = 2.4, planar BDW or DDW.	(17 − 20)	*c* = 250, *ω* = 16.8, Ω = (1 − 2). λ, *h* and *ω* are available in function of the pressure of the fluid.	[19, 91, 247, 270, 280–282]
*Crypthecodinium cohnii*				(101 − 144.6)^†^	^†^Data from helical tracking.	[264]
*Diaphanoeca grandis*				40	*V* = 74.	[243]
*Dinophysis acuta*	65	55	*N* = 2, *L* = 65, *h* = 11. Mastigonemes. Either helical or planar beat of flagella.	500	At 18-20°C, 1 measure of speed.	[92, 276]
*Dinophysis ovum*	45	34		160		[95]
*Distigma* sp.	(43.8 − 105.8)		*N* = 2, *L*_1_ ≈ 2*B*, *L*_2_ < *B*/2, DDW			[260, 275]
*Dunaliella* sp.	(8 − 13.5)	(5 − 7.6)	*N* = 2.	(121 − 226)	At 20.5-21.5°C.	[91, 96, 245, 247, 263]
*Euglena gracilis*	(45 − 50)	(9.2 − 15)	*N* = 1, *L* ≈ 45.	100.9^†^(59.7 − 162.8)	*ω* = 41.15, Ω ≈ 1.25^‡^. ^†^Average of three registered values. ^‡^From video.	[91, 247, 263, 266, 283]
*Euglena viridis*	(52 − 64)	(10 − 17)	*N* = 1, *L* = (100 − 128), *n*_*w*_ ≈ 1.5, λ = 35 ± 5, *h* = 6 ± 1, helical DDW.	80 ± 15 (max168)	*c* = (410 − 813^†^), Ω ≈ 1, *ω* = 12 ± 3. ^†^Estimate.	[19, 91, 247, 263, 270, 275, 281, 284, 285]
*Eutreptiella gymnastica*	(17 − 30)		*N* = 2, *L*_1_ = (20 − 32), *L*_2_ = (8 − 13).	240 (200 − 275)	*G* = (2 − 5), *V* = 650.	[93, 251]
*Eutreptiella* sp. R	(40 − 60)	(13 − 17)		(115 − 155)	*G* = (1.5 − 2.5), *V* = (4000 − 5888).	[93, 155]
*Exuviaella baltica*	(9 − 22)			138.9	(Taxonomic synonym of *Prorocentrum balticum*)	[155, 286, 287]
*Giardia lamblia*	(10.4 − 12.1)^⋆^	(7.3 − 8.9)^⋆^	*N* = 8 or four pairs (anterior, posteolateral, ventral and caudal), *L* = (10.6 − 12.5)^⋆^, *n*_*w*_ = 2, λ = (2.73^†^ − 5.5^‡^), *h* = (0.2^‡^ − 0.31^†^).	(12 − 40)	*ω*_a_ = (17-18)^♢^, *ω*_v_ = (8-11)^♢^. ^†^Values obtained from curve fitting. ^‡^Simulated values, for both anterior and posterolateral flagella, *η* = 1. ^♢^The cells were attached to the glass slide.	[288–290]
*Gonyaulax polyedra*	39.2 ± 3.7 (max 48)	33.3 ± 3.5 (max 45)	*N* = 2, either helical or planar beat.	(250 − 278)	Ω = 0.65. *V* = 25700. At 20°C.	[243, 247, 263, 291, 292]
*Gonyaulax polygramma*	(30 − 54.1)	46.2		500		[155, 253, 293]
*Gymnodinium aureolum*				394		[5]
*Gymnodinium sanguineum*	47.6 ± 4	30.9 ± 3.3		(135.4 − 305.6)	Ω = 0.19. *V* = 16700. At 20°C.	[243, 253, 292]
*Gymnodinium simplex*	(7.2 − 14)	(6 − 10)		(234 ± 34^†^ − 879 ± 39^‡^)	Strain CCMP 418. Motile behaviour was studied in the presence of the ciliate predator *Mesodinium simplex*. ^†^Approaching swimming. ^‡^Escaping swimming.	[155, 294, 295]
*Gyrodinium aureolum*	(27 − 34)			(33 − 245)	*G* = (2 − 3). Algae from University of Oslo, Hulburt, measured with Laser Doppler Spectroscopy. *V* = 250.	[93, 245]
*Gyrodinium dorsum*	37.5 ± 4.1	31.3 ± 3	*N* = 2, either helical or planar beat.	324 ± 43.8 (254 − 454)	Ω = (1.5 − 2.32). At 20°C. Swimming speed for cells with: short flagella = 240 ± 47(120 − 316) *μ*m s^−1^, no longitudinal flagella = 147 ± 28.5(93 − 224) *μ*m s^−1^.	[19, 247, 253, 291, 292, 296]
*Gyrodinium dorsum*	34.5	24.5	*N* = 1. Planar beat.	148.35	Ω = 8.2 (This *Gyrodinium* has no helical flagella).	[19, 296]
*Hemidinium nasutum*	26.8 (24.4^⋆^ − 30)	17.1 (15.2^⋆^ − 20)		105.6	Ω = 1.	[253, 258, 297]
*Hemiselmis simplex*	(4 − 6.5)	3	*N* = 2.	260 (200 − 450)	*G* = (7 − 10), *V* = 10.	[93, 155, 251]
*Heterocapsa pygmea*	(12 − 15)	10.02 ± 0.74		(89 − 115.7)	Algae from Biologische Anstalt Helgoland, ME71, Loeblich *et al*., measured with Laser Doppler Spectroscopy.	[245, 298]
*Heterocapsa rotundata*	(10 − 15)	(5 − 10)		(102 ± 34^†^ − 564 ± 14^‡^)	Strain K-483, SCAP motile behaviour was studied in the presence of the ciliate predator *Mesodinium simplex*. ^†^Approaching swimming. ^‡^Escaping swimming.	[155, 294, 299]
*Heterocapsa triquetra*	17^†^			97 ± 2	^†^Equivalent spherical diameter.	[166]
*Heteromastix pyriformis*	(5 − 7)		*N* = 2, *L*_1_ = (4 − 5), *L*_2_ = (1.5 − 2) × *B* (Also *L*_1_ = 3 × *B* and *L*_2_ = 2 × *B*^⋆^).	85 (75 − 100)	*G* = (1.5 − 3), *V* = 13. (Synonym of *Nephroselmis pyriformis*).	[93, 251, 260, 300]
*Hymenomonas carterae*	(10 − 15)			(61 − 113)	Coccolithophorid from Biologische Anstalt Helgoland, ME72, (Braarud & Fagerl.) and from the University of Oslo, (Braarud & Fagerl.), measured with Laser Doppler Spectroscopy.	[245]
*Jakoba libera*				19	*V* = 75.	[243]
*Katodinium rotundatum*	(7.5 − 14)	(6 − 8)	*N* = 2 (longitudinal and transverse flagellum).	370 (300 − 550)	*G* = (5 − 10). Paulmier 1992, Throndsen 1969 and Campbell 1973. *V* = (350 − 530). (Taxonomic synonym of *Heterocapsa rotundata* (Lohmann) G. Hansen)	[93, 243, 253, 301]
*Leishmania major*	12.5 ± 0.3		*L* = 16.4 ± 0.6, *n*_*w*_ = 0.91(= *L*/Λ), λ = 11.9 ± 0.3, *h* = 2.9 ± 0.07.	36.4 ± 2	*c* = 291 ± 4, *ω* = 24.5 ± 0.8. Friedlin strain V, cultured at 28°C, examined at room temperature = 22°C	[279]
*Menoidium cultellus*	45	7	*N* = 1, *L* = 10, *n*_*w*_ = 1, λ = 10 ± 2, *h* = 3 ± 0.5. Helical beat. Mastigonemes.	(80 − 193.5)	*c* = 411.7, Ω ≈ 1, *ω* = 17 ± 3.	[267, 284]
*Menoidium incurvum*	(24 − 26)		*N* = 1, *L* ≈ *B*, *n*_*w*_ < 1. Helical DDW.	50	*c* = 312 (estimate), Ω ≈ 1, *ω* = 12.	[247, 275]
*Micromonas pusilla*	(1 − 3)			90(17 − 100)	Algae from the University of Oslo, (Butcher) Parke & Manton, measured with Laser Doppler Spectroscopy. *V* = 1.5.	[93, 245]
*Monas stigmata*	6		*N* = 2, *L*_1_ = 3, *L*_2_ = 15. Planar beat.	269	*ω* = 47.75. In a 3 mm deep chamber. When between thin slides, one has measured *U* = 10 and *ω* = 19.	[247, 274]
*Monosiga* sp.				25	*V* = 20.	[243]
*Monostroma angicava*	(5.9 ± 0.09^†^ − 7.53 ± 0.05^‡^)	(2.96 ± 0.03^†^ − 3.70 ± 0.03^‡^)	*N* = 2, *L* = (13.21 ± 0.17^†^ − 14.21 ± 0.12^‡^).	(158.4^‡^ − 182.7^†^)	In water at 5°C. ^†^Male gametes. ^‡^Female gametes. The planozygotes swam with velocity *U* = 250.8 at 5°C.	[302]
*Nephroselmis pyriformis*	(4.5 − 5)			(138 − 189)	Algae from the University of Oslo, (N. Carter) Ette, measured with Laser Doppler Spectroscopy.	[245]
*Oblea rotunda*	20	20		420		[95]
*Ochromonas danica*	(6.67 − 10.75)^⋆^	(5.5 − 5.7)^⋆^	λ = 4.5 ± 0.2, *h* = 0.96 ± 0.12. Leading flagellum with mastigonemes, producing DDW.	77 ± 2	*ω* = 59 ± 2. At 20°C and *η* = 1. Data also for *η* = {2.3, 3.7, 5.6, 7.5, 10}.	[28, 303]
*Ochromonas malhamensis*	3		*N* = 1, *n*_*w*_ = 2.8, λ = 7, *h* = 1. Mastigonemes. Planar DDW.	(55 − 60)	*ω* = 68.44. At 18°C.	[19, 91, 285]
*Ochromonas minima*	(3.5 − 6.5)		*N* = 2, *L*_1_ = (1 − 2) and *L*_2_ = *B*/3.	75	*G* = (0.25 − 1.25), *V* = 25.	[93, 251]
*Olisthodiscus luteus*	(15 − 30)			140 (20 − 160)	*G* = (0.5 − 1). Algae from the University of Oslo, N. Carter, measured with Laser Doppler Spectroscopy. *V* = 600.	[93, 245]
*Oxyrrhis marina*	(28.2 − 50.8)		*N* = 2 (longitudinal and transverse).	300 ± 134 (90 − 700)^†^	Ω = 9.3. Cells swim in a helicoidal path with *h*_path_ = 18 and λ_path_ = 108, with *ω*_normal_ = 9.8. Speed increased slightly in the presence of food cells. ^†^Average of 7 registered values.	[5, 260, 264, 304]
*Paragymnodinium shiwhaense*	10.9 ± 0.4 (8.4 − 15.2)^†^	8.6 ± 0.3 (5.2 − 11.6)^†^	*N* = 2 (longitudinal and transverse), *L*_*L*_ = 10.12^‡^, λ_*T*_ = (1.4 − 1.7)^⋆^, *h*_*T*_ = (0.81^⋆^ − 0.92^‡^).	571	^†^For cells growing photosynthetically and starved for 2 days. Cells fed with *A. carterae* were bigger. ^‡^From illustration.	[5, 305]
*Paraphysomonas imperforata*				42	*V* = 212.	[243]
*Paraphysomonas vestita*	14.7^⋆^		*n*_*w*_ > 2, *h* = (1.5 ± 0.3 − 2.6 ± 0.5). Mastigonemes. Complex 3D beat.	70(67.7^†^ − 166^‡^)	*ω* = 49 ± 4. At 20-25°C. When a particle made contact with the flagellum, the pattern of flagellar beat changed to a hooked wave and the frequency increased to 74 ± 9 s^−1^. *V* = 190. ^†^Cells swimming in a circular path. ^‡^Cells swimming in a helical path.	[243, 306, 307]
*Pavlova lutheri*	(5 − 8)			(121 − 131)	Algae from Biologische Anstalt Helgoland, ME52, (Droop) Green, measured with Laser Doppler Spectroscopy.	[245]
*Peranema trichophorum*	55 (20 − 70)	12	*N* = 1, *L* = (40 − 100). Mastigonemes. Tractellar, helical BDW.	20	*c* = 200, *ω* = (5 − 6).	[19, 91, 247, 263, 270, 275, 281, 308]
*Peridinium bipes*	42.9^⋆^	37^⋆^		291	Ω = 4.99. Cells swim in a helicoidal path with *h*_path_ = 17.8 and λ_path_ = 289, with *ω*_normal_ = 1.92.	[264]
*Peridinium* cf. *quinquecorne*	(16 − 22)			1500	*V* = 140000.	[243, 245, 253, 309]
*Peridinium cinctum*	46 (40 − 55)	44		(40 − 200)	Ω = 0.83. Algae from Biologische Anstalt Helgoland, ME24, (O. F. Müller) Ehrenb., measured with Laser Doppler Spectroscopy.	[245, 253, 258]
*Peridinium claudicans*	(50 − 105)	(48 − 75)		215(125 − 333)	*V* = 110000. At 18-20°C, 6 measures of speed. (Taxonomic synonym of *Protope-ridinium claudicans*)	[92, 155, 243, 310]
*Peridinium crassipes*	102^⋆^	77^⋆^		100	*V* = 204000. At 18-20°C, 1 measure of speed. (Taxonomic synonym of *Protope-ridinium crassipes*)	[92, 243, 311]
*Peridinium foliaceum*	30.6 ± 3.3	30.6 ± 3.3		185.2	Ω = 2. At 20°C.	[292]
*Peridinium gregarium*	(30 − 35)	≈*B*	*N* = 2, *L*_*T*_ ≈ 200^†^.	(777.8 − 1805.6)	(Taxonomic synonym of *Bysmatrum gregarium*). ^†^Estimate.	[253, 312]
*Peridinium ovatum*	(54 − 68)			188(125 − 250)	*V* = 110000. At 18-20°C, 2 measures of speed. (Taxonomic synonym of *Protope-ridinium ovatum* Pouchet).	[92, 243, 313, 314]
*Peridinium penardii*	(25.1 − 32.5)^⋆^			417	(Taxonomic synonym of *Peridiniopsis penardii* (Lemmermann) Bourrelly).	[315–317]
*Peridinium pentagonum*	106 (75 − 110)	87.5 (75 − 100)		252 (200 − 333)	*V* = 110000. At 18-20°C, 2 measures of speed. (Taxonomic synonym of *Protope-ridinium pentagonum* (Gran) Balech).	[92, 243, 318, 319]
*Peridinium subinerme*	(40 − 60)	(40 − 50)		(278 − 285)	*V* = 50000. At 18-20°C, 1 measure of speed. (Taxonomic synonym of *Protoperidinium subinerme* (Paulsen) Loeblich III).	[92, 155, 243, 320]
*Peridinium trochoideum*	(20 − 30)	(15 − 23)		(36 − 70)		[253, 316]
*Peridinium umbonatum*	28(25 − 35)	23(21 − 30)		250	Ω = 1.67.	[253, 258, 316]
*Phaeocystis pouchetii*	(4.5 − 8)		*N* = 2, *L* = 1.5 × *B*.	(21 − 155)	Algae from Biologische Anstalt Helgoland, ME64, (Harlot) Lagerh., measured with Laser Doppler Spectroscopy.	[245, 251]
*Polytoma uvella*	18.25^†^(15 − 30)	(9 − 20)	*N* = 2, *L* ≈ *B*, λ = 15, *h* = 2.9^⋆^. Planar DDW and rowing breaststroke beating.	103.7^†^(74.8 − 127)	*c* > 312, Ω = (3 − 4), *ω* = 11.7^†^ (7 − 20). At 20-22°C. ^†^Average of the different values registered.	[19, 91, 247, 263, 274, 275, 321, 322]
*Polytomella agilis*	(9.8 − 15)	(4.9 − 9)	*N* = 4, *L* = (8 − 9), Planar DDW and rowing breaststroke.	(80 − 220)	*c* = (90 − 450), *ω* = (7 − 33). At 20-22°C.	[19, 91, 247, 270, 322, 323]
*Poteriodendron* sp.			*L* = 35, λ = 4, *h* ≈ 2. Planar beat.	Sessile	*ω* = 40. At 20°C.	[281]
*Prorocentrum mariae-lebouriae*	14.8 ± 1.7	14.8 ± 1.7		(83 − 171.3 ± 27.8)	Ω = 3. At 20°C.	[245, 257, 292]
*Prorocentrum micans*	(40 − 50)			117.55^†^(47.2 − 611)	*V* = 34000. ^†^Average of 6 registered values.	[243, 245, 253]
*Prorocentrum minimum*	15.1 ± 0.3 (max20)	11.8 ± 0.8	*N* = 2 (longitudinal and transverse): λ_*L*_ = 12.22 ± 0.81, *h*_*L*_ = 1.31 ± 0.2, *h*_*T*_ = 1.14 ± 0.14.	107.7 ± 54.6	Ω = 1.12 ± 0.23, *ω*_*L*_ = 65.9 ± 9.4, *ω*_*T*_ = 36.1 ± 15.2. Algae from Biologische Anstalt Helgoland, ME 3, Pavillard, measured with Laser Doppler Spectroscopy. Strain NIES-238 cultured in ESM medium at 20°C.	[245, 257]
*Prorocentrum redfieldii*	33.2^⋆^	10.28^⋆^	*L* = 13.5^⋆^.	333.3	Bursa (Taxonomic synonym of *Prorocentrum triestinum* J. Schiller).	[324, 325]
*Protoperidinium depressum*	132	116		450		[95]
*Protoperidinium granii*	(35 − 80)	(25 − 56)		86.1	(Ostf.) Balech.	[155, 324, 326]
*Protoperidinium pacificum*	54	50		410		[95]
*Prymnesium parvum*	7.2 ± 0.3	5.4 ± 0.5	*N* = 2 and a haptonema. *L* = 10, *L*_*h*_ = 3.4 ± 0.6.	30	*ω* = 40.	[327]
*Prymnesium polylepis*	9.1 ± 0.8	6.8 ± 0.4	*N* = 2 and a haptonema. *n*_*w*_ = (1 − 2)^⋆^, *L* = 28, *L*_*h*_ = 13.5 ± 1.3, λ = 13.4 ± 2.4^⋆^, *h* = (1.9 − 2.4)^⋆^.	45	*ω* = 33.3.	[327]
*Pseudopedinella pyriformis*	(5 − 8)		*N* = 1 (and usually a pseudopodium), *L* = (3 − 5) × *B*.	105 (90 − 110)	*G* = (0.5 − 1), *V* = 500.	[93, 251]
*Pseudoscourfieldia marina*	(3.2 − 5)			(21 − 63)	Algae from the University of Oslo, (Throndsen) Manton, measured with Laser Doppler Spectroscopy.	[245]
*Pteridomonas danica*	5.54^⋆^		Sine waves with λ = 13.0 ± 1.5, *h* = 2.2 ± 0.5. Mastigonemes.	(120.8 − 238.1)^†^	*ω* = 30 ± 4. At 20-25°C. ^†^For cells swimming in helicoidal paths. For straight: *U* = (64.3 − 69.7); and for circular: *U* = (112 − 134.3).	[306, 328]
*Pyramimonas amylifera*	(18 − 31)		*N* = 4 or 8, *L* = 1.5 × *B*.	(20 − 25)	Algae from the University of Oslo, Conrad, measured with Laser Doppler Spectroscopy.	[245]
*Pyramimonas cf. disomata*	(6 − 12)	(4 − 5)	*N* = 4, *L* = 8^†^.	350 (290 − 420)	*G* = (4 − 6), *V* = 100. ^†^Using the width given to construct scale bars; average of the four flagella.	[93, 251, 329, 330]
*Rhabdomonas spiralis*	(14 − 40)	10	*N* = 1, *n*_*w*_ = 1, λ = 15 ± 3, *h* = 3.5 ± 0.5. Helical beat. Mastigonemes.	120 ± 20	Ω ≈ 1.4, *ω* = 25 ± 5.	[91, 267, 284]
*Rhodomonas salina*	(12 − 17)	6	*N* = 2, *L* ≈ 0.7 × *B*^⋆^.	(153 ± 16^†^ − 950 ± 90^‡^)	Strain from the Marine Biological Laboratory, University of Copenhagen. Motile behaviour was studied in the presence of the ciliate predator *Mesodinium simplex*. ^†^Approaching swimming. ^‡^Escaping swimming.	[5, 155, 251, 294]
*Scrippsiella trochoidea*	25.3 ± 2.4 (max 35)	19.9 ± 2.1		82 (22.2 − 153)	*V* = 3600. Algae from Biologische Anstalt Helgoland, ME64, (Stein) Loeblich, measured with Laser Doppler Spectroscopy.	[243, 245, 292, 324]
*Spumella* sp.	10^†^			25 ± 2	^†^Equivalent Spherical diameter. (Synonym of *Monas* O. F. Müller 1773 and of *Heterochromonas* Pascher 1912)	[166, 251]
*Teleaulax* sp.	(12 − 15)		*N* = 2, *L* ≈ 0.6 × *B*^⋆†^.	(53 − 56)	Behaviour in the presence of the predator *Oxyrrhis marina*. Prior to encounter with predator: *U* = (61 − 76), post-encounter: *U* = (133 − 143). ^†^For *T. acuta*	[5, 251]
*Tetraflagellochloris mauritanica*	(3 − 5)	(2 − 2.5)	*N* = 4, two short flagella (*L*_s_ = (11-12)) and two long flagella (*L*_l_ = (33-36)).	300 ± 35(260 − 350)^†^	*ω* = 10 ± 1^†^. ^†^During forward swimming (the four rear-mounted flagella beat synchronously, uni-directionally, and perfectly phase-locked behind the cell). During backward swimming (the right and left flagella couples beat asynchronously, alternatively and sequentially every 0.4 s), *U* = 102 ± 13(85 − 120) and *ω* = 2.5. The cells are also observed to form colonies of up to 16 cells, for which *U* = 98 ± 11(83 − 115).	[331]
*Trachelomonas volvocina*	25		*L* = 50. Series of helical waves. Mastigonemes.			[267]
*Tritrichomonas foetus*	14.63 ± 1.3	6.73 ± 1	*N* = 4.		Forces and torques have been characterised but no swimming velocity is given.	[332]
*Trypanosoma brucei*	(11.51 − 26)	(1.03 − 3.6)	*L* = 9.04, λ = (1.8 − 3.9), *h* = (1 − 2.5). Planar BDW loops of varying λ and *h*. (Using the figures of [333] one gets *L* = 19.4^⋆^, *n*_*w*_ = (1 − 2), λ = 7.5^⋆^and *h* = 1.95^⋆^).	(5 ± 2^†^ − 8 ± 2^‡^)^♢^	Propagation of kinks = (85 ± 18^†^ − 136 ± 7^‡^) *μ*m s^−1^. Ω = 19 ± 3 flips s^−1^ (each flip ≈180° rotation) at 22°C. ^†^Procyclic form. ^‡^Bloodstream form. ^♢^[333] measured *U* = 18.6 ± 5.9(9.7 − 38)^×^ in persistent swimming and *ω* = 19 when swimming in mouse blood. Motility of the strains ILTat 1.4 and AnTat 1.1 was analysed in the blood from different host mammals. The authors also studied the changes in motile behaviour in response to viscosity changes.	[333–336]
*Trypanosoma congolense*	(11 − 25)	(1.8 − 3.7)	*N* = 1, *L* = (10.47 − 12.76)^⋆^, *n*_*w*_ = (1 − 2), λ = (5.26 − 5.84)^⋆^, *h* = (0.64 − 0.82)^⋆^.	9.7 ± 5.0(1.8 − 26.0)^×^	*ω* = (6 − 9). Motility of the strain IL 1180 and KETRI 3827 was analysed in the blood from different host mammals. The authors also studied the changes in motile behaviour in response to viscosity changes.	[333]
*Trypanosoma cruzi*	20	2	*N* = 1, *n*_*w*_ = 3, λ = 3.5, *h* = 0.5. Planar sine BDW.	(40 − 304)	*ω* = (14 − 23). In blood. Flexible body.	[19, 91, 337, 338]
*Trypanosoma evansi*	22(19 − 24)	2.9(2.1 − 3.7)	*N* = 1, *L* = 18.84^⋆^, *n*_*w*_ = (1 − 2), λ = 9.4^⋆^, *h* = 1.8^⋆^.	16.1 ± 5.5(4.7 − 26)^×^	*ω* = 15. Motility of the strain KETRI 2479 and KETRI 4009 was analysed in the blood from different host mammals. The authors also studied the changes in motile behaviour in response to viscosity changes.	[333]
*Trypanosoma vivax*	23(18 − 29)	3.4(2 − 3.2)	*N* = 1, *L* = 19.4^⋆^, *n*_*w*_ = (1 − 2), λ = 10.2^⋆^, *h* = 2.8^⋆^.	29.5 ± 19.4 (4.5 − 109)^×^	*ω* = (13 − 29). Motility of the strains IL 1392 and IL 2136 was analysed in the blood from different host mammals. The authors also studied the changes in motile behaviour in response to viscosity changes.	[333]

**Table 8:** Data for spermatozoa.

Species	Geometry			Kinematics		References
	*B*	*W*	Flagella	*U*	Notes	
(Cricket)	110 ± 10		*L* = 870 ± 31.6, λ ≈ 20, *h* = 0.9^†^.		*ω* = 13.3 ± 3.4^†^. ^†^In basic suspension medium at 18.5 ± 0.5°C and *η* = 1.2.	[339]
(Guinea Pig)	10.86^⋆^	9.68^⋆^	*L* = 108.55 (*L*_midpiece_ = 11.5).	9.48 ± 0.40		[100, 340]
(Rabbit)	(8.06 − 8.51)	(4.59 − 4.98)	*L* = (46 − 49.51) (*L*_midpiece_ = 8.81), λ = 41.6 ± 4.2^†^, *h* = 3.3 ± 0.3^†^.	(101 ± 7^‡^ − 272 ± 14^♢^)	*ω*_shallow_ = 17 ± 0.9, *ω*_deep_ = 18 ± 0.9. New Zealand white rabbit spermatozoa at 37°C. ^†^From tracings, using the values of *B*, *W* and *L*. ^‡^Average path velocity in shallow slide (25 *μ*m) with ampullar fluid. ^♢^Head velocity in deep slide (100 *μ*m) with ampullar fluid. Cells diluted in BO medium were also studied.	[63, 341–343]
(Rat)	20.44	2.93	*L* = (171.1 − 190) (*L*_midpiece_ = 63).	(71 ± 19^†^ − 166 ± 32^‡^)	*ω* = 11.27 ± 3^×^. ^†^Straight line velocity, measured with Computer-Assisted Sperm Analysis (CASA). ^‡^Curvilinear velocity measured with CASA. Average path velocity = 93 ± 29 *μ*m s^−1^, lateral head displacement *A*_*h*_ = 9.7 ± 3.1*μ*m. Values were also obtained with manual tracking.	[97, 100, 341]
(Stallion)	(5.33 − 6.62)	(2.79 − 3.26)	*L* = (40.5 − 57) (*L*_midpiece_ = (8-10.5), *L*_endpiece_ = 2.5^⋆^).	86.7 ± 3.8^†^	^†^In still fluid. The influence of the current velocities of the fluid on the absolute speed of the spermatozoa was also measured.	[344, 345]
*Acipenser baeri* (siberian sturgeon)				(250 − 300)^†^	*ω* = 60. ^†^Activity lasts (2 − 3) min.	[98]
*Aedes* (mosquito)	(7.84^†^ − 8.57^‡^)^⋆^	(4.13^†^ − 5.33^‡^)^⋆^	*L* > 46^⋆†♢^, *h* ≈ 5.		*ω* = (3.4^♣^ − 34^♠^). ^†^ *A. triseriatus*. ^‡^ *A. aegypti*. ^♢^Tail was not entirely shown in picture. ^♣^Large amplitude waves. ^♠^Short amplitude waves.	[102, 346]
*Aleochara curtula* (beetle)	15.4 ± 0.44^†^		*L* = 84.8 ± 12.81, λ = 9.9(7 − 14.5), *h* = 1.3(0.8 − 2.1). Helicoidal DDW.	8.4 (3.7 − 15.2)	*ω* = 19.2(7.1 − 39.2). ^†^ *L*_acrosome_ = 4.9 ± 0.2, *L*_nucleus_ = 10.5 ± 0.4.	[347]
*Asterias amurensis* (starfish)				259 ± 8	Ω = 2.3 ± 0.3. The authors also estimated the torque as 600 pN nm.	[348]
*Bacillus* (stick insect)			Characteristic large and small waves: λ_large_ = (20 − 30), λ_small_ = (6 − 12), *h*_large_ = (9 − 15), *h*_small_ = (3 − 4).	(16 − 100)	*c*_large_ = (20 − 90), *c*_small_ = (40 − 300), *ω*_large_ = (0.9 − 2.8), *ω*_small_ = (7 − 28).	[103]
*Bos* (bull)	8.87^†^(6.77 − 10.2)	4.74^†^(4.2 − 5.4)	*L* = (44.2 − 63.83) (*L*_midpiece_ = (9.7 − 14.8)), *n*_*w*_ ≈ 1, λ = (30.5^⋆^ − 40), *h* = 8(7.1^⋆^ − 11). Cells present a 3D helical or complex (with varying amplitude) flagellar beat.	97 ± 6 (40 − 160)^‡^	*c* = (400 − 700), Ω = 8(7.14 − 9.1), *ω* = 20.57 ± 3.4. ^†^Average of our registered values. ^‡^Cells also happen to swim in circles with velocity between 20 and 100.	[19, 41, 63, 100, 101, 247, 281, 349–353]
*Bufo marinus* (toad)	>7.6^†^	0.69	*L* > 21.51^†^, λ ≈ 20, *h* = 2.88 ± 1.13.	22.12 ± 15.9 (6.9 − 49.2)	*ω* = 11.74 ± 3.2(6.7 − 15.3). ^†^Not entirely comprised in the figure.	[354]
*Campanularia flexuosa* (hydroid)	3.5^†^	0.81^⋆^	*L* = 40.	(150 − 180)	^†^Head and midpiece.	[355]
*Carassius auratus* (goldfish)	4.2 ± 0.06^×^	4.3 ± 0.06^×^	*L* = (30.3 − 52.9)^×^.	109.4 ± 9.8^×^	Results obtained using automated sperm morphology analysis (ASMA) and computer assisted sperm analysis (CASA). The authors investigated the effect of mercury on the motility and morphology of the spermatozoa.	[356]
*Ceratitis capitata* (fly)			Characteristic large and small waves: λ_large_ = 30, λ_small_ = (5 − 8), *h*_large_ = (10 − 20), *h*_small_ = (1 − 2).	16	*c*_large_ = 120, *c*_small_ = 150, *ω*_large_ = (2 − 4), *ω*_small_ = 20.	[104, 105]
*Chaetopterus* (annelid)	(3.4 − 8.15^†^)	(1.7 − 4.56^†^)	*L* = 36, *n*_*w*_ = (1.25 − 1.4), λ = (19.3 − 25.4), *h* = 3.8, 2D beat.	105	*c* = 660, *ω* = 26. *η* = 1.4. ^†^Three images are superimposed, contributing to a lack of precision in measures.	[19, 349, 353, 357, 358]
*Ciona* (tunicate)	(4.1 − 4.74^⋆^)	(1.33^⋆^ − 2.4)	*L* = 47.5, *n*_*w*_ = (1.3 − 1.57), λ = (22 − 32), *h* = (4.3 − 4.7), 3D and 2D beating.	165	*c* = (1070 − 1122.5), *ω* = 35. At 16°C, *η* = 1.4.	[19, 349, 353, 357, 358]
*Colobocentrotus* (sea urchin)	7.17 ± 0.13^⋆^	3.1 ± 0.36^⋆^	*L* = (35.5^⋆^ − 42), *n*_*w*_ = (1.25 − 1.5), λ = 20.9 ± 3^⋆^, *h* = 3.94 ± 0.95^⋆^. 2D beat.	(165.6 − 193.2)	*ω* = 46. At 23-26°C, *η* = 1.8. The authors also studied the movement of ATP-reactivated sperm: *U* = (73.6 − 83.2), *ω* = 32.	[19, 106, 349]
*Columba livia* (pigeon)	16		*L* = 132 ± 11.1 (*L*_midpiece_ = 98.1 ± 11.2). More complex than a helical wave, with consistent angular velocity always CW.			[99]
*Coturnix coturnix* var. *japonica* (quail)			*L* = 208 (*L*_midpiece_ = 161). Irregular beat, decaying towards the end of the midpiece.	(max 50)	Ω = max4 (CW). At 20-23°C.	[99]
*Culex* (mosquito)	(13.7^†^ − 14.6^⋆^)	0.48^†^	*L* > 41.2^†‡^, *n*_*w*_ = 3.3, λ = 15.5, *h* = 2.6.	6.3^⋆^	^†^ *C. pipiens quinquefasciatus*. ^‡^Not entirely in the picture.	[19, 346, 359]
*Culicoides melleus* (midge)	15.7 ± 0.4		*L* = 173.2 ± 1.17 (*L*_midpiece_ = 6.5 ± 0.5). Characteristic large and small waves: *n*_*w*small_ = 16.8, λ_large_ = 54.1 ± 1.1, λ_small_ = 8.7, *h*_large_ = 2.1 ± 0.9, *h*_small_ = 0.8. Planar beating.	8.3	*c* = 80, *ω* = 8.2(max20). At 25-27°C, pH10.1.	[104, 105, 360]
*Cyprinus carpio* (carp)				140^†^	*ω* = 53^†^. Activity lasted 200 s.	[98]
*Dendraster excentricus* (sand dollar)				(95.75 ± 23.8^†^ − 241.5 ± 46.3^‡^)	^†^At 7.1°C. ^‡^At 24.7°C.	[361, 362]
*Dicentrarchus labrax* (sea bass)				120^†^	*ω* = 70. ^†^During (50 − 60) s.	[98, 363]
*Didelphis* (opossum)			3D beat.		At 37°C, swim in pairs.	[341, 364]
*Echinus microtuberculatus* (sea urchin)				120		[365]
*Fugu* (puffer fish)				160		[363]
*Gadus morhua* (cod)	(1.8 − 3.6)^†^	(1.5 − 2.3)^†^	*L* = (51.5^⋆^ − 90.5), λ = 21.6^⋆‡^, *h* = 2.25^⋆‡^.	(48.3 − 201.5)^♢^	*ω* = (52 − 55). ^†^Heads can be elon-gated or round shaped. ^‡^After 14s activation with sea water. ^♢^At 22°C, motility lasted (7 − 800) s.	[98, 363, 366, 367]
*Gallus domesticus* (domestic fowl)			*L* = 82 (*L*_midpiece_ = 4), λ = 24.6 ± 3.6^†^, *h* = 5.9 ± 1.5^†^, dextral helix.	66.5 ± 10.1^†^	*c* = 623.6 ± 131.6^†^, Ω = 14.8 ± 2.9^†^, *ω* = 25.4 ± 4.8^†^. ^†^Rapid, co-ordinated motility at 23°C in standard saline medium. Cells also swam in slow, low amplitude motility.	[99]
*Hemicentrotus pulcherrimus* (sea urchin)				243 ± 15	Ω = 4.8 ± 0.8. The authors also estimated the torque as 900 pN nm.	[348]
*Hippoglossus hippoglossus* (halibut)				(150 − 180)	*ω* = 55. Duration of motility: 110-120 s.	[98, 363]
*Homo* (human)	5.1^†^(4.5 − 6.11)	3.2^†^(3 − 3.45)	*L* = (49.7 − 56.2) (*L*_midpiece_ = (4 − 7)), λ = 32.1 ± 12.7(= *c*/*ω*). 3D beat.	30.8 ± 11.1 (7 − 50)	*c* = 253.8 ± 76.9(91 − 499), *ω* = (7.9 ± 2 − 19.1 ± 2.95). *U* decreases 46% in cervical mucus. Success in fecundation might be directly related to forward swimming speed (*U* ≥ 25 *μ*m s^−1^) and amplitude of lateral head displacement (*A*_*h*_ ≥ 7.5 *μ*m). Authors measured, for 57 ejaculates: *A*_*h*_ = 5.5(2 − 10) *μ*m. ^†^Average of our registered values.	[7, 63, 96, 100, 101, 281, 341, 353, 368, 369]
*Littorina sitkana* (sea snail)	27^⋆†^	1^⋆†^	*L* = 25.4^⋆†^(*L*_midpiece_ = 16^⋆†^).	185(18^‡^ − 200)	Cells swim in a spiral path doing 24 revolutions per second. ^†^From illustration, obtained after superimposing two frames from a film. ^‡^Backward swimming (tail first), which is more frequent in viscous fluids. *U*(*η*) is available.	[370]
*Lygaeus* (milkweed bug)	(4.8 − 5.24^⋆^)	(0.7^⋆^ − 1)	*L* > 29.8^†^, *n*_*w*_ = 2.3, λ = (13 − 14.5^⋆^), *h* = (1.3^⋆^ − 2.1).		*ω* = (110 − 130). ^†^Not entirely in the picture and estimating the end of the head and beginning of the tail.	[19, 359, 360]
*Lytechinus* (sea urchin)	(5.1 − 7.55^⋆^)	(2.9 − 2.97^⋆^)	*L* = 43.5, *n*_*w*_ = 1.45, Λ = 29.9, λ = 24.8^†^ (22.6 − 30), *h* = (4.6 − 4.7). 2D beat.	158	*c* = (854 − 900), *ω* = 30. At 16°C, *η* = 1.4. ^†^Average of all registered values.	[19, 349, 353, 357, 358]
*Megaselia scalaris* (fly)	18.7 ± 0.54	0.16 ± 0.01	*L* = 128.7 ± 4.09. Characteristic large and small waves: *n*_*w*large_ ≈ 1.1, λ_large_ = (68 − 75^⋆^), λ_small_ = 7, *h*_large_ = (9.3 − 10.27^⋆^), *h*_small_ = 0.5.	117.6 ± 29.6^†^	*ω*_large_ = 3.1. ^†^For straight cells as they move in natural fluid (rounded and linear cells could be observed). Rounded cells moved with *U* = 12.7 ± 6. Cells in methyl cellulose tended to be linear and move at *U* = 35.5 ± 10.3.	[105, 371, 372]
*Merluccius merluccius* (hake)	(2.7 − 3.9)^⋆^	3.1 ± 0.5	*L* = (30 − 50) (*L*_midpiece_ = (2.2-2.6)^⋆^), *n*_*w*_ = (0.5 − 4), λ = (12.1 − 20.9)^†^, *h* = (3 − 8)^‡^.	(57 − 130)	[98]: *ω* = (56 − 57). Motility lasted for (4 − 500) s. ^†^The wavelength decreases linearly with the period of swimming from λ(6 s) = 20.9 *μ*m to λ(28.3 s) = 12.1 *μ*m. ^‡^The amplitude *h* remains approximately constant = 8 *μ*m between 6 s to 17 s of activity and then decreases linearly to 3 *μ*m at 28.3 s. [373] measured, after actvation with sea water, *ω* = 53, λ = 12, *h* = 4 and *U* = 82 ± 25. They also have results for 90 s and 180 s after activation and *ω* in function of temperature. [374] reports *U* = (69 − 102) and has values for the amplitude of lateral head displacement.	[98, 366, 373, 374]
*Mesocricetus* (hamster)	(13.8 − 15.2)	(2.51 − 3)	*L* = (176.5^⋆^ − 250) (*L*_midpiece_ = 50.5). 3D beat.	6.75 ± 0.15	*ω* = 7.75 ± 1.6.	[19, 63, 100, 340, 341]
*Monodelphis domestica* (opossum)	17.65^⋆^	8.77^⋆^	*L* > 237.94^⋆^(*L*_midpiece_ = 10.36).	(247 ± 14^†^ − 342 ± 34^‡^)^♢^	^†^For single spermatozoon at 37°C in Minimum Essencial Medium (MEM). ^‡^For paired spermatozoa at 37°C in MEM. ^♢^Straight line velocity measured with Computer-Aided Semen Analysis (values obtained with sperm tracking are also available and values of curvilinear velocity). Lateral head displacement for paired spermatozoa *A*_*h*_ = 5.6 ± 2.1 *μ*m and *A*_*h*_ = 11.4 ± 2.6 *μ*m for single spermatozoa, at 37°C. Increased viscosity reduces straight line velocity for both paired and single spermatozoa, but paired spermatozoa are able to have net displacement whereas single ones moved in tight circles with poor straight line velocity.	[27]
*Mus* (mouse)	(7.24 − 9.44)	(3.2 − 4.48)	*L* = (113.4 − 134) (*L*_midpiece_ = (18.4–26.8^⋆^)), *n*_*w*_ = 1.2, λ = (50 − 65), *h* = 15. 3D beat.		*ω* = 13.2 ± 2.5.	[19, 63, 100, 341, 353]
*Myzostomus* (worm)	30.8 ± 4.55		*L* = 52 ± 2.5. With 9+ 0 axoneme, spermatozoa can swim either with Flagellum foremost (BDW) or Head foremost (DDW) (see superscript): λ^*F*^ = 3.3 ± 2.1, λ^*H*^ = 28 ± 3, *h*^*F*^ = 0.9 ± 0.6, *h*^*H*^ = 1.7 ± 0.6. The form of the helicoidal body also changes according to the configuration of swimming: $\lambda_{B}^{F}=16.3\pm 2.6$, $\lambda_{B}^{H}=16.7\pm 2.4$, $h_{B}^{F}=(1\pm 0.4-3.3\pm 0.9)$, $h_{B}^{H}=(1.4\pm 0.5-3.2\pm 0.7)$.	(20.7 ± 9.8^†^ − 45.4 ± 18.3^‡^)	Ω^*F*^ = 19.8 ± 5.5, *ω*^*F*^ = 17.5 ± 3.5, Ω^*H*^ = 20.9 ± 4.4, *ω*^*H*^ = 18.3 ± 2.9. *M. cirriferum* Leuckart observed at 15 − 21°C in seawater. ^†^Flagellum foremost. ^‡^Head foremost.	[375]
*Oikopleura dioica* (tunicate)	1	1	*L* = 28 (*L*_midpiece_ = 3).	75.61 ± 1.90 (max109.88 ± 1.65)^†^	^†^The authors examined the motile behaviour in a gradient of sperm attractant.	[376, 377]
*Oncorhynchus mykiss* (trout)				220^†^	*ω* = 55. ^†^Activity lasted 30 s.	[98]
*Ostrea* (oyster)	2.6	2.8	*L* = 47, λ = 25.6, *h* = 4.7. 2D and 3D beat.	(163.8 − 169)	*ω* = 43. At 23°C.	[19, 353, 354]
*Ovis* (ram)	10.6	6.2	*L* = 59, λ = 36.5, *h* = 7.3. 2D and 3D beat.	(132.3 − 136)	*ω* = 29. At 35.5°C.	[19, 353, 354]
*Periplaneta americana* (cockroach)	14.85^⋆†^	0.95^⋆^	*L* = 57.75^⋆^, *h* = 6.43^⋆^	(16.1 ± 1.22^‡^ − 53.6 ± 3.1^♢^)	^†^The acrosome measured 2.08. ^‡^At 15 − 16.6°C. ^♢^At 37 − 39°C	[23, 346, 360]
*Polyodon spathula* (paddlefish)				175	*ω* = 50. Activity lasted 50 s.	[98]
*Psammechinus* (sea urchin)	1		*L* = (40 − 45), *n*_*w*_ = 1.25, λ = 24, *h* = 4. 2D beat.	(180 − 191.4)	*c* = (800 − 1000), *ω* = 35(30 − 40), Ω = 3.	[59, 281, 285, 353, 365, 378]
*Psetta maxima* (turbot)			λ = (6.7 − 10.87)^×†^, *h* = (0.5 − 5.33)^×‡^.	220	*ω* = 60. Motility during 200 s, with varying λ and *h*. ^†^λ between 10.15 and 10.87 *μ*m up to 50 s after activation and then decreases linearly to λ(142s) = 6.7 *μ*m. ^‡^The amplitude decreases almost linearly from 5.33 *μ*m at 10.45 s to 0.5 *μ*m at 142 s.	[98, 363]
*Salmo salar* (atlantic salmon)	(3.6 − 5.5)^×^		*L* = (28.2 − 35.7)^×^.	(18 − 127)	Longevity varied between 18 and 72 s. The authors observed that males with longer sperm had shorter-lived gametes.	[379]
*Salmo trutta fario* (trout)				(160 − 164)^†^	^†^At 12.5 − 16°C, 4 s after activation by fresh water. *U*(8 s) = (85 − 91), *U*(16 s) = (24 − 33) and *U*(26 s) = (2 − 5).	[380]
*Scaphirhynchus platorynchus* (shovelnose sturgeon)				200^†^	*ω* = (48 − 50). ^†^Activity lasted 48 − 50 s.	[98]
*Silurus glanis* (wels catfish)				130	*ω* = 35. Activity lasted 90 s.	[98]
*Strongylocentrotus purpuratus* (sea urchin)	5.6^⋆^	2.5^⋆^	*L* = (38 − 41.7^⋆^), *n*_*w*_ = (1 − 1.5), λ = 27.7 ± 2^†^, *h* = (4 − 4.5)^⋆^.	145.3^⋆^	*ω* = 31.1 ± 0.7^†^. ^†^At 18°C and *η* = 1.1. Values of λ and *ω* in function of *η* are available. [349] reports λ = (30 − 31.6) and *ω* = (25 − 31) at 16°C.	[349, 381, 382]
*Sturnus vulgaris* (starling)	10.3		*L* = 73.4.	110^†^(max200)	Cells swam in three different ways: “Twist-drill” motility (TD, large majority of sperm., spin frequency and swimming velocity rose exponentially with temperature). Spin frequency = 42^†^ (max90) *s*^−1^; “Wave” motility (Ω > 30, *U* > *U*_*TD*_, helical flagellar wave with frequency = (3 − 10) s^−1^; “Speedometer-cable” motility. ^†^At 20°C, body temperature.	[99]
*Taeniopygia guttata* (zebra finch)	11.3 ± 1		*L* = 64.1 ± 5.7.		“Twist-drill” motility (see *Sturnus vulgaris*).	[99]
*Tenebrio* (mealworm beetle)	6.2	1.7	*n*_*w*_ = 4. Characteristic large and small waves: λ_large_ = (20 − 30), λ_small_ = (6 − 12), *h*_large_ = (9 − 15), *h*_small_ = (3 − 4). 2D beat.	(16 − 100)	*c*_large_ = (20 − 90), *c*_small_ = (40 − 300), *ω*_large_ = (0.9 − 2.8), *ω*_small_ = (7 − 28).	[19, 103, 104, 359]
*Tripneustes* (sea urchin)					*ω* = 60. At 25°C.	[349]
*Tuhunnus thynnus* (tuna)	2.3^⋆^	1.13^⋆^	*L* = 36.3^⋆^, *n*_*w*_ ≈ 2, λ = 14.83^⋆^, *h* = 1.67^⋆^.	(215 − 340^⋆^)	*c* = (850 − 960)^†^, *ω* = (57 − 65)^‡^. Activity lasted 140 s. ^†^Apparent *c* = 624.3^⋆^; the values were hence obtained by adding the swimming speed. ^‡^Using the obtained values of *c* and λ (The values are in the margin of error given by one of the articles: *ω* = (50 − 70)).	[98, 363]

**Table 9:** Data for ciliates.

Species	Geometry			Kinematics		References
	*B*	*W*	Cilia	*U*	Notes	
*Amphileptus gigas*	808	136		608		[94]
*Amphorides quadrilineata*	138	47		490		[95]
*Balanion comatum*	16^†^		This species has one caudal cilium.	220 ± 10	^†^Equivalent spherical diameter.	[166]
*Balantidium entozoon*	(84.5^⋆^ − 106)	(43.26^⋆^ − 55.6)	Cells can swim either with dexio-symplectic (slow swimming, right handed spiral path, λ_*MW*_ = 3.83^⋆^) or dexio-antiplectic metachrony (fast swimming, left handed spiral path, λ_*MW*_ = 6.32^⋆^), *ℓ* = (3.71 − 4.72).			[24, 61]
*Blepharisma* sp.	350	120	*ℓ* = 7.5, *N* = 7000 (excluding compound cilia), *κ* = 0.1.	600	*V* = 1830000.	[243, 270, 383]
*Cepedea* sp.	333^⋆^	148.5^⋆^	Symplectic metachrony, λ_*MW*_ = 37, *ℓ* ≈ 25^⋆^.		Considering 250× magnification.	[61]
*Coleps hirtus*	(66 − 123^⋆^)	(30 − 72.9^⋆^)	Cilia distributed regularly, *d* ≈ 10. Antiplectic metachronism (similar to *Paramecium*), *ℓ* = 24.7.	686		[61, 94]
*Coleps* sp.	78	35		523		[94]
*Colpidium campylum*	85.4^⋆^	42.5^⋆^	*ℓ* = 8.16^⋆^, *d* = 2.45^⋆^, antiplectic metachronism (similar to *Paramecium*), λ_*MW*_ = (8.67 − 11.4)^⋆^.			[61]
*Colpidium* sp.	79.1	38.6	Dexioplectic metachronism, λ_*MW*_ ≈ 10.			[24]
*Colpidium striatum*	77 ± 4	35.4 ± 2.2		(max 570)	*U*(*T*) and *U*(*η*) are available.	[384, 385]
*Colpoda* sp.	117.7^⋆^	64.96^⋆^	*ℓ* = 10.7^⋆^, *d* = 3.57, antiplectic metachronism (similar to *Paramecium*), λ_*MW*_ = (7.93 − 10.7)^⋆^.			[61]
*Condylostoma patens*	371	102		1061		[24, 94]
*Didinium nasutum*	126^†^(80 − 200)	83.1^†^(60 − 107)	*ℓ* = 12.5, *N* = 1750 divided in 2 circular rows, *κ* = 0.2, dexioplectic metachrony, λ_*MW*_ = 17^⋆^.	1190^†^(464 − 3000)	*V* = 543000. ^†^Average of our registered values.	[24, 61, 94, 243, 263, 270, 383, 384]
*Epistylis* sp.	36.3 ± 4.1^†^	29.5 ± 1.2^†^			*f* = (11 − 12.5). ^†^ *E. daphniae*.	[386, 387]
*Euplotes charon*	(49 − 83)	(34 − 69)		1053	At 19°C, Λ_path_ = 282.	[94]
*Euplotes patella*	(143 − 261)	124 (91 − 156)		1250		[94]
*Euplotes vannus*	82 ± 11	(26 ± 5^†^ − 47 ± 7^‡^)		446 ± 130^‡^	^†^Width. ^‡^Height. ^♢^Straight swimming. The influence of Hg++ on its motile behaviour has been also measured. There is also data of its swimming in microchannels with bent angles. Cells are also reported to walk.	[388, 389]
*Eutintinnus cf. pinguis*	147	24		410		[95]
*Fabrea salina*	184.1^⋆^	120.8^⋆^		(149^†^ − 283^‡^)	^†^At 18°C. ^‡^At 30°C.	[390, 391]
*Favella ehrenbergi*				920	*V* = 150000.	[243]
*Favella panamensis*	238	94		600		[95]
*Favella* sp.	150	65		1080		[95]
*Frontonia* sp.	(282 − 475)	213 (141 − 285)		1632	At 21.5°C, Λ_path_ = 1000.	[94]
*Halteria grandinella*	21.7 ± 2.3 (max 60)	50		533^†^	^†^Cells jumped 8.05 ± 5.23min^−1^ after encounter with rotifer predator *Synchaeta pectinata*, with velocity = 2760 ± 640 (max 3890) *μ*m s^−1^, covering a distance = 370 ± 260(max1300) *μ*m, at 20°C.	[33, 94]
*Kerona polyporum*	107	64	Cells have 6 rows of cirri.	(465 − 488)	Λ_path_ = 222.	[94]
*Koruga* sp.	(300 − 400)	(200 − 300)	*ℓ* = (20 − 30), symplectic metachrony, λ_*MW*_ = (22 − 40).	O(100)		[24]
*Laboea strobila*	100	49		810		[95]
*Lacrymaria lagenula*	42	45		909	At 26°C.	[94]
*Lembadion bullinum*	43	36		415		[94]
*Lembus velifer*	87	17		200		[94]
*Mesodinium rubrum*	(22^†^ − 45^‡^)	38		(6100 ± 1300^†^ − 9600 ± 300^‡^)^♢^	*f* = 60. ^†^Small cells. ^‡^Large cells. ^♢^At 21°C.	[6, 392, 393]
*Metopides contorta*	115	33	Cells have 5 rows of long cilia. Dexioplectic metachrony, λ_*MW*_ = 17.1.	359		[24, 94]
*Mixotricha* sp.	(400 − 500)	(200 − 300)	*ℓ* = 10, symplectic metachrony, λ_*MW*_ = 7.5.		*f* > 5. Cilia organelles are symbiotic spirochaetes.	[24]
*Nassula ambigua*	(118 − 168)	(59 − 79)		2004	At 19.5°C, Λ_path_ = 1185.	[94]
*Nassula ornata*	282	90		750		[94]
*Nyctotherus cordiformis*	139	97.2	*ℓ* = 7^⋆^, symplectic, dexio-symplectic and dexio-antiplectic metachronies were observed, λ_*MW*_ = 26.6.			[24, 61]
*Opalina obtrigonoidea*	363^⋆†^	113.8^⋆^	*ℓ* = 21.63^⋆^, *d* = (1.8 − 7.6)^⋆^, symplectic metachrony.		^†^Not entirely in the picture.	[61]
*Opalina ranarum*	375^†^(200 − 500)	(112 − 300^⋆^)	*ℓ* = 15.35^†^ (10 − 20), *N* = 10^5^, *d* = (0.33 − 3), *κ* = 1.2^†^(1 − 2), symplectic metachrony, λ_*MW*_ = (30 − 50).	50	*f* = 3.6^†^ (1 − 5), *c*_*MW*_ = (20 − 200). ^†^Average of all registered values.	[19, 24, 94, 270, 276, 281, 383, 394, 395]
*Ophryoglena* sp.	252^⋆^(200 − 450)	(92.8 − 104^⋆^)	Dexio-antiplectic metachronism, λ_*MW*_ = 10.33^⋆^(10 − 13).	4000		[19, 24, 61, 396]
*Opisthonecta henneg*	126	75	Dexioplectic metachrony.	1197	*f* = (10 − 36).	[24, 397]
*Oxytricha bifara*	(235 − 329)	94		1210		[94]
*Oxytricha ferruginea*	150	64		400		[94]
*Oxytricha platystoma*	(120 − 140)	(40 − 60)		520		[94]
*Paramecium aurelia*	125 (98 − 390)	31 (21 − 120)		1310 (800 − 2500)	At 21°C, Λ_path_ = 1500.	[94, 398]
*Paramecium bursaria*	126(60 − 200)	57 (30 − 86)		1365 (1000 − 2083)	At 25°C, 3 different strains.	[94, 398]
*Paramecium calkinsii*	120 (70 − 178)	44 (30 − 70)		995(347 − 2437)		[94, 398]
*Paramecium caudatum*	242(140 − 311)	48 (35 − 70)	*ℓ* = 12, *κ* = (0.5 − 11.1), dexioplectic metachrony, λ_*MW*_ = 12.	1476.5^†^(478.7^‡^ − 4500)	*f*_mouth_ = 35.5 ± 3.1^♢^([386] reports *f*_mouth_ ≈ 8 ± 0.1), *f*_anterior_ = 34.5 ± 3.4^♢^, *f*_body_ = 31.4 ± 8.3^♢^, *f*_posterior_ = 15.2 ± 2.3^♢^. Ω = 1.05 ± 0.296^♢^. *V* = 303000. Λ_path_ = 1731, λ_path_ = (500 − 1000), *h*_path_ = (40 − 150). ^†^Average of three values registered. ^‡^At 16.4°C. ^♢^ *η* = *η*_water_. Influence of viscosity and temperature over motility was studied.	[19, 24, 94, 107, 243, 281, 386, 395, 398, 399]
*Paramecium marinum*	115	49	λ_*MW*_ = 10.8.	930	At 19°C.	[94, 398]
*Paramecium multimicronucleatum*	251 ± 18 (168 − 280)	62 (42 − 77)	*ℓ* ≈ 14.2, *d* = (2.56 − 4.2), antiplectic metachronism, λ_*MW*_ = 10.7^†^.	2843(2173 − 4166)	*f* = 32.5 ± 2.5^†^. ^†^At 20°C, *η* = 1 and pH7.2.	[61, 398, 400, 401]
*Paramecium polycaryum*	88(70 − 112)	31 (21 − 50)		1470 (500 − 2500)		[398]
*Paramecium* spp.	210(150 − 250)		*ℓ* = (10 − 12), *N* = 5000, *κ* = (0.25 − 0.5), dexio-antiplectic metachronism, λ_*MW*_ = (7 − 14)^†^.	1000 (750 − 1200)	*f* = 32^†^, *c*_*MW*_ = 350. Increase in viscosity ⇒ decrease in *f* and increased λ_*MW*_. ^†^At 20°C and *η* = 1.	[24, 61, 91, 270, 281, 383]
*Paramecium tetraurelia*	124 ± 20	46 ± 5		784 ± 31(max1376)	*f*_cortex_ = 35 ± 4, *f*_mouth_ = 66 ± 8, *c*_*MW*_ = (461 − 1596). Wild-type cells of stock d4-2 grown at 27°C swimming in 0.2 ml in depression slides maintained at a temperature between 20 and 25°C. There is data available for some mutants too.	[402]
*Paramecium woodruffi*	169 (98 − 222)	62 (42 − 72)	Antiplectic metachrony.	2000(1250 − 2777)	This species could also swim in right-handed spirals with *U* = 609(581 − 666).	[398]
*Porpostoma notatum*	107.7^⋆^	29^⋆^		(1583.4 − 2101)^⋆^		[256]
*Prorodon teres*	175	160		1066		[94]
*Protoopalina* sp.	315^⋆^	92.4^⋆^	*ℓ* = 15.1, symplectic metachrony, λ_*MW*_ = (20.54 − 27.6).			[61]
*Pseudocohnilem-bus pussilus*				320	*V* = 2500.	[243]
*Spathidium spathula*	(172 − 237)	(21 − 43)		526		[94]
*Spirostomum ambiguum*	(950 − 1140)	95	*ℓ* = 8.2, antipletic metachrony, λ_*MW*_ = 8.5.	810	*f* = 30.	[94, 403]
*Spirostomum* sp.	1000	130	*ℓ* = 12, *N* = 10^5^ (excluding compound cilia), *κ* = 0.2.	1000		[270, 383]
*Spirostomum teres*	(300 − 600)	(50 − 60)		640		[94]
*Stenosemella steinii*	83	58		190		[95]
*Stentor coeruleus*	(420 − 637)	(139 − 308)		1500	*f* = (26 − 42), Λ_path_ = 1140.	[94, 386]
*Stentor polymorphus*	208	(15.2 − 152)	*ℓ* = 27.5, *d* = 3.5, dexioplectic metachrony, λ_*MW*_ = 13.	(817 − 957)	*f* = 33, *c*_*MW*_ = 760. Propagation velocity of bend = 1060(max1200) *μ*m s^−1^.	[19, 94, 276, 404]
*Stentor* sp.	(200 − 2000)		*ℓ* = 30, (2 − 3) rows of about 20 closely packed cilia. Dexioplectic metachronism, λ_*MW*_ = 22.43 ± 2.11^†^ (18.6 − 27.5).		*f* = 26.73 ± 7.45^†^ (10.25 − 36.3), *c*_*MW*_ = 577.4 ± 140.7^†^ (282 − 784). ^†^Average of all the values registered, at different temperatures and viscosities.	[24, 281]
*Strobilidium spiralis*	60	50		330		[95]
*Strobilidium velox*	43 ± 9			150 ± 90 (max 480)^†^	^†^Pre-jump velocity. Cells jumped (3.58 ± 2.92min^−1^ at 24°C, 1.67 ± 3.28min^−1^ at 17°C) spontaneously and after encounter with rotifer predator *Asplanchna girodi*. In spontaneous jumps *U* = 7320 ± 1090 (5570 ± 1230) covering a distance of 9090 ± 1950 (12170 ± 1930) *μ*m at 24°C (17°C) in a trajectory 99.56 ± 0.32 (98.53 ± 1.3) % linear. In jumps following encounters *U* = 6950 ± 2100(max 16070) for a distance = 1500 ± 900(max 4410) *μ*m at 17°C.	[33]
*Strombidinopsis acuminatum*	80	30		390		[95]
*Strombidium claparedi*	(64 − 75)	43		3740	At 18°C.	[94]
*Strombidium conicum*	75	43		570		[95]
*Strombidium* sp.	33	25		360		[95]
*Strombidium sulcatum*	(30 − 35)	(20 − 25)		850 (490 ± 17 − 1517^†^)	*V* = 9000. At 20°C. Swimming speeds as a function of the concentration of bacteria available (an increase in concentration reduced the swimming speed). ^†^Average from 6 trackings	[32, 243, 256]
*Stylonichia* sp.	167	86	*ℓ* = 50, 18 cirri (with (8 − 22) component cilia), *d* = 4.5, λ_*MW*_ = 25.5(28 − 40).	(475 − 1000)^†^	*f* = (36 − 59). At 22°C. Cells can also walk with speed = (100 − 2500)*μ*m s^−1^.	[24, 94, 276, 405]
*Tetrahymena pyriformis*	70 (55.7 − 89.77^⋆^)	27.5^⋆^(20 − 45)	*ℓ* = (7 − 14.35^⋆^), *N* = 500 (excluding compound cilia) divided in (17 − 23) columns, *κ* = 0.2, *d* = (2.84 − 6.16)^⋆^, dexio-antiplectic metachrony (similar to *Paramecium*), λ_*MW*_ ≈ 16.2.	480 (451.2 − 500)	*f* = 20.	[19, 24, 61, 270, 383, 406]
*Tetrahymena thermophila*	(46.2 − 47.1)^⋆^	(28.3 − 28.8)^⋆^	*ℓ* = 5.30 ± 0.95^⋆^.	204.5 ± 24.2^×^	*f* = 15.9 ± 3.7. Values for wild-type cell (CU427.4)	[407]
*Tillina magna*	162 (150 − 175)	82 (75 − 90)		2000	At 25°C.	[24, 94]
*Tintinnopsis kofoidi*	100	29		400		[95]
*Tintinnopsis minuta*	40	26		60		[95]
*Tintinnopsis tubulosa*	95	39		160		[95]
*Tintinnopsis vasculum*	82	49		250		[95]
*Trachelocerca olor*	(235 − 300)	(35 − 40)		900		[94]
*Trachelocerca tenuicollis*	432	43		1111	Λ_path_ = 303.	[94]
*Urocentrum turbo*	90	60	2 circular rows.	700	At 28.5°C, Λ_path_ = 333.	[94]
*Uroleptus piscis*	203	52		487	At 22°C.	[94]
*Uroleptus rattulus*	400			385	(Synonym of *Uroleptus lamella*). At 21°C.	[94, 408]
*Uronema filificum*	(23.6 − 27.8)^⋆^	(13.3 − 14.9)^⋆^		1372.7^⋆†^	^†^Tracking of straight swimming.	[256, 409]
*Uronema marinum*	40 (30 − 83.8^⋆^)	(16 − 41^⋆^)	*ℓ* = 15.9^⋆^, *d* = (5.3 − 7.97), antiplectic (similar to *Paramecium*).	(150 ± 130^†^ − 1400 ± 600^‡^)	*V* = 1000. ^†^Inside the food patch cells. ^‡^Outside the food patch cells.	[61, 94, 243, 256, 263]
*Uronema* sp.	25	11.25	*ℓ* = 5, *N* = 200 (excluding compound cilia), *κ* = 0.6.	(1150 − 1200)	*V* = 1600.	[243, 270, 383]
*Uronemella* spp.	(25 − 31.17^⋆^)	22^⋆^	*N* ≈ 100, *ℓ* = 5.38^⋆^.	250	The cells exert a force of ≈50 pN.	[196]
*Uronychia setigera*	64 ± 7^†^	31^⋆^		7347 ± 1170	Helical trajectories have also been characterised. ^†^The body represents 60% of the total length (64) and the transverse cirri 40%.	[410]
*Uronychia transfuga*	118 ± 10^†^	63^⋆^		6406 ± 876	Helical trajectories have also been characterised. ^†^The body represents 70% of the total length (118) and the transverse cirri 30%.	[410]
*Vorticella microstoma*	55(35 − 83)	35(22 − 50)			*f* = (6 − 8). Species in the genus *Vorticella* can live as a free-swimming telotroch and as a sessile stalked trophont. The stalk is reported to be 90(20 − 385) *μ*m in length, and can be contracted at an average rate of (10000 − 20000) *μ*m s^−1^.	[386, 411, 412]
